# Supplementary material for: Angiogenetic Factors in Chronic Subdural Hematoma Development
Source: Diagnostics (Basel). 2022 Nov 14;12(11):2787. doi: 10.3390/diagnostics12112787 (PMC9689028; doi:10.3390/diagnostics12112787)
Supplement: Supplementary file 1 [file diagnostics-12-02787-s001.zip › diagnostics-1877487-supplementary.pdf]

StatTech 2.8.8

© StatTech LLC, Russia, 2020

The software is registered by the Federal Service for Intellectual Property, accession number 2020615715, registration date 29.05.2020

User: Arkady

Email: arkady.neuro@gmail.com

Data analysis: "Angiogenetic factors in chronic subdural hematomas development Oct-03-2022 08-21-54"

Statistical analysis

Statistical analysis was performed using StatTech v. 2.8.8 (Developer - StatTech LLC, Russia).

Quantitative variables were assessed for normality using the Shapiro-Wilk test (when the number of subjects was less than 50) or the Kolmogorov-Smirnov test (when the number of subjects was more than 50).

Quantitative variables following a normal distribution were described using mean (M) and standard deviation (SD), 95% confidence interval (95% CI) for the mean were estimated.

Quantitative variables following non normal distribution were described using median (Me) and lower and upper quartiles (Q1 – Q3).

Comparison of the two groups for a quantitative variable following a normal distribution was performed using Student's t-test if the variances were equal and Welch's t-test in the case of unequal variances.

Mann-Whitney U-test was used to compare two groups on a quantitative variable whose distribution differed from the normal distribution.

The direction and strength of the association between two quantitative variables were estimated using Pearson's correlation coefficient (in case of the normal distribution of variables), The direction and strength of the association between two quantitative indicators were estimated using Spearman's correlation coefficient.

The prognostic model characterizing the dependence of a quantitative variable on predictors was developed using ordinary least squares linear regression.

ROC analysis was used to assess the diagnostic performance of quantitative variables in predicting a categorical outcome. The optimal cut-off value of the quantitative variable at was estimated using the Youden's J statistic.

Table 1 - Descriptive statistics for quantitative variables

Variables	M ± SD / Me	95% CI / Q_1 – Q_3	n	min	max

VEGF (vein) healthy volunteers, M ± SD (pg/ml)	271 ± 41	257 – 286	33	168	358
Age Patients, M ± SD	61 ± 14	49 – 73	8	43	82
Age healthy volunteers, M ± SD	54 ± 15	48 – 59	33	27	79
MMP-9 (vein) healthy volunteers, Me (ng/ml)	453	379 – 486	33	326	499
Angio-2 (vein) healthy volunteers, M ± SD (pg/ml)	2198 ± 248	2110 – 2286	33	1695	2677
PDGF-β (vein) healthy volunteers, M ± SD (pg/ml)	3611 ± 371	3442 – 3779	21	2810	4120
TGF-β1 (vein) healthy volunteers, M ± SD (pg/ml)	10936 ± 411	10748 – 11123	21	10110	11850
VEGF (vein) Patients, Me (pg/ml)	39	17 – 153	8	10	496
VEGF (artery) Patients, Me (pg/ml)	37	25 – 134	7	17	300
MMP-9 (vein) Patients, Me (ng/ml)	692	288 – 844	8	138	2284

MMP-9 (artery) Patients, M ± SD (ng/ml)	596 ± 416	211 – 980	7	139	1205
Angio-2 (vein) Patients, Me (pg/ml)	2608	2292 – 5182	8	1932	7000
Angio-2 (artery) Patients, M ± SD (pg/ml)	3799 ± 2259	1709 – 5889	7	1729	6944
TGF-β1 (vein) Patients, M ± SD (pg/ml)	7462 ± 3497	4539 – 10386	8	3630	12585
TGF-β1 (artery) Patients, M ± SD (pg/ml)	8869 ± 1327	7642 – 10097	7	6660	10260
PDGF-β (vein) Patients, M ± SD (pg/ml)	2372 ± 1371	1226 – 3519	8	840	4420
PDGF-β (artery) Patients, M ± SD (pg/ml)	2683 ± 1042	1719 – 3646	7	1180	3980

We performed a correlation analysis of the association between VEGF (vein) healthy volunteers and Age healthy volunteers.

Table 2 – Results of the correlation analysis of the association between VEGF (vein) healthy volunteers and Age healthy volunteers

Variable	Correlation characteristics		
	r_{xy}	Strength of the association assesed using Chaddock scale	p

VEGF (vein) healthy volunteers – Age healthy volunteers	-0.259	Weak	0.145

A weak correlation negative association between Age healthy volunteers and VEGF (vein) healthy volunteers was estimated.

Observed dependence of Age healthy volunteers from VEGF (vein) healthy volunteers is described by a linear regression equation:

$$Y_{\text{Age healthy volunteers}} = -0.096 \times X_{\text{VEGF (vein) healthy volunteers}} + 79.563$$

With an 1 pg/ml decrease of VEGF (vein) healthy volunteers 0.096 change of Age healthy volunteers should be expected. According to the coefficient of determination R^2 of the resulting model, 6.7% of the observed variance of Age healthy volunteers were explained..

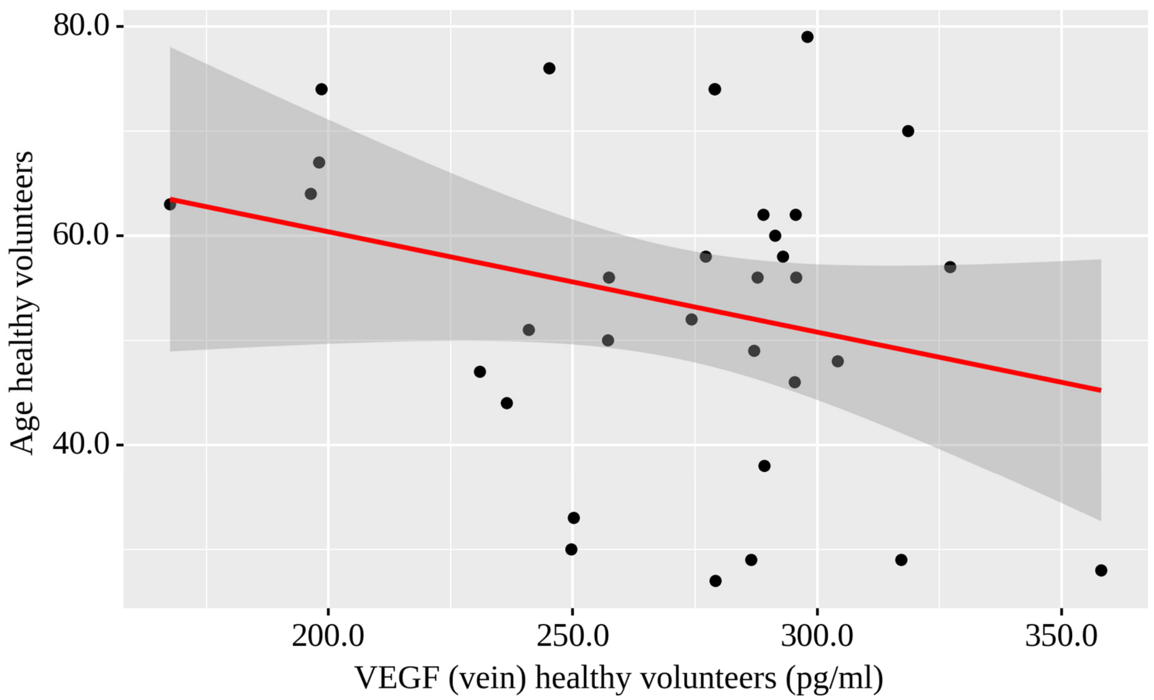

Figure 1 – Regression line characterizing the dependence of Age healthy volunteers from VEGF (vein) healthy volunteers

Analysis of VEGF (vein) healthy volunteers was performed conditioning on Sex Healthy volunteers.

Table 3 – Analysis of VEGF (vein) healthy volunteers conditioning on Sex Healthy volunteers

Variable	Categories	VEGF (vein) healthy volunteers (pg/ml)			p
		Me	$Q_1 – Q_3$	n	
Sex Healthy volunteers	Female	289	279 – 304	13	0.113
	Male	276	249 – 289	20	

When comparing of VEGF (vein) healthy volunteers depending on Sex Healthy volunteers no statistically significant differences were revealed (p = 0.113) *(applied method: Mann-Whitney U-test)*.

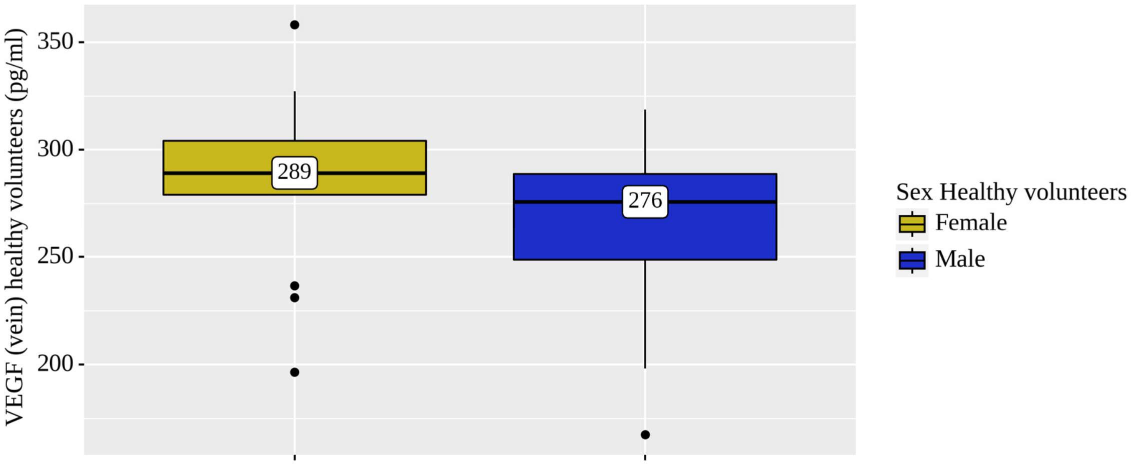

Figure 2 – Analysis of VEGF (vein) healthy volunteers conditioning on Sex Healthy volunteers

When evaluating the dependence of the probability of Male on the VEGF (vein) healthy volunteers using the ROC analysis, the following curve was obtained.

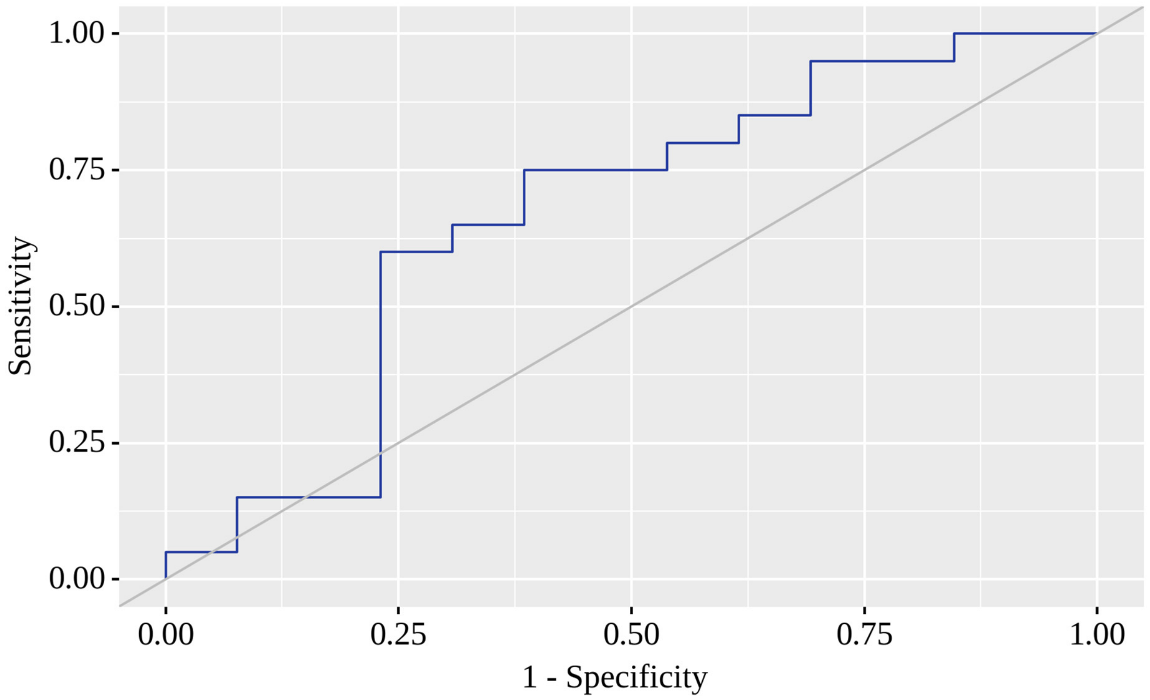

Figure 3 – ROC-curve characterizing the dependence of the probability Sex Healthy volunteers on VEGF (vein) healthy volunteers

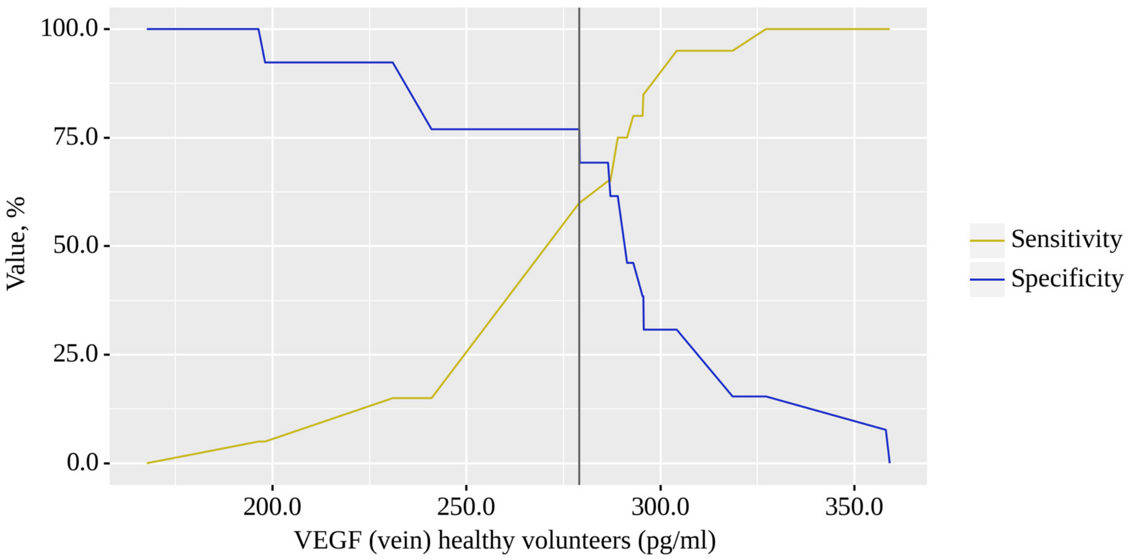

Figure 4 - Analysis of the sensitivity and specificity of Sex Healthy volunteers depending on VEGF (vein) healthy volunteers

Table 4 – Threshold VEGF (vein) healthy volunteers

Threshold	Sensitivity (Se), %	Specificity (Sp), %	PPV	NPV

289	75.0	61.5	75.0	61.5
**287**	**65.0**	**61.5**	**72.2**	**53.3**
286	65.0	69.2	76.5	56.2
279	60.0	69.2	75.0	52.9
279	60.0	76.9	80.0	55.6

The area under the ROC curve comprised 0.665 ± 0.100 with 95% CI: 0.470 - 0.861. The resulting model was not statistically significant (p = 0.113).

The cut-off value of VEGF (vein) healthy volunteers which corresponds to the highest Youden's J statistic is 279.100 pg/ml. If VEGF (vein) healthy volunteers was less than this value, Male was predicted. The sensitivity and specificity of the method were 60.0% and 76.9%, respectively.

Analysis of Age Patients was performed conditioning on Rebleeding on CT scans.

Table 5 – Analysis of Age Patients conditioning on Rebleeding on CT scans

Variable	Categories	Age Patients			p
		Me	$Q_1 – Q_3$	n	
Rebleeding on CT scans	none	55	44 – 69	4	0.564
	rebleeded	59	58 – 66	4	

When comparing of Age Patients depending on Rebleeding on CT scans there were no statistically significant differences (p = 0.564) *(applied method: Mann-Whitney U-test)*.

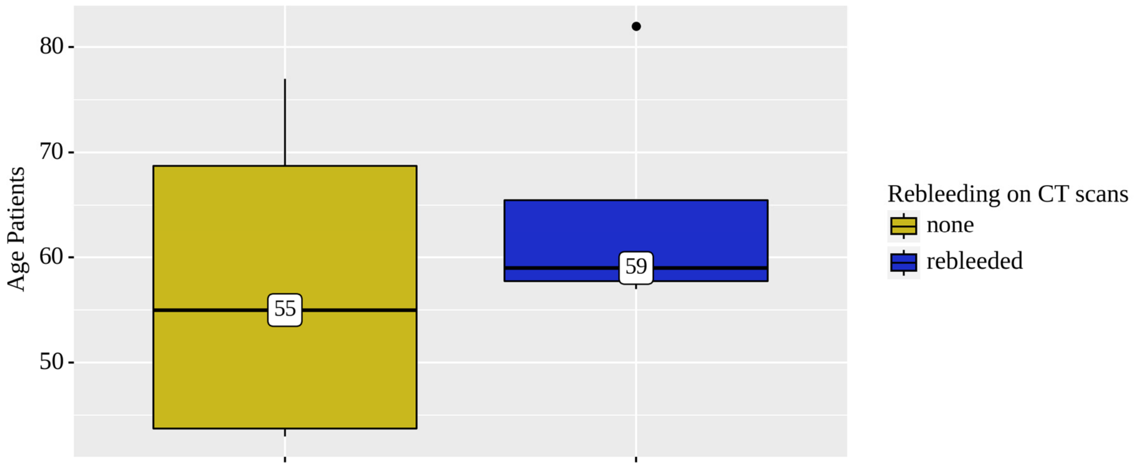

Figure 5 – Analysis of Age Patients conditioning on Rebleeding on CT scans

We performed a correlation analysis of the association between MMP-9 (vein) healthy volunteers and Age healthy volunteers.

Table 6 – Results of the correlation analysis of the association between MMP-9 (vein) healthy volunteers and Age healthy volunteers

Variable	Correlation characteristics		
	ρ	Strength of the association assesed using Chaddock scale	p
MMP-9 (vein) healthy volunteers – Age healthy volunteers	0.084	None	0.642

There was no association between Age healthy volunteers and MMP-9 (vein) healthy volunteers.

Observed dependence of Age healthy volunteers from MMP-9 (vein) healthy volunteers is described by a linear regression equation:

$$Y_{\text{Age healthy volunteers}} = 0.021 \times X_{\text{MMP-9 (vein) healthy volunteers}} + 44.469$$

With an 1 ng/ml increase of MMP-9 (vein) healthy volunteers 0.021 change of Age healthy volunteers should be expected. According to the coefficient of determination R^2 of the resulting model, 0.7% of the observed variance of Age healthy volunteers were explained..

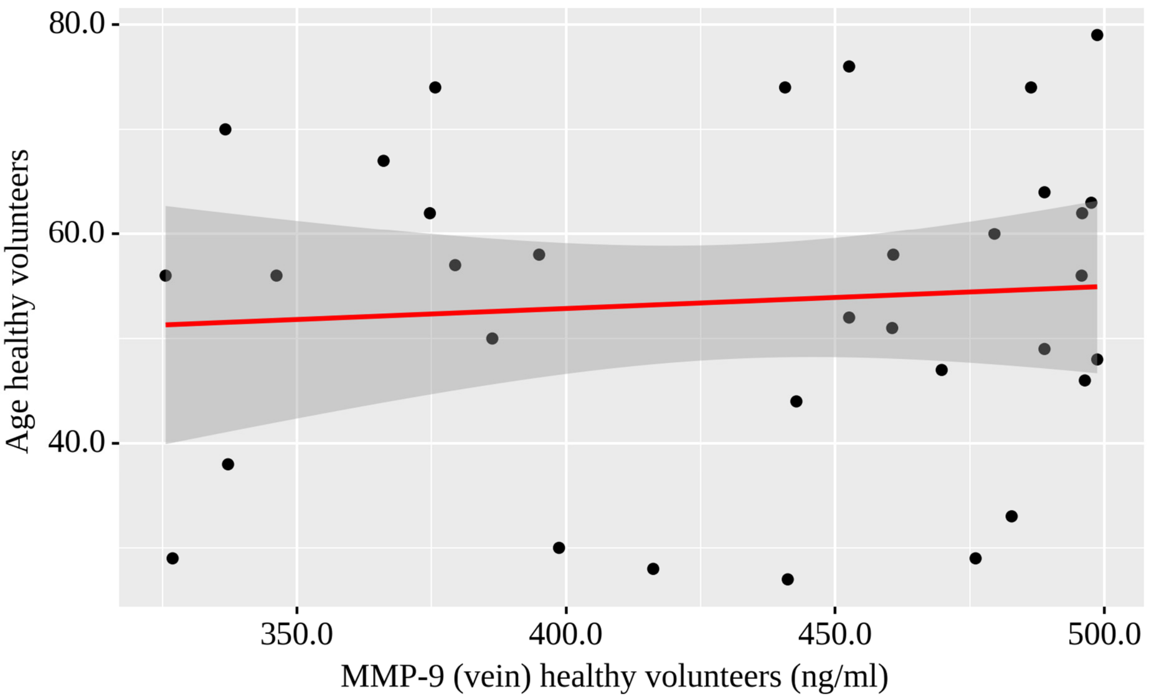

Figure 6 – Regression line characterizing the dependence of Age healthy volunteers from MMP-9 (vein) healthy volunteers

We performed analysis of MMP-9 (vein) healthy volunteers conditioning on Sex Healthy volunteers.

Table 7 – Analysis of MMP-9 (vein) healthy volunteers conditioning on Sex Healthy volunteers

Variable	Categories	MMP-9 (vein) healthy volunteers (ng/ml)			p
		Me	$Q_1 – Q_3$	n	
Sex Healthy volunteers	Female	416	376 – 476	13	0.461
	Male	457	396 – 487	20	

When comparing of MMP-9 (vein) healthy volunteers depending on Sex Healthy volunteers no statistically significant differences were revealed (p = 0.461) *(applied method: Mann-Whitney U-test)*.

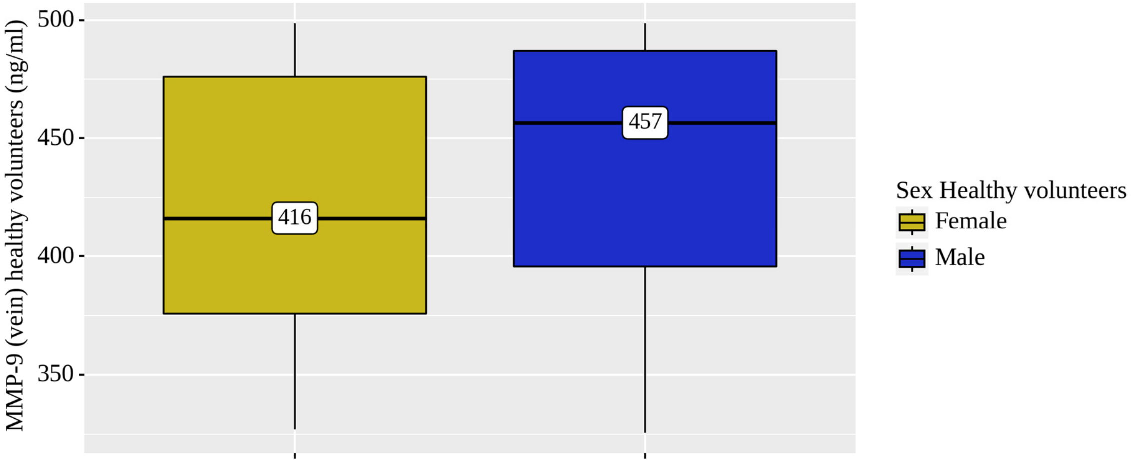

Figure 7 – Analysis of MMP-9 (vein) healthy volunteers conditioning on Sex Healthy volunteers

When evaluating the dependence of the probability of Male on the MMP-9 (vein) healthy volunteers using the ROC analysis, the following curve was obtained.

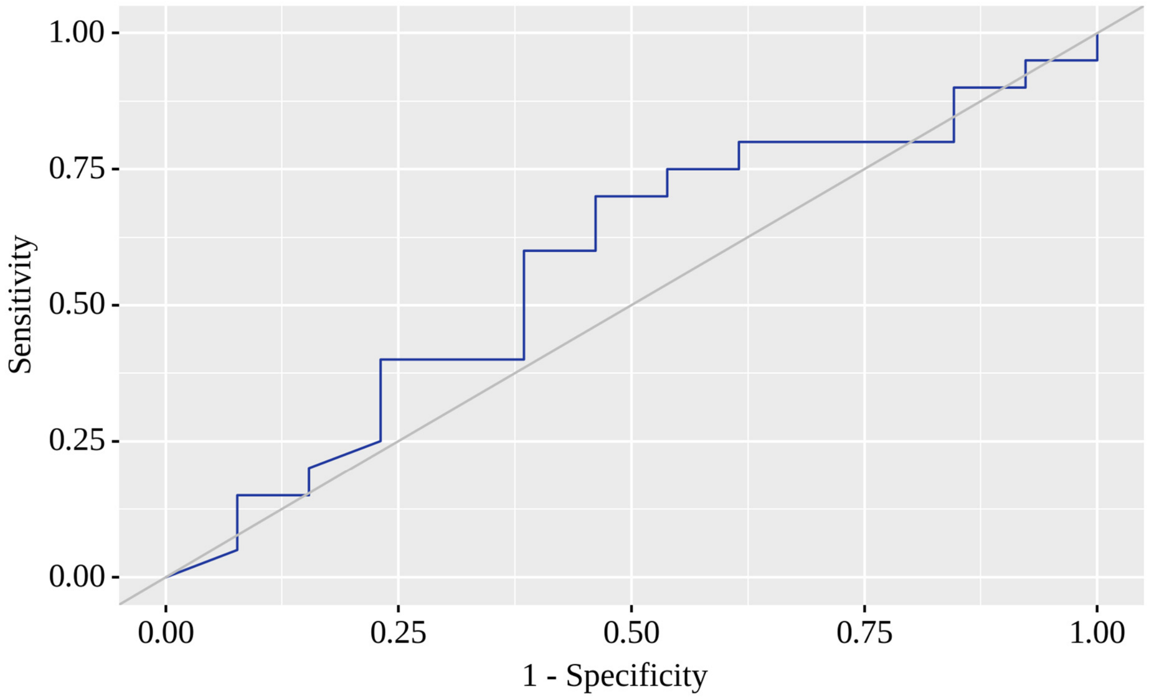

Figure 8 – ROC-curve characterizing the dependence of the probability Sex Healthy volunteers on MMP-9 (vein) healthy volunteers

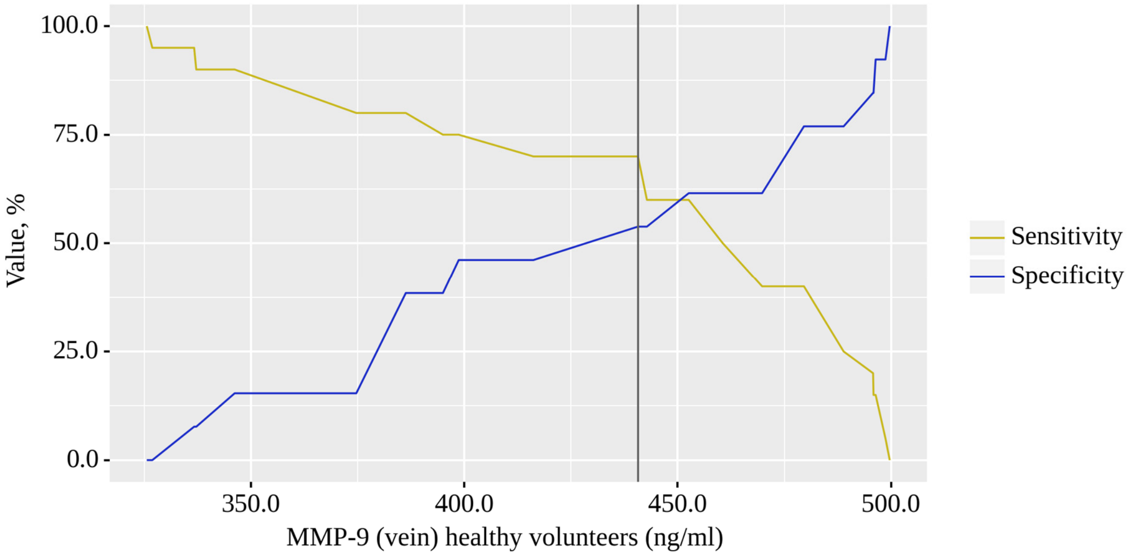

Figure 9 - Analysis of the sensitivity and specificity of Sex Healthy volunteers depending on MMP-9 (vein) healthy volunteers

Table 8 – Threshold MMP-9 (vein) healthy volunteers

Threshold	Sensitivity (Se), %	Specificity (Sp), %	PPV	NPV
461	50.0	61.5	66.7	44.4
**453**	**60.0**	**61.5**	**70.6**	**50.0**
443	60.0	53.8	66.7	46.7
441	70.0	53.8	70.0	53.8

The area under the ROC curve comprised 0.577 ± 0.102 with 95% CI: 0.377 - 0.776. The resulting model was not statistically significant (p = 0.461).

The cut-off value of MMP-9 (vein) healthy volunteers which corresponds to the highest Youden's J statistic is 440.700 ng/ml. If MMP-9 (vein) healthy volunteers was greater than or equal to this value, Male was predicted. The sensitivity and specificity of the method were 70.0% and 53.8%, respectively.

Correlation analysis of the association between Angio-2 (vein) healthy volunteers and Age healthy volunteers was performed.

Table 9 – Results of the correlation analysis of the association between Angio-2 (vein) healthy volunteers and Age healthy volunteers

Variable	Correlation characteristics

	r_{xy}	Strength of the association assesed using Chaddock scale	p
Angio-2 (vein) healthy volunteers – Age healthy volunteers	0.053	None	0.768

There was no association between Age healthy volunteers and Angio-2 (vein) healthy volunteers.

Observed dependence of Age healthy volunteers from Angio-2 (vein) healthy volunteers is described by a linear regression equation:

$$Y_{\text{Age healthy volunteers}} = 0.003 \times X_{\text{Angio-2 (vein) healthy volunteers}} + 46.38$$

With an 1 pg/ml increase of Angio-2 (vein) healthy volunteers 0.003 change of Age healthy volunteers should be expected. According to the coefficient of determination R^2 of the resulting model, 0.3% of the observed variance of Age healthy volunteers were explained..

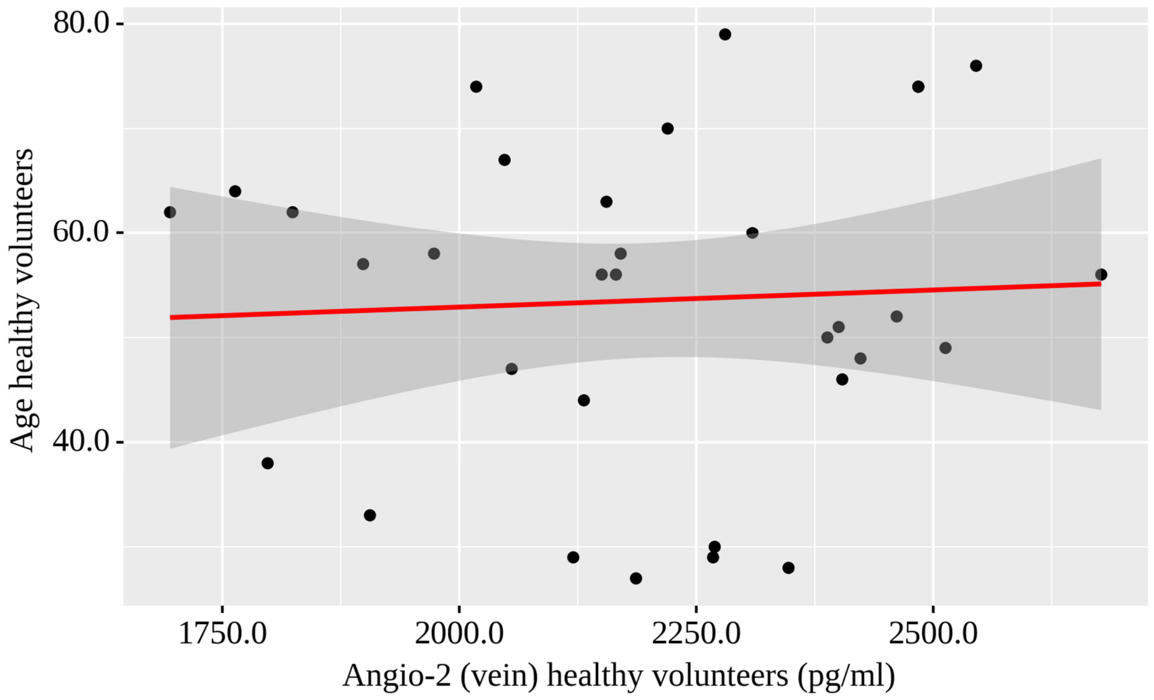

Figure 10 – Regression line characterizing the dependence of Age healthy volunteers from Angio-2 (vein) healthy volunteers

Analysis of Angio-2 (vein) healthy volunteers was performed conditioning on Sex Healthy volunteers.

Table 10 – Analysis of Angio-2 (vein) healthy volunteers conditioning on Sex Healthy volunteers

Variable	Categories	Angio-2 (vein) healthy volunteers (pg/ml)			p
		M ± SD	95% CI	n	
Sex Healthy volunteers	Female	2060 ± 262	1901 – 2218	13	0.008*
	Male	2287 ± 196	2196 – 2379	20	

* – differences are statistically significant (p < 0.05)

According to the presented table, when comparing of Angio-2 (vein) healthy volunteers, statistically significant differences were revealed depending on Sex Healthy volunteers (p = 0.008) *(applied method: Student's t-test)*.

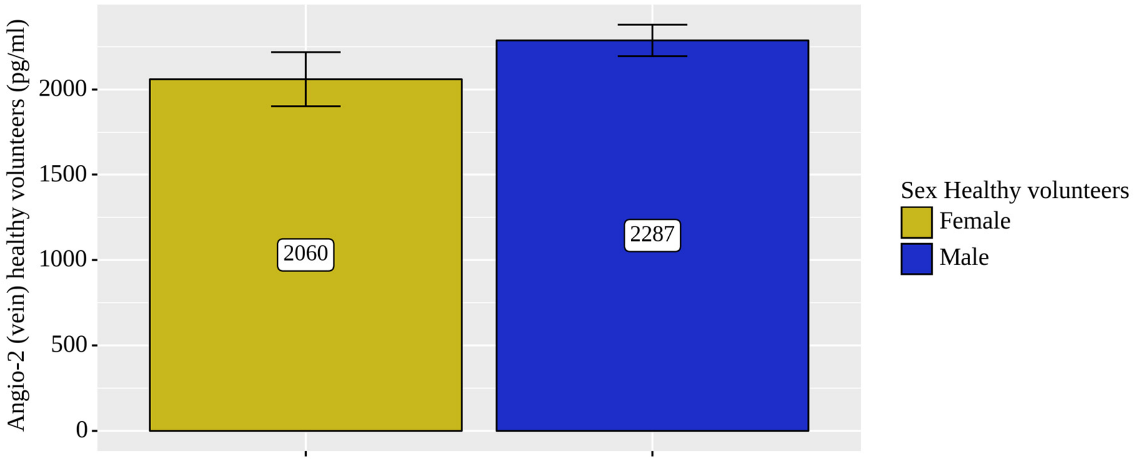

Figure 11 – Analysis of Angio-2 (vein) healthy volunteers conditioning on Sex Healthy volunteers

When evaluating the dependence of the probability of Male on the Angio-2 (vein) healthy volunteers using the ROC analysis, the following curve was obtained.

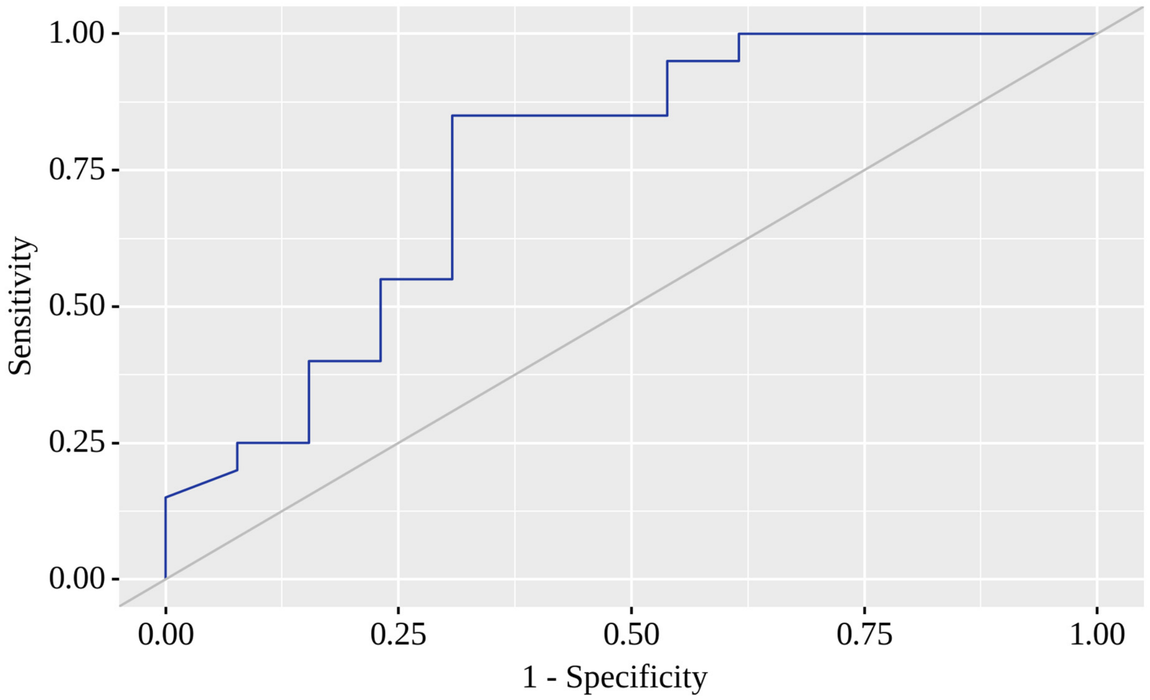

Figure 12 – ROC-curve characterizing the dependence of the probability Sex Healthy volunteers on Angio-2 (vein) healthy volunteers

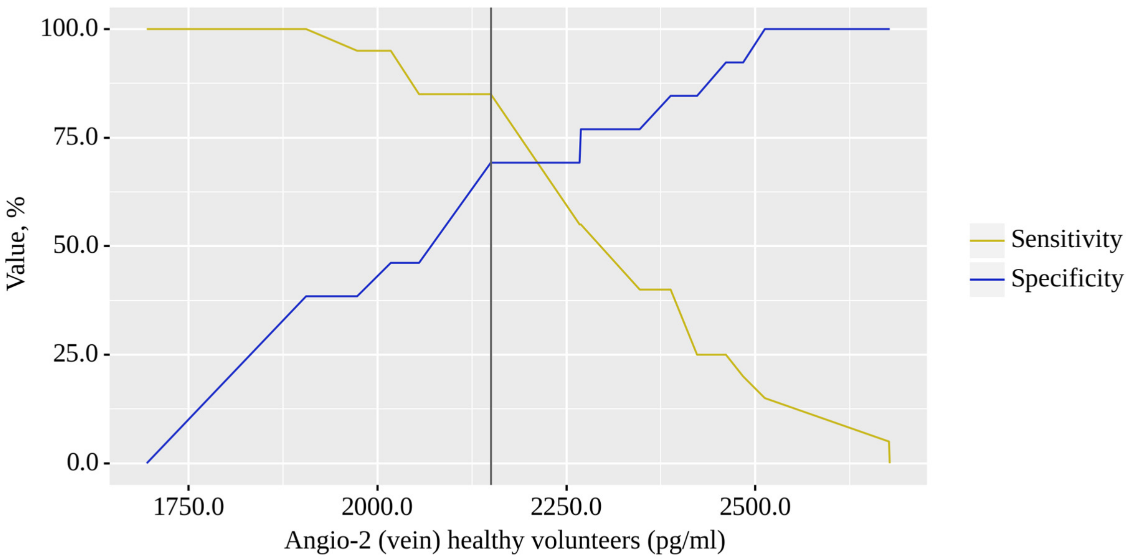

Figure 13 - Analysis of the sensitivity and specificity of Sex Healthy volunteers depending on Angio-2 (vein) healthy volunteers

Table 11 – Threshold Angio-2 (vein) healthy volunteers

Threshold	Sensitivity (Se), %	Specificity (Sp), %	PPV	NPV

2269	55.0	76.9	78.6	52.6
**2268**	**55.0**	**69.2**	**73.3**	**50.0**
2150	85.0	69.2	81.0	75.0

The area under the ROC curve comprised 0.760 ± 0.083 with 95% CI: 0.596 - 0.923. The resulting model was statistically significant ($p = 0.013$).

The cut-off value of Angio-2 (vein) healthy volunteers which corresponds to the highest Youden's J statistic is 2150.000 pg/ml. If Angio-2 (vein) healthy volunteers was greater than or equal to this value, Male was predicted. The sensitivity and specificity of the method were 85.0% and 69.2%, respectively.

Correlation analysis of the association between PDGF-β (vein) healthy volunteers and Age healthy volunteers was performed.

Table 12 – Results of the correlation analysis of the association between PDGF-β (vein) healthy volunteers and Age healthy volunteers

Variable	Correlation characteristics		
	r_{xy}	Strength of the association assesed using Chaddock scale	p
PDGF-β (vein) healthy volunteers – Age healthy volunteers	0.375	Moderate	0.094

A moderate correlation positive association between Age healthy volunteers and PDGF-β (vein) healthy volunteers was estimated.

Observed dependence of Age healthy volunteers from PDGF-β (vein) healthy volunteers is described by a linear regression equation:

$$Y_{\text{Age healthy volunteers}} = 0.015 \times X_{\text{PDGF-β (vein) healthy volunteers}} - 3.297$$

With an 1 pg/ml increase of PDGF-β (vein) healthy volunteers 0.015 change of Age healthy volunteers should be expected. According to the coefficient of determination R^2 of the resulting model, 14.1% of the observed variance of Age healthy volunteers were explained..

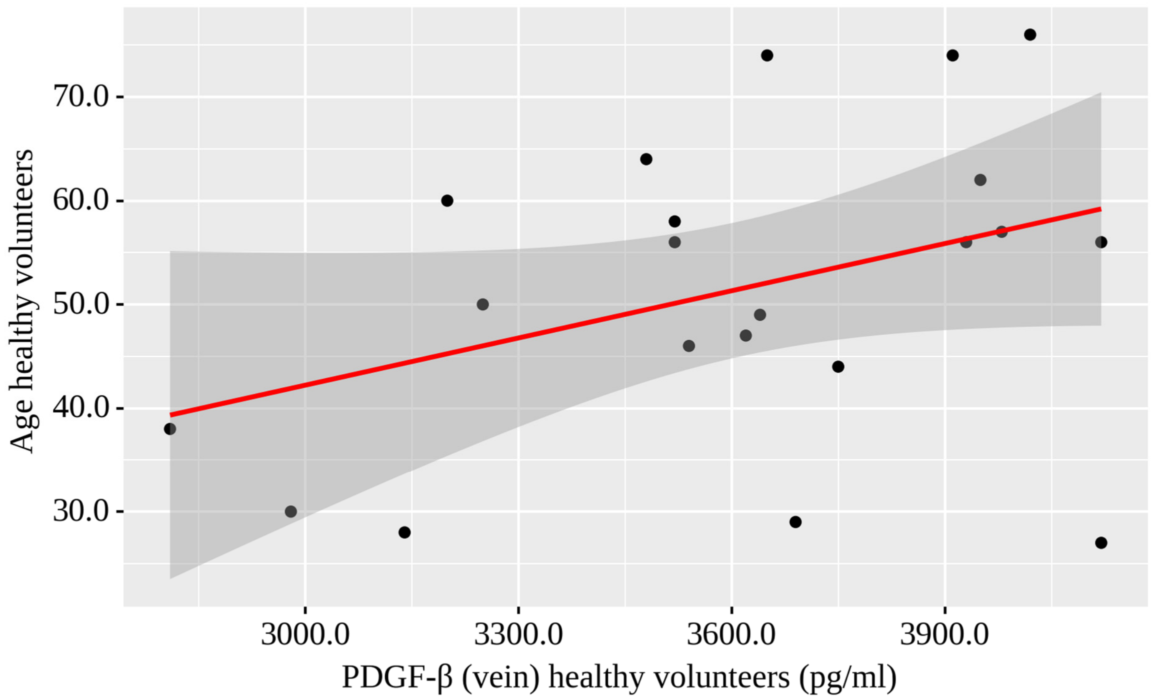

Figure 14 – Regression line characterizing the dependence of Age healthy volunteers from PDGF-β (vein) healthy volunteers

Analysis of PDGF-β (vein) healthy volunteers was performed conditioning on Sex Healthy volunteers.

Table 13 – Analysis of PDGF-β (vein) healthy volunteers conditioning on Sex Healthy volunteers

Variable	Categories	PDGF-β (vein) healthy volunteers (pg/ml)			p
		M ± SD	95% CI	n	
Sex Healthy volunteers	Female	3592 ± 394	3289 – 3895	9	0.852
	Male	3624 ± 370	3389 – 3859	12	

When comparing of PDGF-β (vein) healthy volunteers depending on Sex Healthy volunteers there were no statistically significant differences (p = 0.852) *(applied method: Student's t-test)*.

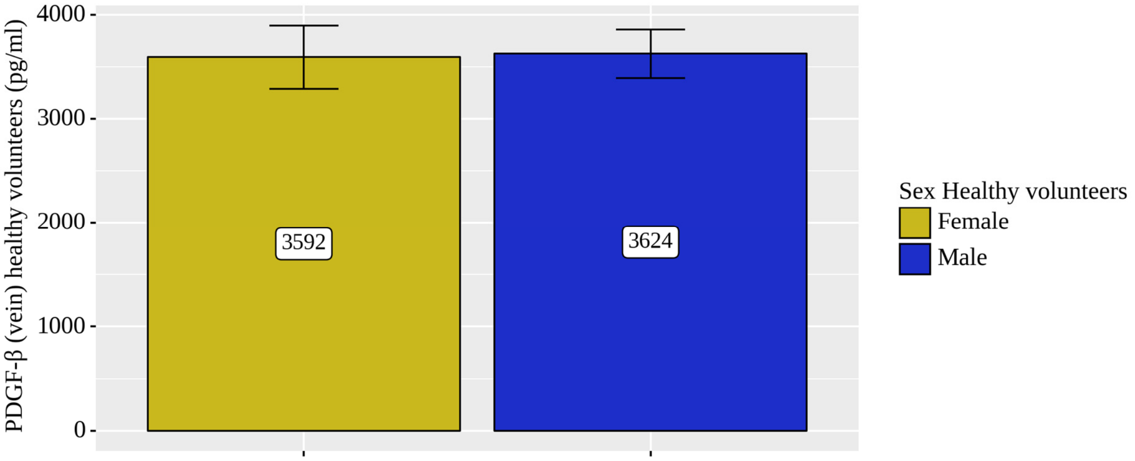

Figure 15 – Analysis of PDGF-β (vein) healthy volunteers conditioning on Sex Healthy volunteers

When evaluating the dependence of the probability of Male on the PDGF-β (vein) healthy volunteers using the ROC analysis, the following curve was obtained.

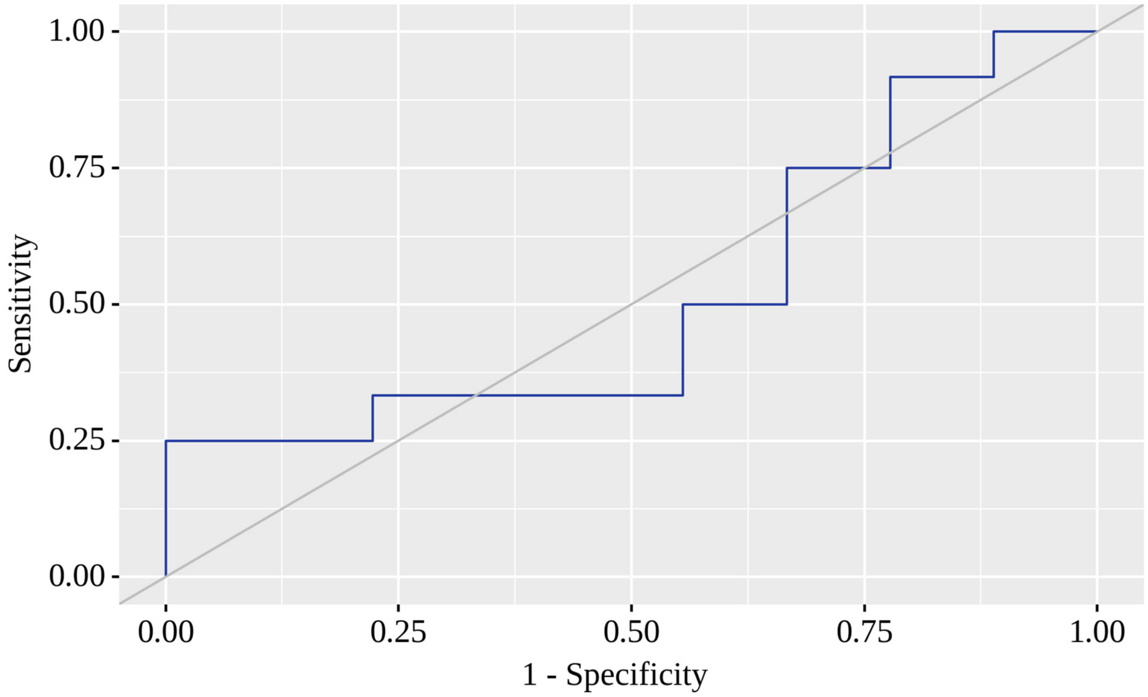

Figure 16 – ROC-curve characterizing the dependence of the probability Sex Healthy volunteers on PDGF-β (vein) healthy volunteers

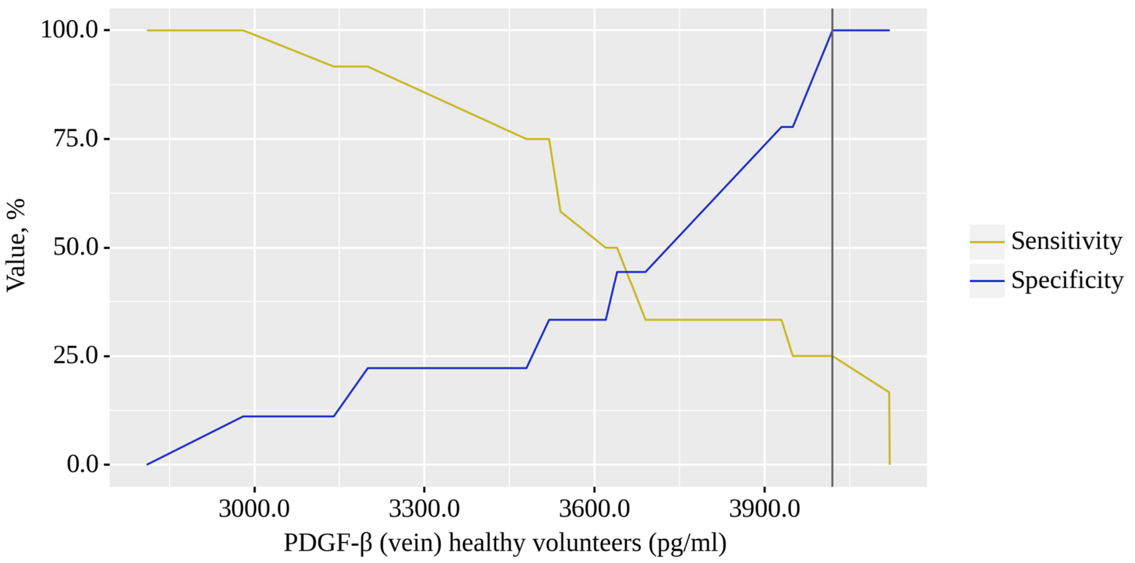

Figure 17 - Analysis of the sensitivity and specificity of Sex Healthy volunteers depending on PDGF-β (vein) healthy volunteers

The area under the ROC curve comprised 0.519 ± 0.130 with 95% CI: 0.264 - 0.773. The resulting model was not statistically significant (p = 0.887).

The cut-off value of PDGF-β (vein) healthy volunteers which corresponds to the highest Youden's J statistic is 4020.000 pg/ml. If PDGF-β (vein) healthy volunteers was greater than or equal to this value, Male was predicted. The sensitivity and specificity of the method were 25.0% and 100.0%, respectively.

We performed a correlation analysis of the association between TGF-β1 (vein) healthy volunteers and Age healthy volunteers.

Table 14 – Results of the correlation analysis of the association between TGF-β1 (vein) healthy volunteers and Age healthy volunteers

Variable	Correlation characteristics		
	r_{xy}	Strength of the association assesed using Chaddock scale	p
TGF-β1 (vein) healthy volunteers – Age healthy volunteers	0.028	None	0.904

There was no association between Age healthy volunteers and TGF-β1 (vein) healthy volunteers.

Observed dependence of Age healthy volunteers from TGF-β1 (vein) healthy volunteers is described by a linear regression equation:

$$Y_{\text{Age healthy volunteers}} = 0.001 \times X_{\text{TGF-}\beta\text{1 (vein) healthy volunteers}} + 40.052$$

With an 1 pg/ml increase of TGF-β1 (vein) healthy volunteers 0.001 change of Age healthy volunteers should be expected. According to the coefficient of determination R^2 of the resulting model, 0.1% of the observed variance of Age healthy volunteers were explained..

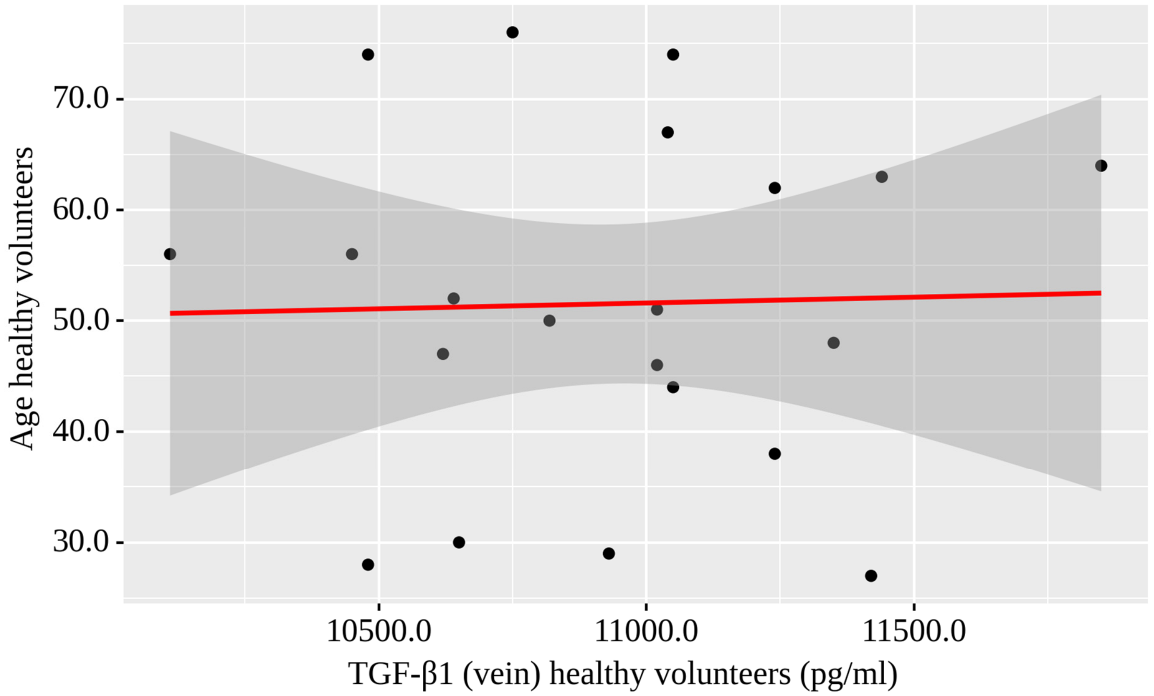

Figure 18 – Regression line characterizing the dependence of Age healthy volunteers from TGF-β1 (vein) healthy volunteers

We performed analysis of TGF-β1 (vein) healthy volunteers conditioning on Sex Healthy volunteers.

Table 15 – Analysis of TGF-β1 (vein) healthy volunteers conditioning on Sex Healthy volunteers

Variable	Categories	TGF-β1 (vein) healthy volunteers (pg/ml)			p
		M ± SD	95% CI	n	
Sex Healthy volunteers	Female	11095 ± 433	10733 – 11457	8	0.169
	Male	10838 ± 381	10607 – 11068	13	

When comparing of TGF-β1 (vein) healthy volunteers depending on Sex Healthy volunteers no statistically significant differences were revealed (p = 0.169) *(applied method: Student's t-test)*.

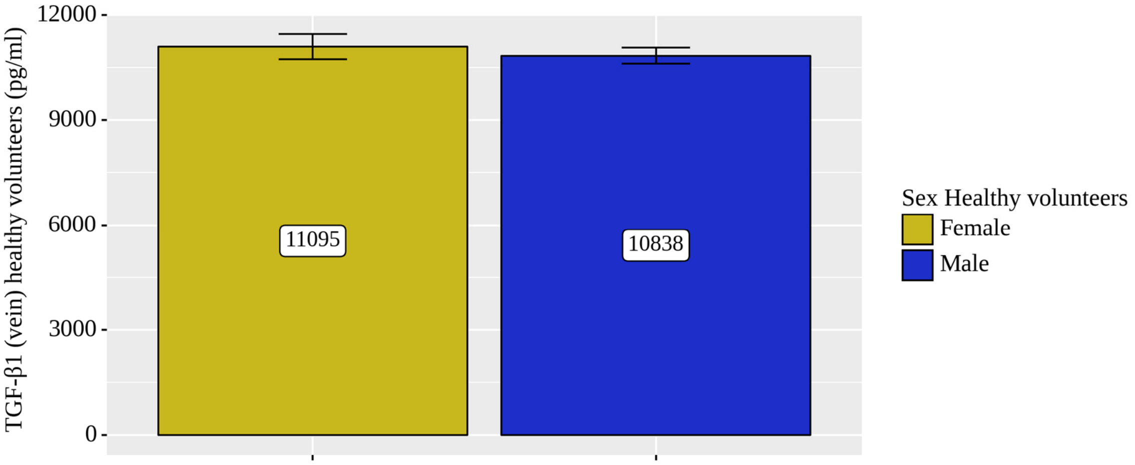

Figure 19 – Analysis of TGF-β1 (vein) healthy volunteers conditioning on Sex Healthy volunteers

When evaluating the dependence of the probability of Male on the TGF-β1 (vein) healthy volunteers using the ROC analysis, the following curve was obtained.

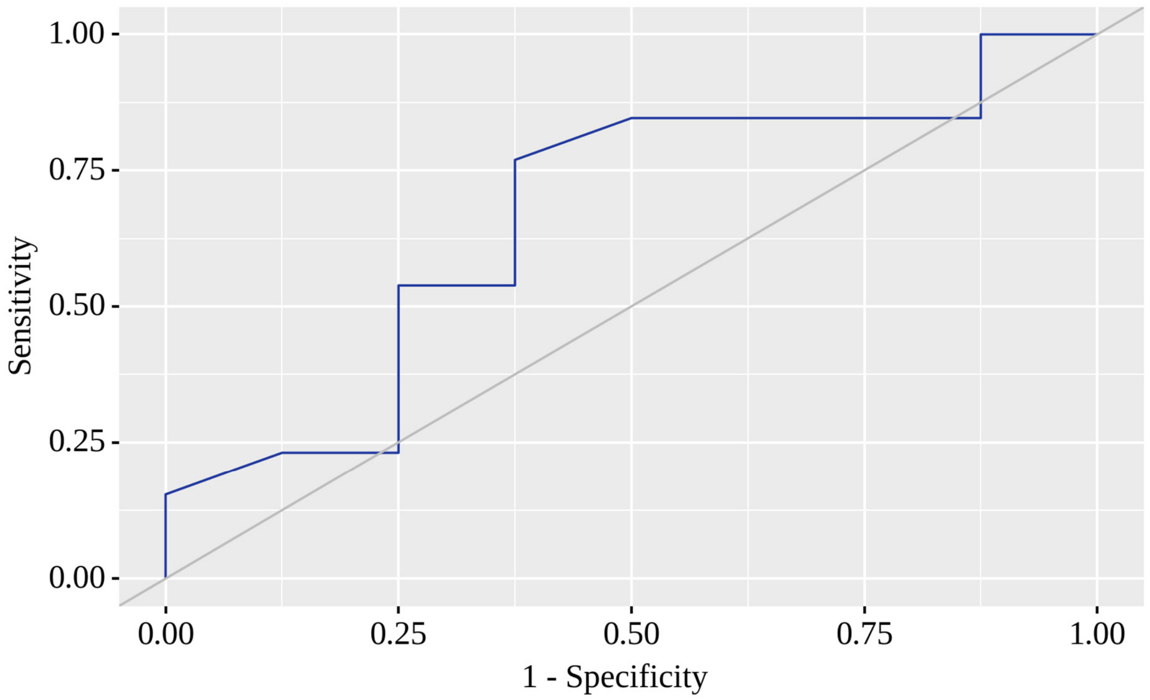

Figure 20 – ROC-curve characterizing the dependence of the probability Sex Healthy volunteers on TGF-β1 (vein) healthy volunteers

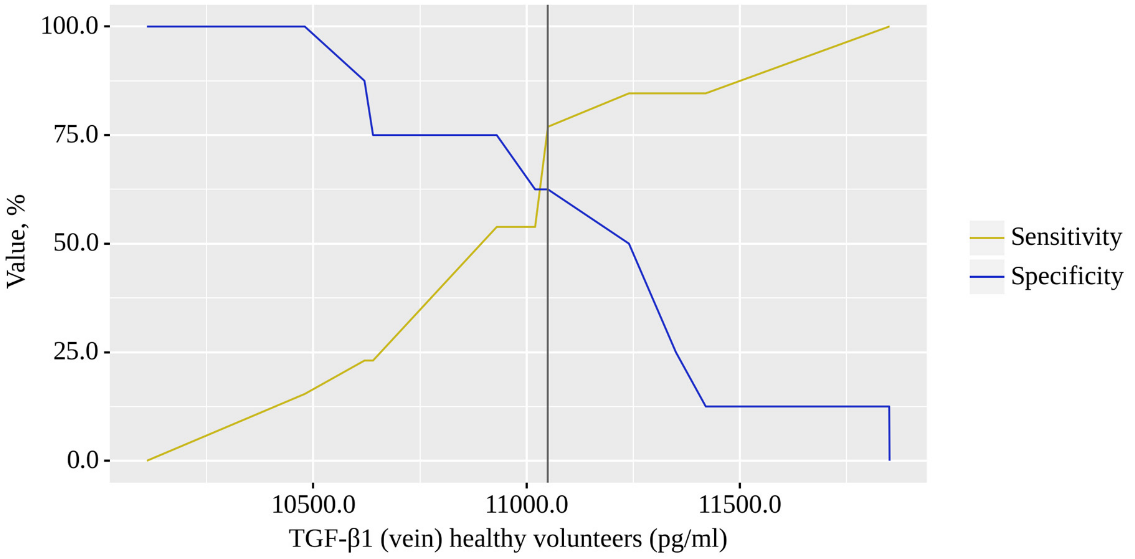

Figure 21 - Analysis of the sensitivity and specificity of Sex Healthy volunteers depending on TGF-β1 (vein) healthy volunteers

Table 16 – Threshold TGF-β1 (vein) healthy volunteers

Threshold	Sensitivity (Se), %	Specificity (Sp), %	PPV	NPV
11240	84.6	50.0	73.3	66.7
11050	76.9	62.5	76.9	62.5
**11040**	**69.2**	**62.5**	**75.0**	**55.6**
11020	53.8	62.5	70.0	45.5
10930	53.8	75.0	77.8	50.0

The area under the ROC curve comprised 0.663 ± 0.127 with 95% CI: 0.414 - 0.913. The resulting model was not statistically significant (p = 0.218).

The cut-off value of TGF-β1 (vein) healthy volunteers which corresponds to the highest Youden's J statistic is 11050.000 pg/ml. If TGF-β1 (vein) healthy volunteers was less than this value, Male was predicted. The sensitivity and specificity of the method were 76.9% and 62.5%, respectively.

Analysis of VEGF (vein) P/HV was performed conditioning on Patients/Healthy volunteers.

Table 17 – Analysis of VEGF (vein) P/HV conditioning on Patients/Healthy volunteers

Variable	Categories	VEGF (vein) P/HV (pg/ml)			p
		Me	$Q_1 – Q_3$	n	

Patients/Healthy volunteers	patient	39	17 – 153	8	0.004*
	healthy volunteer	279	250 – 295	33	

* – differences are statistically significant ($p < 0.05$)

In accordance with the presented table, when comparing of VEGF (vein) P/HV, statistically significant differences were revealed depending on Patients/Healthy volunteers ($p = 0.004$) *(applied method: Mann-Whitney U-test)*.

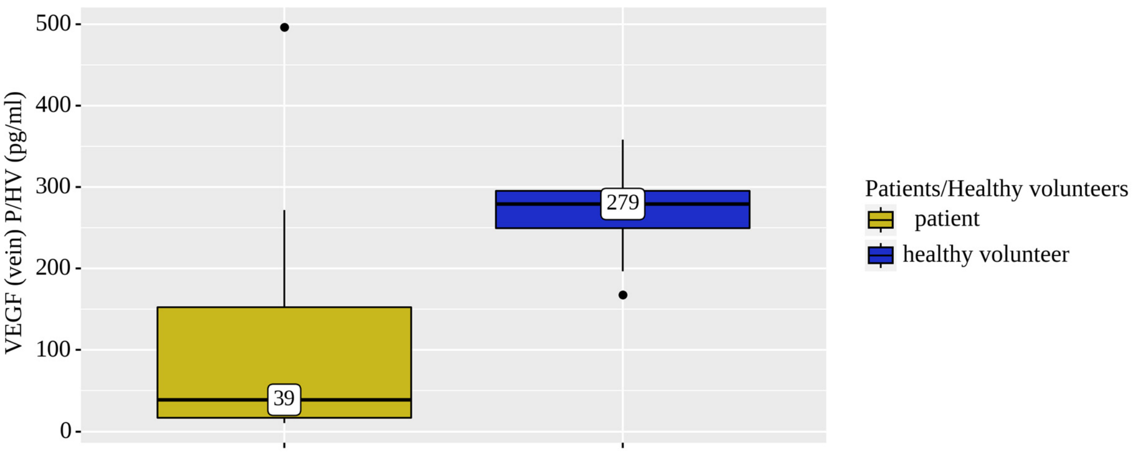

Figure 22 – Analysis of VEGF (vein) P/HV conditioning on Patients/Healthy volunteers

When evaluating the dependence of the probability of healthy volunteer on the VEGF (vein) P/HV using the ROC analysis, the following curve was obtained.

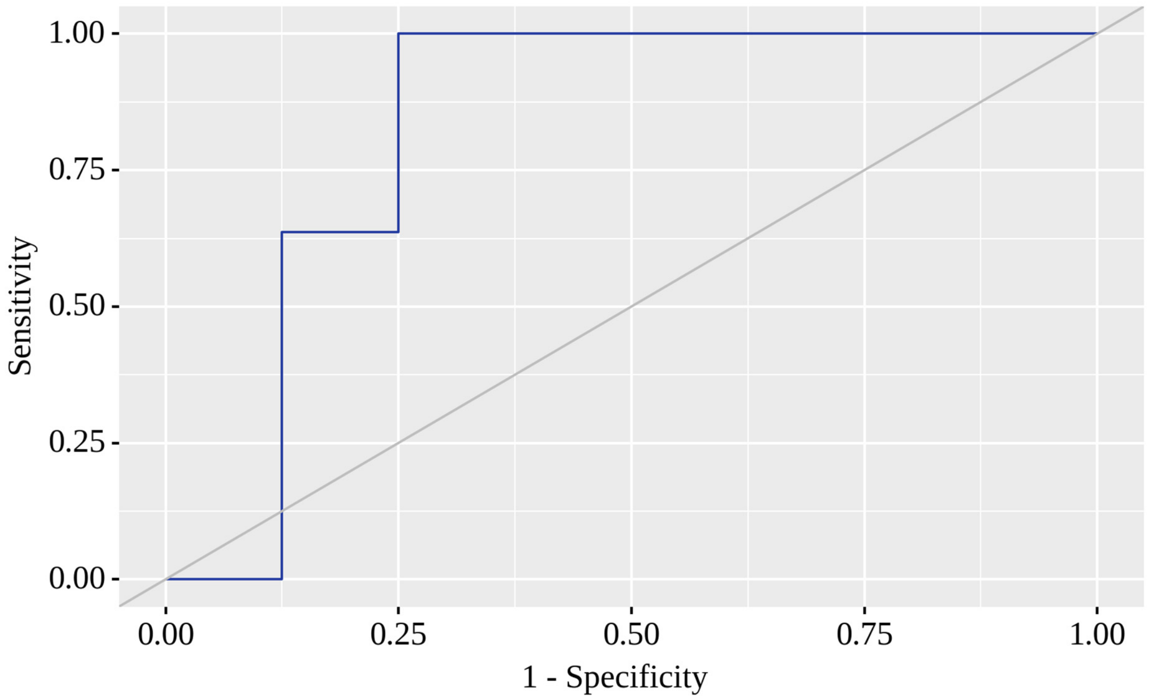

Figure 23 – ROC-curve characterizing the dependence of the probability Patients/Healthy volunteers on VEGF (vein) P/HV

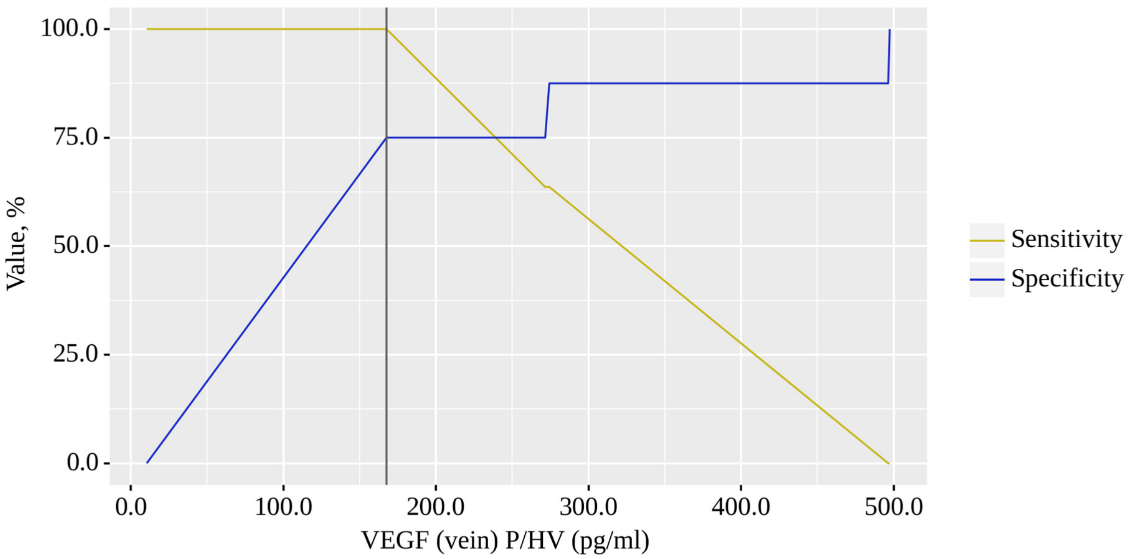

Figure 24 - Analysis of the sensitivity and specificity of Patients/Healthy volunteers depending on VEGF (vein) P/HV

Table 18 – Threshold VEGF (vein) P/HV

Threshold	Sensitivity (Se), %	Specificity (Sp), %	PPV	NPV

274	63.6	87.5	95.5	36.8
**272**	**63.6**	**75.0**	**91.3**	**33.3**
168	100.0	75.0	94.3	100.0

The area under the ROC curve comprised 0.830 ± 0.069 with 95% CI: 0.695 - 0.964. The resulting model was statistically significant (p = 0.004).

The cut-off value of VEGF (vein) P/HV which corresponds to the highest Youden's J statistic is 167.600 pg/ml. If VEGF (vein) P/HV was greater than or equal to this value, healthy volunteer was predicted. The sensitivity and specificity of the method were 100.0% and 75.0%, respectively.

Analysis of VEGF (vein) Patients was performed conditioning on Sides ChSDH.

Table 19 – Analysis of VEGF (vein) Patients conditioning on Sides ChSDH

Variable	Categories	VEGF (vein) Patients (pg/ml)			p
		Me	$Q_1 – Q_3$	n	
Sides ChSDH	Bilateral ChSDH	164	110 – 218	2	0.317
	Monolateral ChSDH	20	16 – 90	6	

When comparing of VEGF (vein) Patients depending on Sides ChSDH no statistically significant differences were revealed (p = 0.317) *(applied method: Mann-Whitney U-test)*.

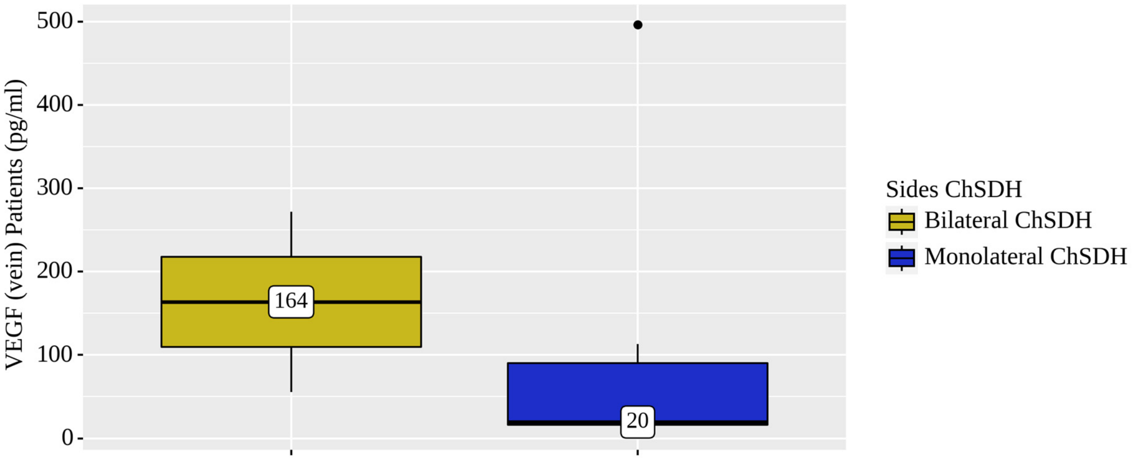

Figure 25 – Analysis of VEGF (vein) Patients conditioning on Sides ChSDH

When evaluating the dependence of the probability of Monolateral ChSDH on the VEGF (vein) Patients using the ROC analysis, the following curve was obtained.

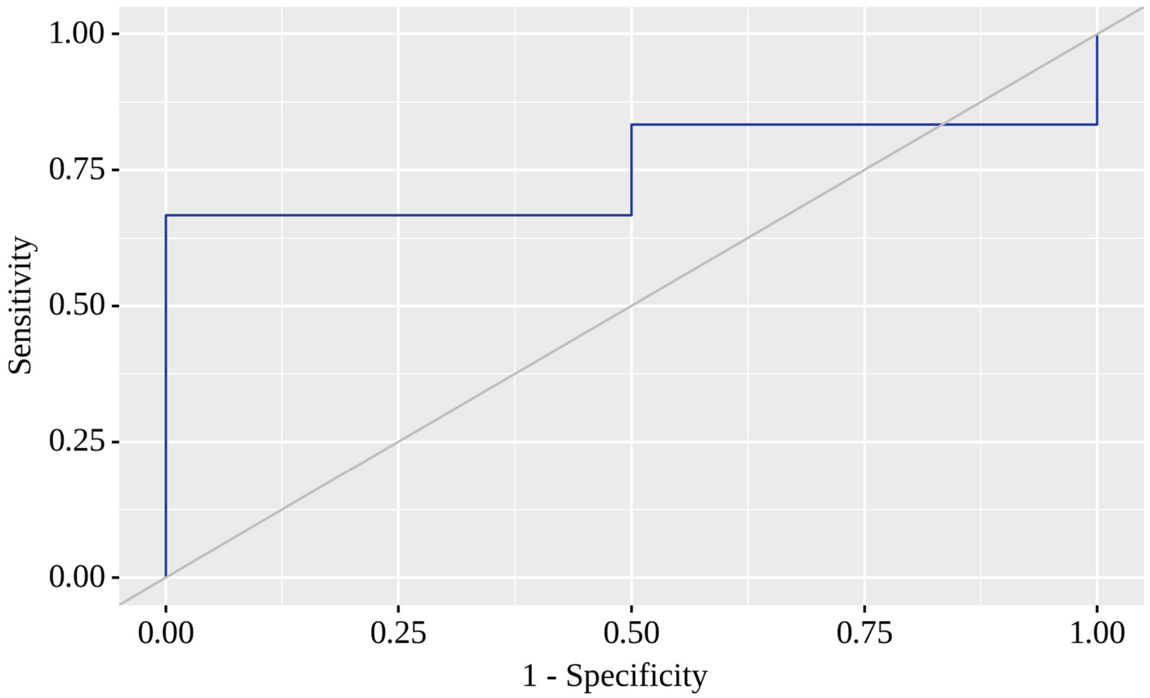

Figure 26 – ROC-curve characterizing the dependence of the probability Sides ChSDH on VEGF (vein) Patients

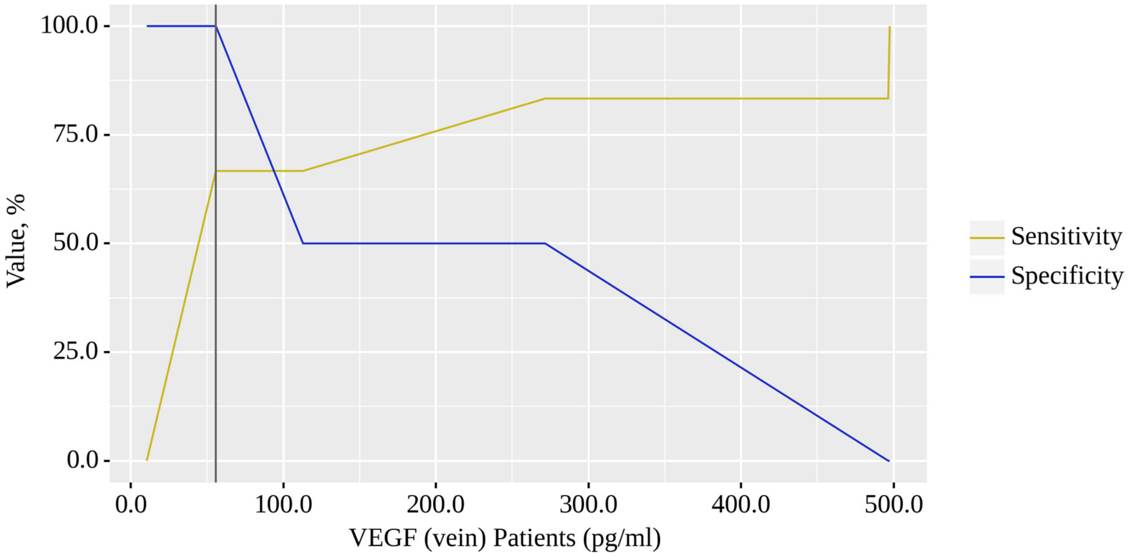

Figure 27 - Analysis of the sensitivity and specificity of Sides ChSDH depending on VEGF (vein) Patients

Table 20 – Threshold VEGF (vein) Patients

Threshold	Sensitivity (Se), %	Specificity (Sp), %	PPV	NPV
272	83.3	50.0	83.3	50.0
**113**	**66.7**	**50.0**	**80.0**	**33.3**
56	66.7	100.0	100.0	50.0

The area under the ROC curve comprised 0.750 ± 0.229 with 95% CI: 0.302 - 1.000. The resulting model was not statistically significant (p = 0.317).

The cut-off value of VEGF (vein) Patients which corresponds to the highest Youden's J statistic is 55.800 pg/ml. If VEGF (vein) Patients was less than this value, Monolateral ChSDH was predicted. The sensitivity and specificity of the method were 66.7% and 100.0%, respectively.

We performed analysis of VEGF (vein) Patients conditioning on Rebleeding on CT scans.

Table 21 – Analysis of VEGF (vein) Patients conditioning on Rebleeding on CT scans

Variable	Categories	VEGF (vein) Patients (pg/ml)			p
		Me	$Q_1 – Q_3$	n	
Rebleeding on CT scans	none	67	19 – 153	4	1.000
	rebleeded	36	17 – 166	4	

When comparing of VEGF (vein) Patients depending on Rebleeding on CT scans no statistically significant differences were revealed (p = 1.000) *(applied method: Mann-Whitney U-test)*.

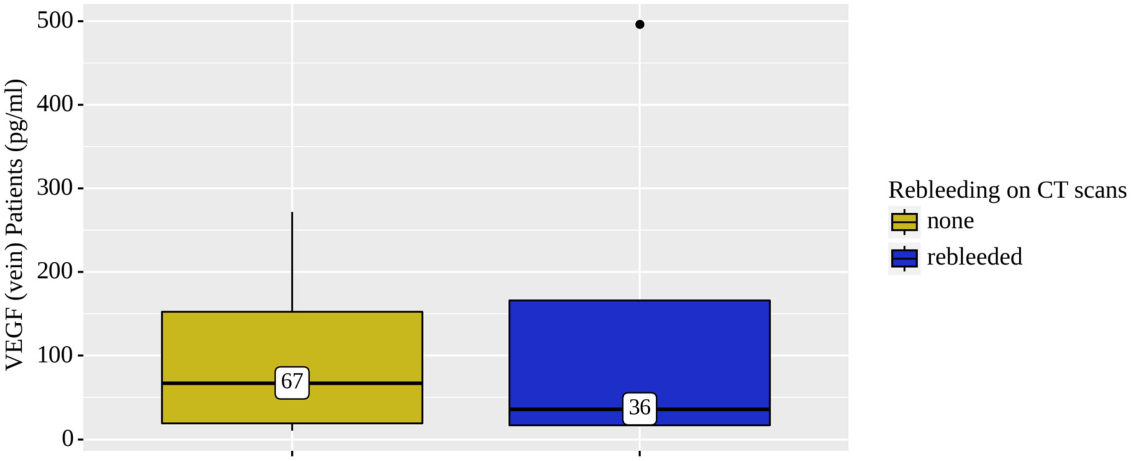

Figure 28 – Analysis of VEGF (vein) Patients conditioning on Rebleeding on CT scans

When evaluating the dependence of the probability of rebleeded on the VEGF (vein) Patients using the ROC analysis, the following curve was obtained.

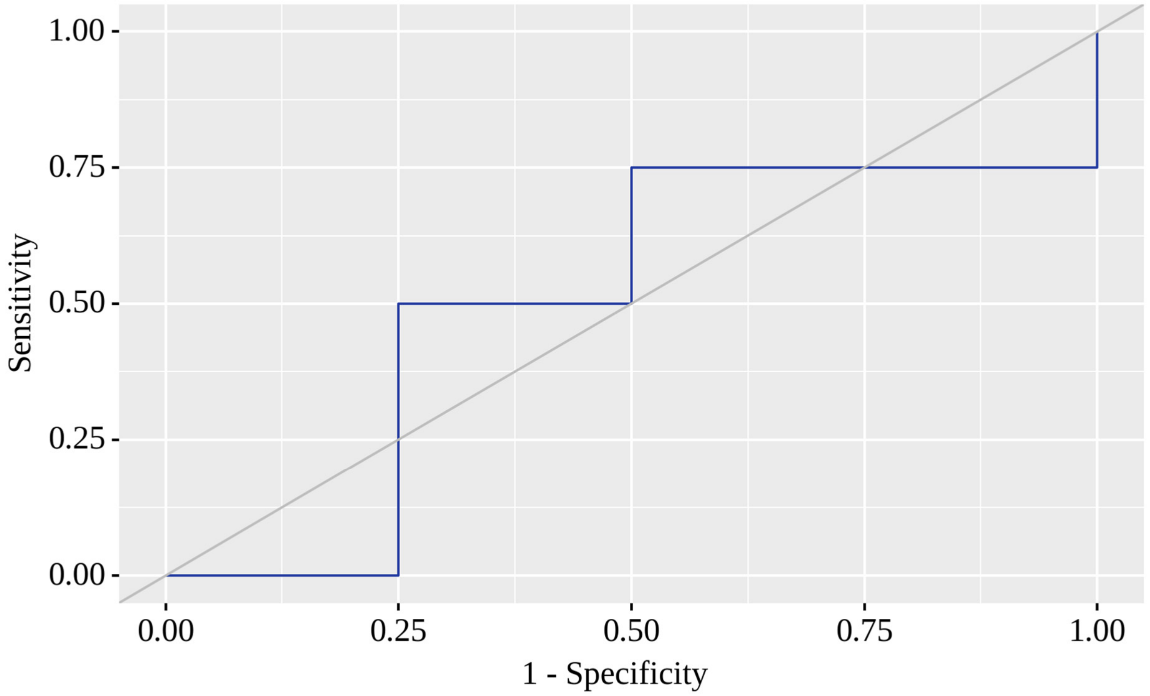

Figure 29 – ROC-curve characterizing the dependence of the probability Rebleeding on CT scans on VEGF (vein) Patients

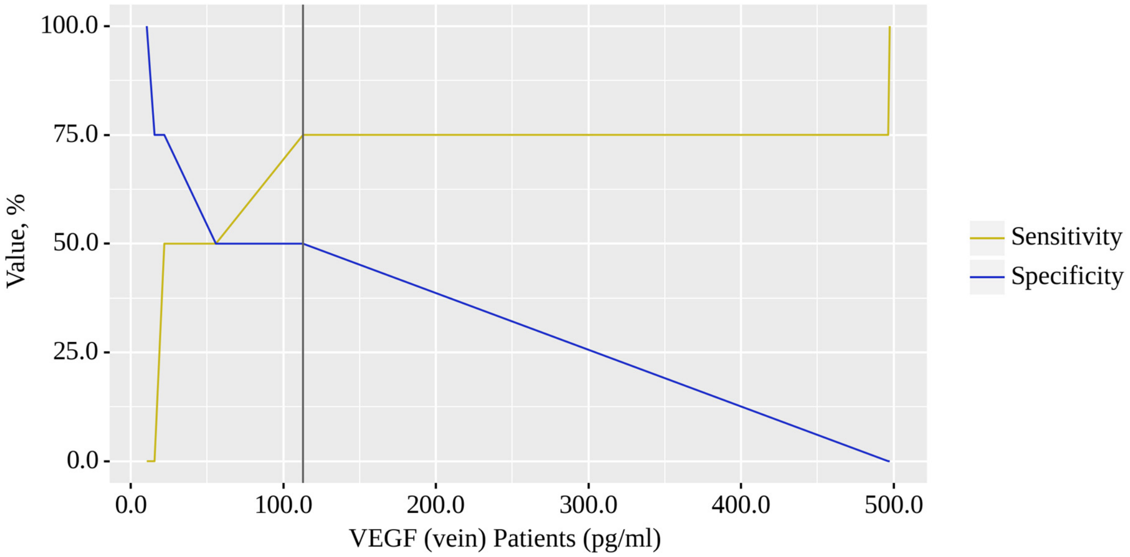

Figure 30 - Analysis of the sensitivity and specificity of Rebleeding on CT scans depending on VEGF (vein) Patients

Table 22 – Threshold VEGF (vein) Patients

Threshold	Sensitivity (Se), %	Specificity (Sp), %	PPV	NPV
113	75.0	50.0	60.0	66.7
**56**	**50.0**	**50.0**	**50.0**	**50.0**
22	50.0	75.0	66.7	60.0

The area under the ROC curve comprised 0.500 ± 0.217 with 95% CI: 0.076 - 0.924. The resulting model was not statistically significant (p = 1.000).

The cut-off value of VEGF (vein) Patients which corresponds to the highest Youden's J statistic is 112.900 pg/ml. If VEGF (vein) Patients was less than this value, rebleeded was predicted. The sensitivity and specificity of the method were 75.0% and 50.0%, respectively.

We performed a correlation analysis of the association between VEGF (vein) Patients and Total volume Pre Embol ChSDs.

Table 23 – Results of the correlation analysis of the association between VEGF (vein) Patients and Total volume Pre Embol ChSDs

Variable	Correlation characteristics		
	ρ	Strength of the association assesed using Chaddock scale	p
VEGF (vein) Patients – Total volume Pre Embol ChSDs	0.731	Strong	0.040*

* – differences are statistically significant (p < 0.05)

A strong correlation positive association between Total volume Pre Embol ChSDs and VEGF (vein) Patients was estimated.

Observed dependence of Total volume Pre Embol ChSDs from VEGF (vein) Patients is described by a linear regression equation:

$$Y_{\text{Total volume Pre Embol ChSDs}} = 0.156 \times X_{\text{VEGF (vein) Patients}} + 82.944$$

With an 1 pg/ml increase of VEGF (vein) Patients 0.156 ml change of Total volume Pre Embol ChSDs should be expected. According to the coefficient of determination R^2 of the resulting model, 16.8% of the observed variance of Total volume Pre Embol ChSDs were explained..

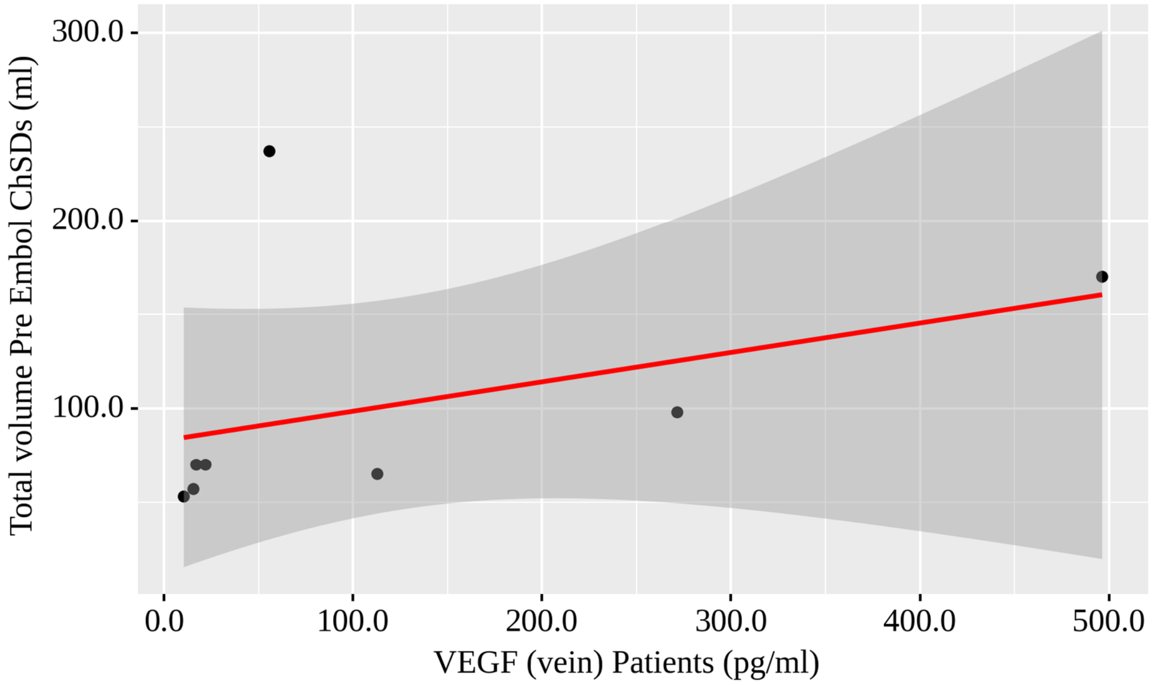

Figure 31 – Regression line characterizing the dependence of Total volume Pre Embol ChSDs from VEGF (vein) Patients

We performed analysis of VEGF (vein) Patients conditioning on Surgery.

Table 24 – Analysis of VEGF (vein) Patients conditioning on Surgery

Variable	Categories	VEGF (vein) Patients (pg/ml)			p
		M ± SD	95% CI	n	
Surgery	none	199 ± 233	-172 – 569	4	0.299
	operated	52 ± 44	-18 – 122	4	

When comparing of VEGF (vein) Patients depending on Surgery no statistically significant differences were revealed (p = 0.299) *(applied method: Welch's t-test)*.

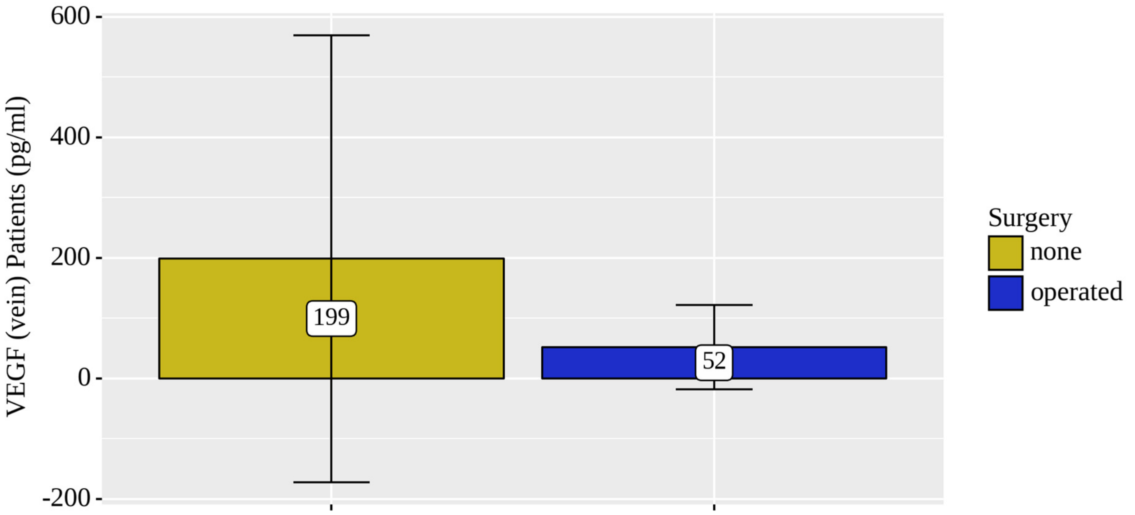

Figure 32 – Analysis of VEGF (vein) Patients conditioning on Surgery

When evaluating the dependence of the probability of operated on the VEGF (vein) Patients using the ROC analysis, the following curve was obtained.

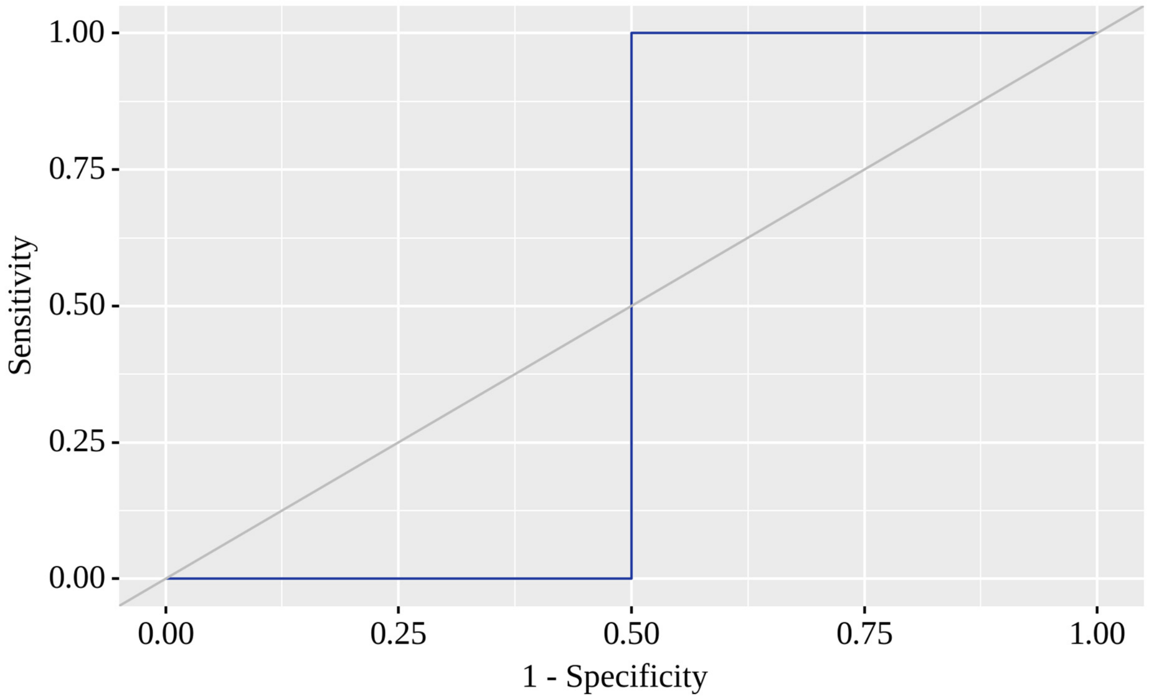

Figure 33 – ROC-curve characterizing the dependence of the probability Surgery on VEGF (vein) Patients

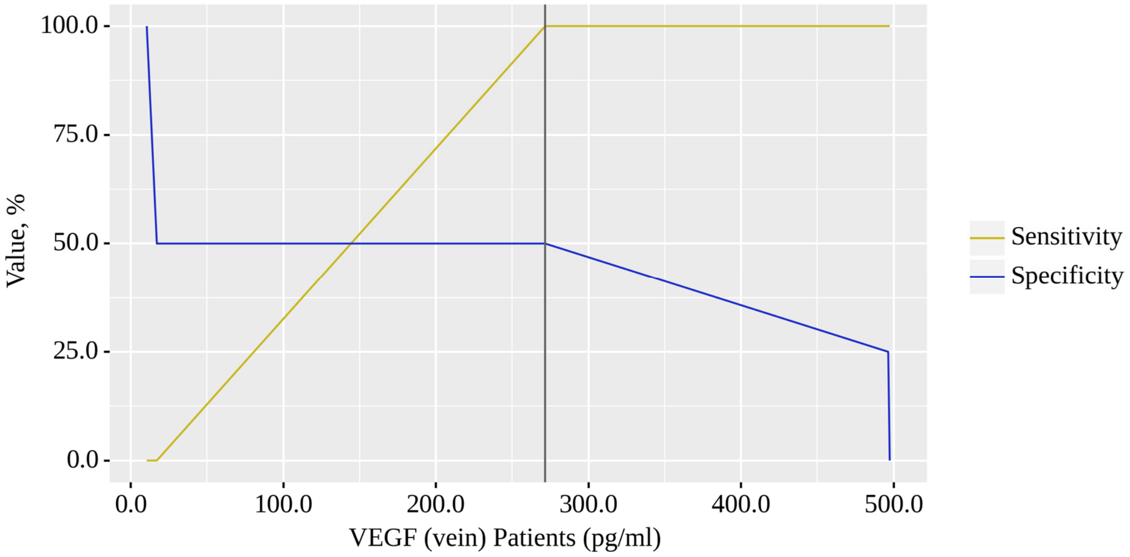

Figure 34 - Analysis of the sensitivity and specificity of Surgery depending on VEGF (vein) Patients

Table 25 – Threshold VEGF (vein) Patients

Threshold	Sensitivity (Se), %	Specificity (Sp), %	PPV	NPV
**272**	**100.0**	**50.0**	**66.7**	**100.0**

The area under the ROC curve comprised 0.500 ± 0.217 with 95% CI: 0.076 - 0.924. The resulting model was not statistically significant (p = 1.000).

The cut-off value of VEGF (vein) Patients which corresponds to the highest Youden's J statistic is 271.600 pg/ml. If VEGF (vein) Patients was less than this value, operated was predicted. The sensitivity and specificity of the method were 100.0% and 50.0%, respectively.

We performed a correlation analysis of the association between VEGF (artery) Patients and VEGF (vein) Patients.

Table 26 – Results of the correlation analysis of the association between VEGF (artery) Patients and VEGF (vein) Patients

Variable	Correlation characteristics		
	ρ	Strength of the association assesed using Chaddock scale	p
VEGF (artery) Patients – VEGF (vein) Patients	0.536	Close	0.215

A close correlation positive association between VEGF (vein) Patients and VEGF (artery) Patients was estimated.

Observed dependence of VEGF (vein) Patients from VEGF (artery) Patients is described by a linear regression equation:

$$Y_{\text{VEGF (vein) Patients}} = 0.106 \times X_{\text{VEGF (artery) Patients}} + 94.169$$

With an 1 pg/ml increase of VEGF (artery) Patients 0.106 pg/ml change of VEGF (vein) Patients should be expected. According to the coefficient of determination R^2 of the resulting model, 0.4% of the observed variance of VEGF (vein) Patients were explained..

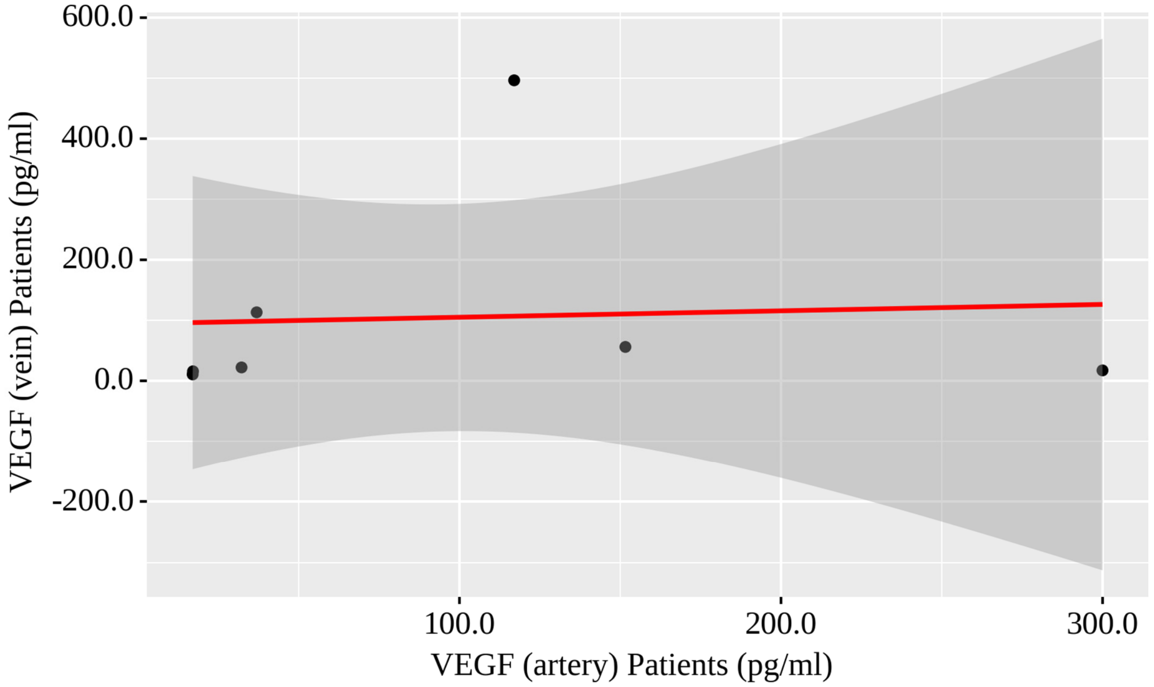

Figure 35 – Regression line characterizing the dependence of VEGF (vein) Patients from VEGF (artery) Patients

We performed analysis of VEGF (artery) Patients conditioning on Sides ChSDH.

Table 27 – Analysis of VEGF (artery) Patients conditioning on Sides ChSDH

Variable	Categories	VEGF (artery) Patients (pg/ml)			p
		Me	$Q_1 – Q_3$	n	
Sides ChSDH	Bilateral ChSDH	152	152 – 152	1	0.317

	Monolateral ChSDH	35	21 – 97	6	

When comparing of VEGF (artery) Patients depending on Sides ChSDH there were no statistically significant differences (p = 0.317) *(applied method: Mann-Whitney U-test)*.

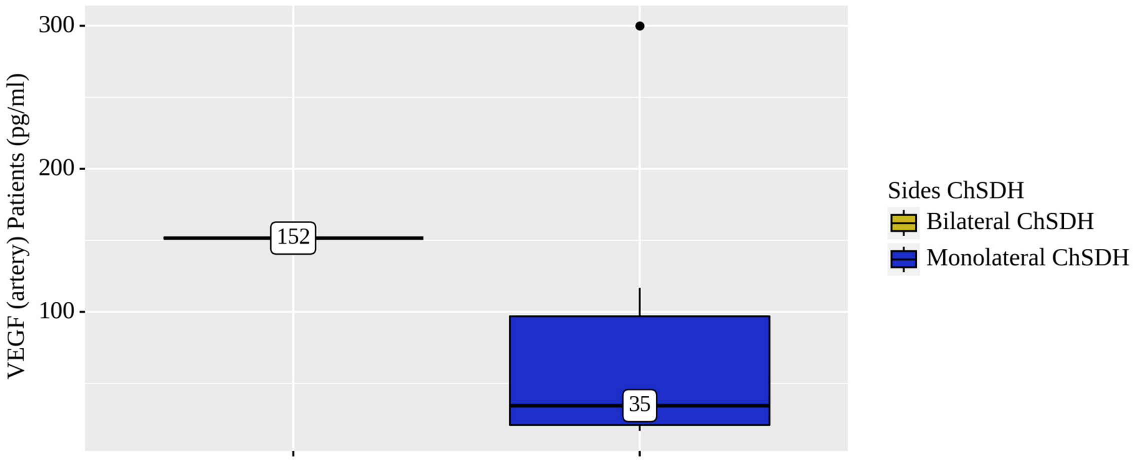

Figure 36 – Analysis of VEGF (artery) Patients conditioning on Sides ChSDH

When evaluating the dependence of the probability of Monolateral ChSDH on the VEGF (artery) Patients using the ROC analysis, the following curve was obtained.

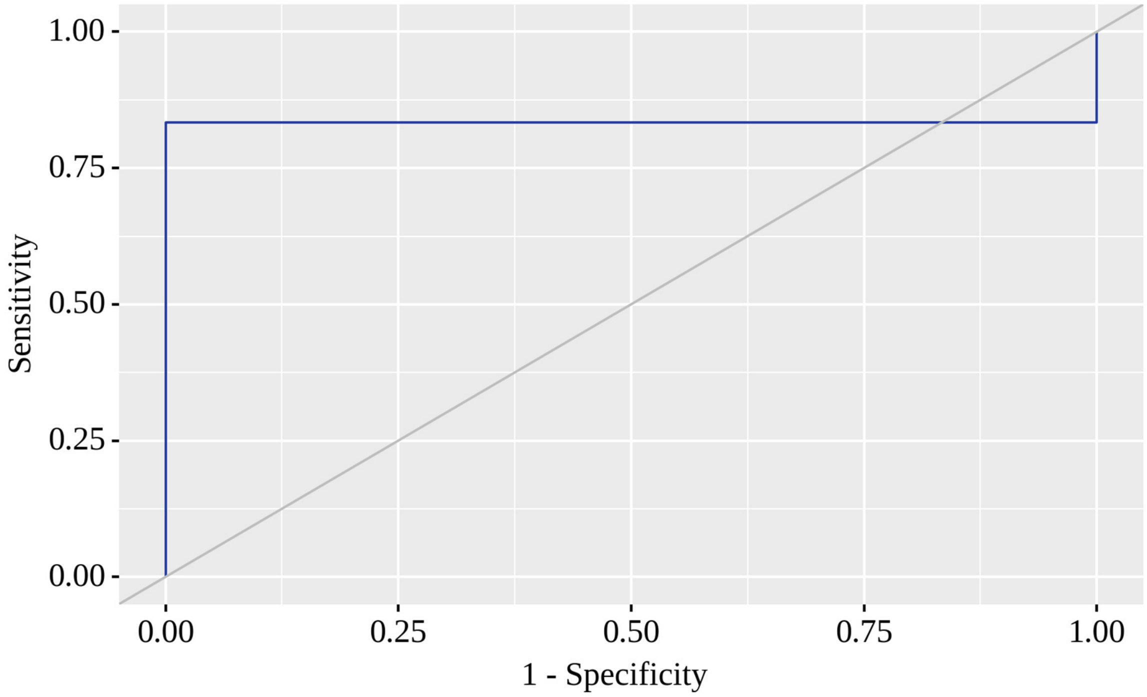

Figure 37 – ROC-curve characterizing the dependence of the probability Sides ChSDH on VEGF (artery) Patients

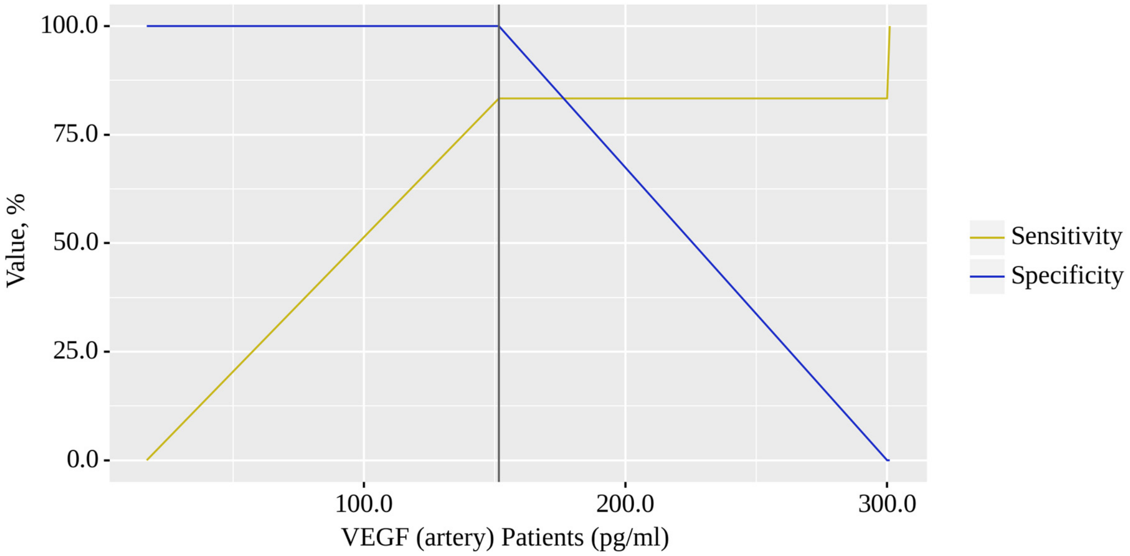

Figure 38 - Analysis of the sensitivity and specificity of Sides ChSDH depending on VEGF (artery) Patients

Table 28 – Threshold VEGF (artery) Patients

Threshold	Sensitivity (Se), %	Specificity (Sp), %	PPV	NPV
**152**	**83.3**	**100.0**	**100.0**	**50.0**

The area under the ROC curve comprised 0.833 ± 0.275 with 95% CI: 0.294 - 1.000. The resulting model was not statistically significant ($p = 0.317$).

The cut-off value of VEGF (artery) Patients which corresponds to the highest Youden's J statistic is 151.600 pg/ml. If VEGF (artery) Patients was less than this value, Monolateral ChSDH was predicted. The sensitivity and specificity of the method were 83.3% and 100.0%, respectively.

Correlation analysis of the association between VEGF (artery) Patients and Total volume Pre Embol ChSDs was performed.

Table 29 – Results of the correlation analysis of the association between VEGF (artery) Patients and Total volume Pre Embol ChSDs

Variable	Correlation characteristics		
	ρ	Strength of the association assesed using Chaddock scale	p
VEGF (artery) Patients – Total volume Pre Embol ChSDs	0.793	Strong	0.033*

* – differences are statistically significant ($p < 0.05$)

A strong correlation positive association between Total volume Pre Embol ChSDs and VEGF (artery) Patients was estimated.

Observed dependence of Total volume Pre Embol ChSDs from VEGF (artery) Patients is described by a linear regression equation:

$$Y_{\text{Total volume Pre Embol ChSDs}} = 0.216 \times X_{\text{VEGF (artery) Patients}} + 82.446$$

With an 1 pg/ml increase of VEGF (artery) Patients 0.216 ml change of Total volume Pre Embol ChSDs should be expected. According to the coefficient of determination R^2 of the resulting model, 9.9% of the observed variance of Total volume Pre Embol ChSDs were explained..

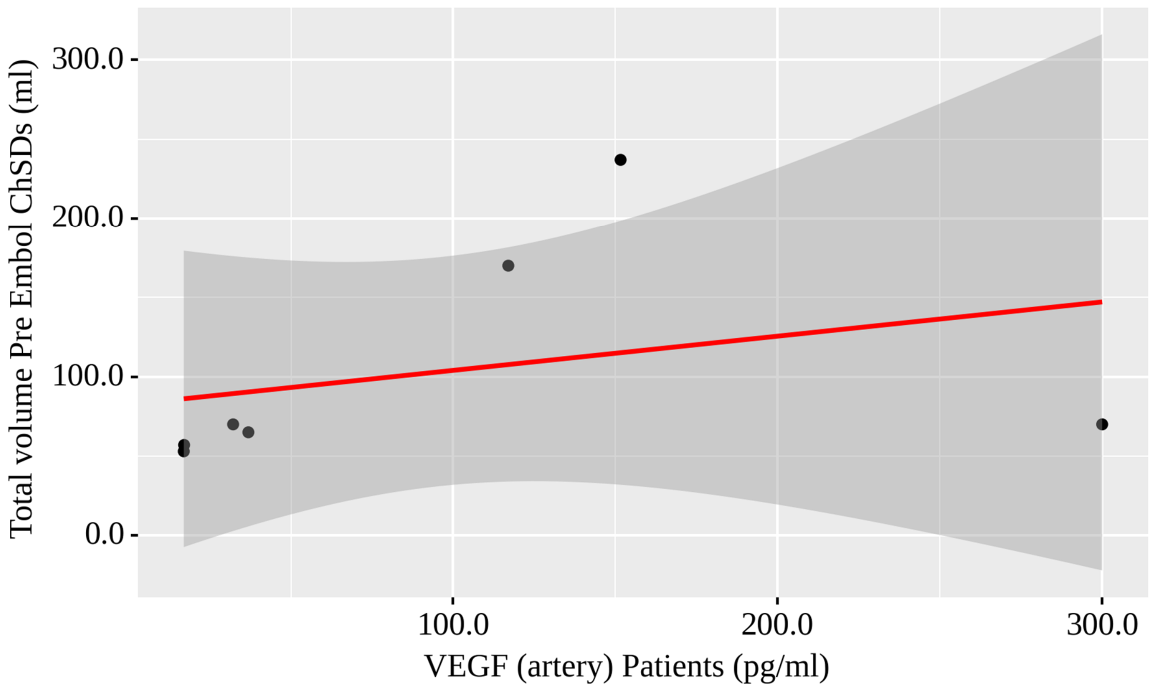

Figure 39 – Regression line characterizing the dependence of Total volume Pre Embol ChSDs from VEGF (artery) Patients

Analysis of VEGF (artery) Patients was performed conditioning on Rebleeding on CT scans.

Table 30 – Analysis of VEGF (artery) Patients conditioning on Rebleeding on CT scans

Variable	Categories	VEGF (artery) Patients (pg/ml)			p
		M ± SD	95% CI	n	
Rebleeding on CT scans	none	29 ± 10	3 – 55	3	0.151
	rebleeded	146 ± 117	-40 – 333	4	

When comparing of VEGF (artery) Patients depending on Rebleeding on CT scans there were no statistically significant differences (p = 0.151) *(applied method: Student's t-test)*.

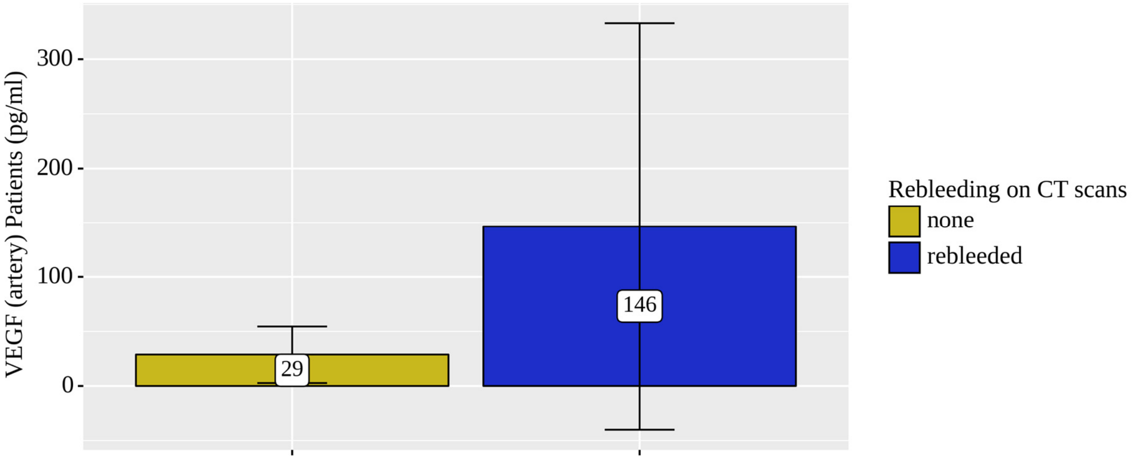

Figure 40 – Analysis of VEGF (artery) Patients conditioning on Rebleeding on CT scans

When evaluating the dependence of the probability of rebleeded on the VEGF (artery) Patients using the ROC analysis, the following curve was obtained.

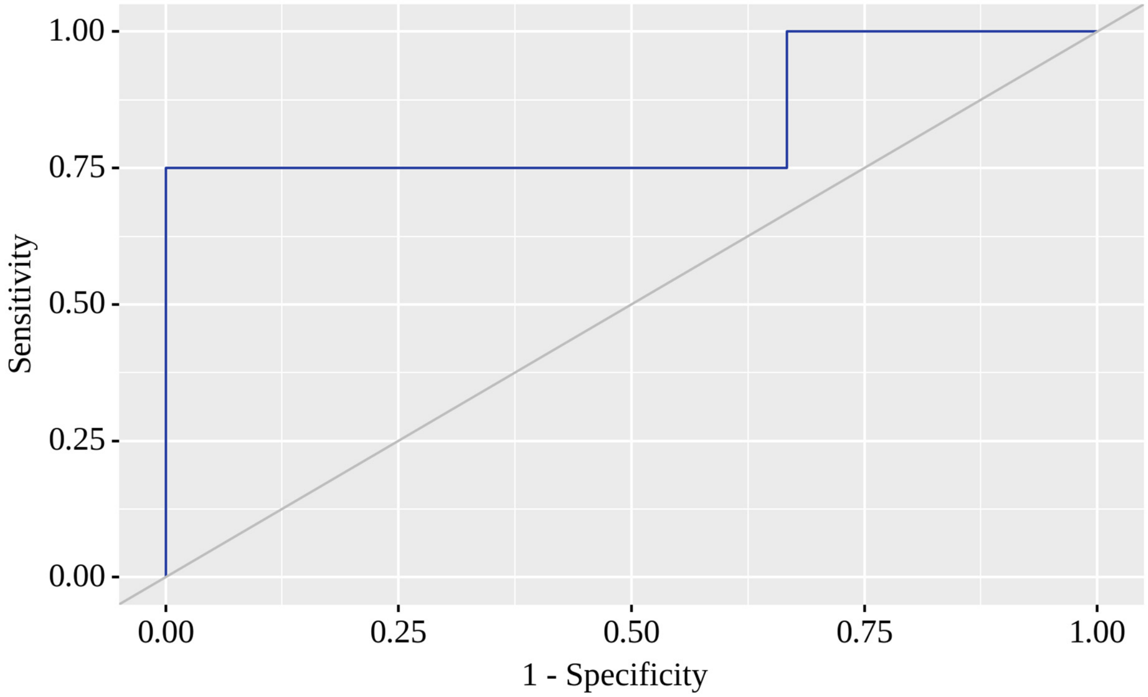

Figure 41 – ROC-curve characterizing the dependence of the probability Rebleeding on CT scans on VEGF (artery) Patients

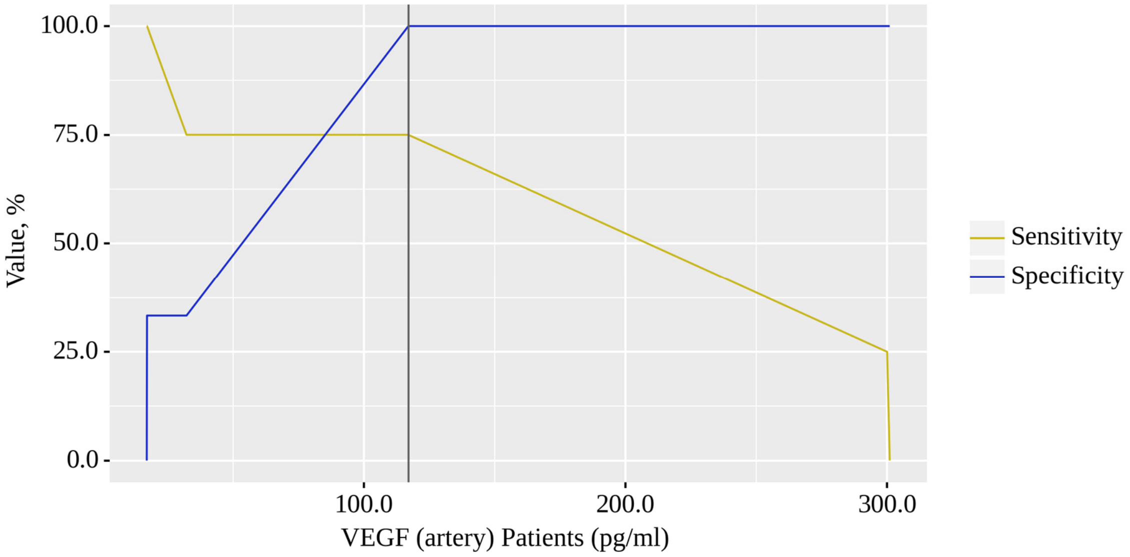

Figure 42 - Analysis of the sensitivity and specificity of Rebleeding on CT scans depending on VEGF (artery) Patients

Table 31 – Threshold VEGF (artery) Patients

Threshold	Sensitivity (Se), %	Specificity (Sp), %	PPV	NPV
**117**	**75.0**	**100.0**	**100.0**	**75.0**

The area under the ROC curve comprised 0.833 ± 0.164 with 95% CI: 0.511 - 1.000. The resulting model was not statistically significant (p = 0.157).

The cut-off value of VEGF (artery) Patients which corresponds to the highest Youden's J statistic is 117.000 pg/ml. If VEGF (artery) Patients was greater than or equal to this value, rebleeded was predicted. The sensitivity and specificity of the method were 75.0% and 100.0%, respectively.

Analysis of VEGF (artery) Patients was performed conditioning on Surgery.

Table 32 – Analysis of VEGF (artery) Patients conditioning on Surgery

Variable	Categories	VEGF (artery) Patients (pg/ml)			p
		Me	$Q_1 - Q_3$	n	
Surgery	none	17	17 – 67	3	0.157
	operated	94	36 – 189	4	

When comparing of VEGF (artery) Patients depending on Surgery there were no statistically significant differences (p = 0.157) *(applied method: Mann-Whitney U-test)*.

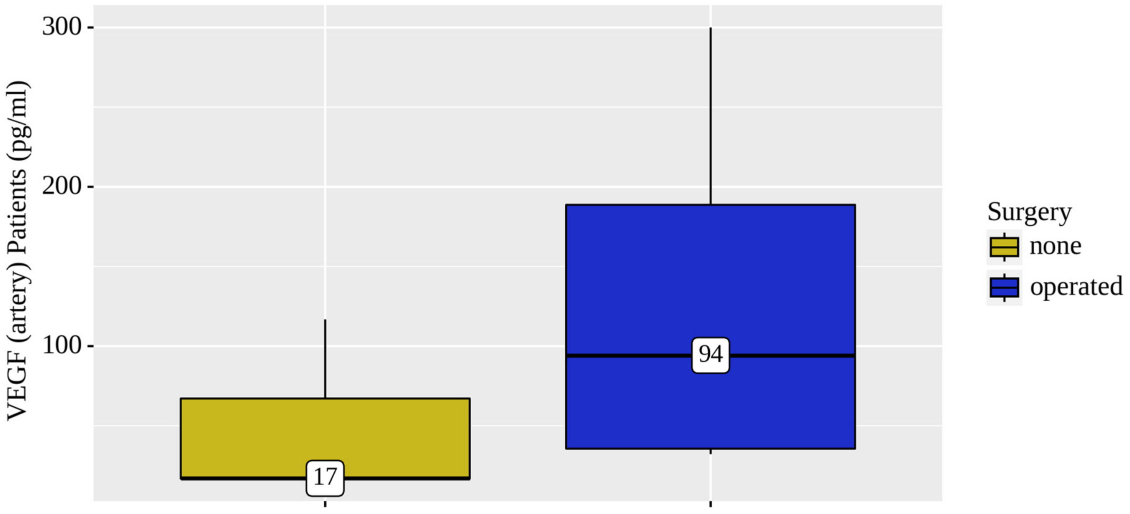

Figure 43 – Analysis of VEGF (artery) Patients conditioning on Surgery

When evaluating the dependence of the probability of operated on the VEGF (artery) Patients using the ROC analysis, the following curve was obtained.

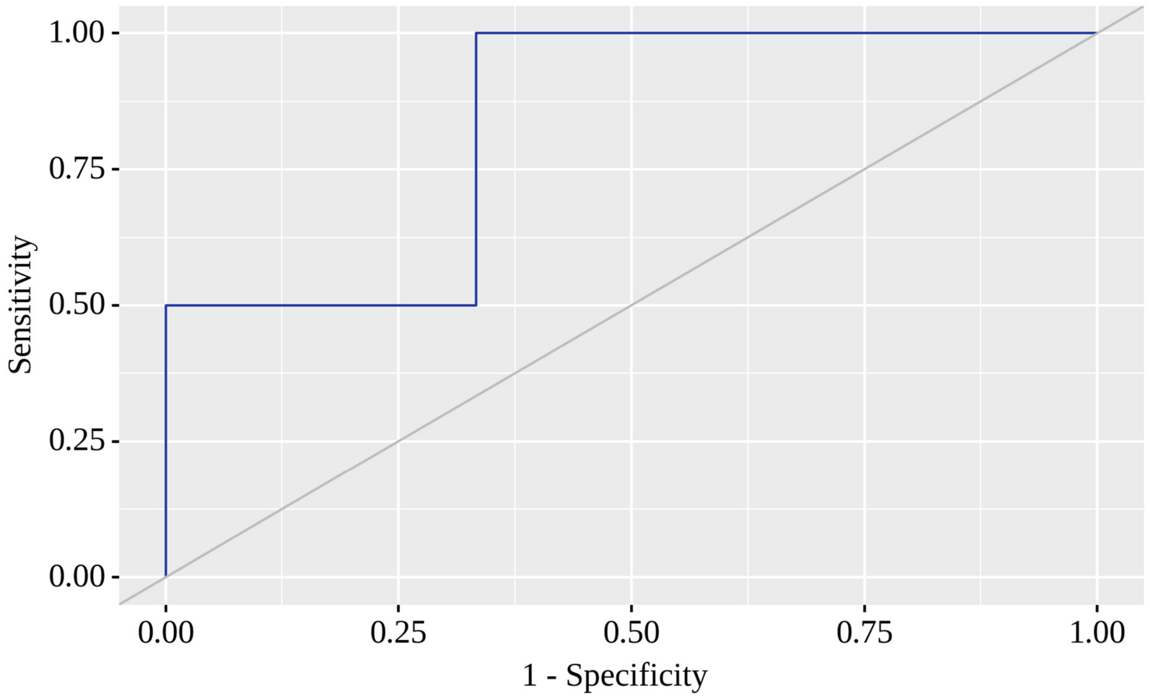

Figure 44 – ROC-curve characterizing the dependence of the probability Surgery on VEGF (artery) Patients

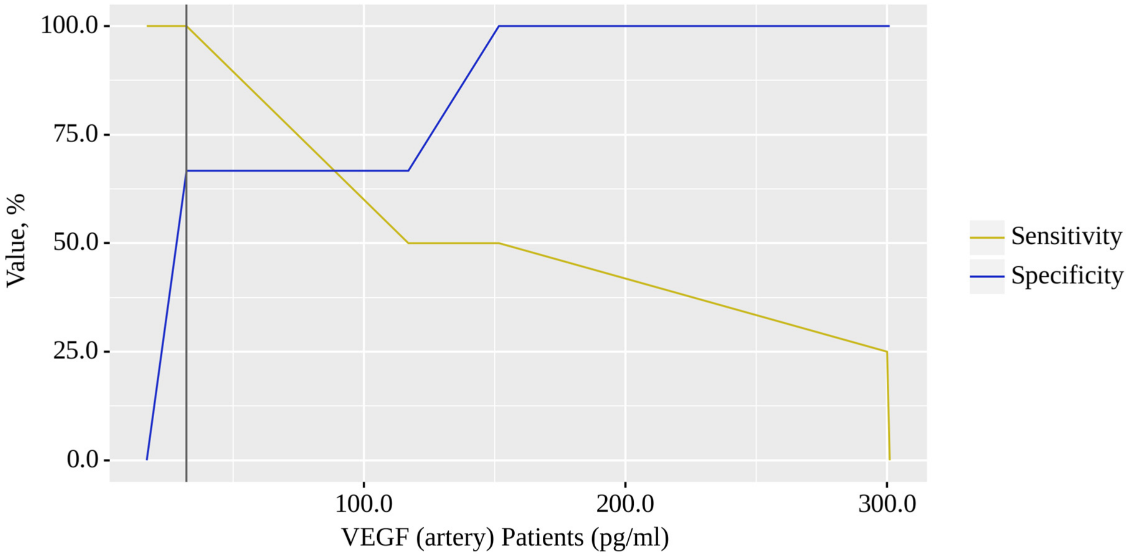

Figure 45 - Analysis of the sensitivity and specificity of Surgery depending on VEGF (artery) Patients

Table 33 – Threshold VEGF (artery) Patients

Threshold	Sensitivity (Se), %	Specificity (Sp), %	PPV	NPV
152	50.0	100.0	100.0	60.0
**117**	**50.0**	**66.7**	**66.7**	**50.0**
32	100.0	66.7	80.0	100.0

The area under the ROC curve comprised 0.833 ± 0.164 with 95% CI: 0.511 - 1.000. The resulting model was not statistically significant (p = 0.157).

The cut-off value of VEGF (artery) Patients which corresponds to the highest Youden's J statistic is 32.200 pg/ml. If VEGF (artery) Patients was greater than or equal to this value, operated was predicted. The sensitivity and specificity of the method were 100.0% and 66.7%, respectively.

We performed analysis of MMP-9 (vein) P/HV conditioning on Patients/Healthy volunteers.

Table 34 – Analysis of MMP-9 (vein) P/HV conditioning on Patients/Healthy volunteers

Variable	Categories	MMP-9 (vein) P/HV (ng/ml)			p
		Me	$Q_1 – Q_3$	n	
Patients/Healthy volunteers	patient	692	288 – 844	8	0.278
	healthy volunteer	453	379 – 486	33	

When comparing of MMP-9 (vein) P/HV depending on Patients/Healthy volunteers there were no statistically significant differences (p = 0.278) *(applied method: Mann-Whitney U-test)*.

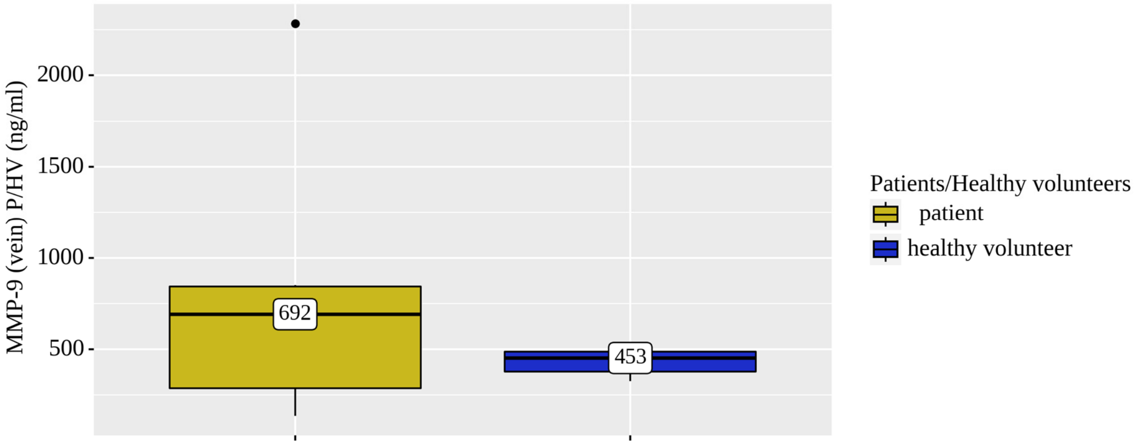

Figure 46 – Analysis of MMP-9 (vein) P/HV conditioning on Patients/Healthy volunteers

When evaluating the dependence of the probability of healthy volunteer on the MMP-9 (vein) P/HV using the ROC analysis, the following curve was obtained.

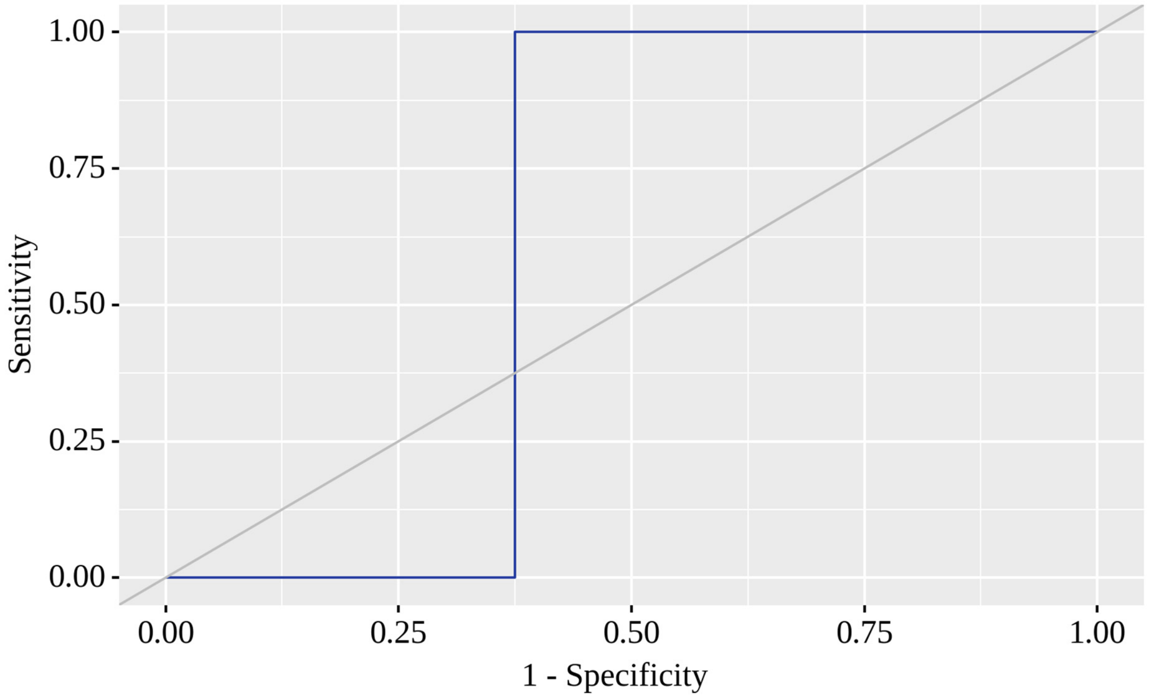

Figure 47 – ROC-curve characterizing the dependence of the probability Patients/Healthy volunteers on MMP-9 (vein) P/HV

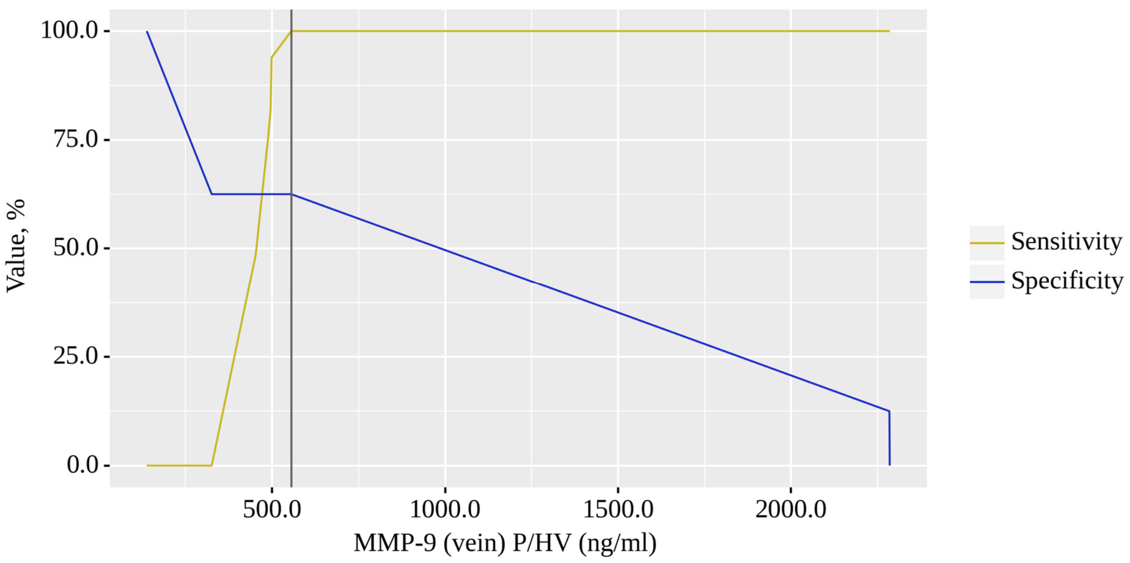

Figure 48 - Analysis of the sensitivity and specificity of Patients/Healthy volunteers depending on MMP-9 (vein) P/HV

Table 35 – Threshold MMP-9 (vein) P/HV

Threshold	Sensitivity (Se), %	Specificity (Sp), %	PPV	NPV
556	100.0	62.5	91.7	100.0
499	93.9	62.5	91.2	71.4
496	81.8	62.5	90.0	45.5
489	75.8	62.5	89.3	38.5
**461**	**54.5**	**62.5**	**85.7**	**25.0**

The area under the ROC curve comprised 0.625 ± 0.116 with 95% CI: 0.397 - 0.853. The resulting model was not statistically significant (p = 0.278).

The cut-off value of MMP-9 (vein) P/HV which corresponds to the highest Youden's J statistic is 555.600 ng/ml. If MMP-9 (vein) P/HV was less than this value, healthy volunteer was predicted. The sensitivity and specificity of the method were 100.0% and 62.5%, respectively.

We performed analysis of MMP-9 (vein) Patients conditioning on Sides ChSDH.

Table 36 – Analysis of MMP-9 (vein) Patients conditioning on Sides ChSDH

Variable	Categories	MMP-9 (vein) Patients (ng/ml)			p
		Me	$Q_1 – Q_3$	n	
Sides ChSDH	Bilateral ChSDH	703	629 – 776	2	0.505
	Monolateral ChSDH	560	281 – 838	6	

When comparing of MMP-9 (vein) Patients depending on Sides ChSDH there were no statistically significant differences ($p = 0.505$) *(applied method: Mann-Whitney U-test)*.

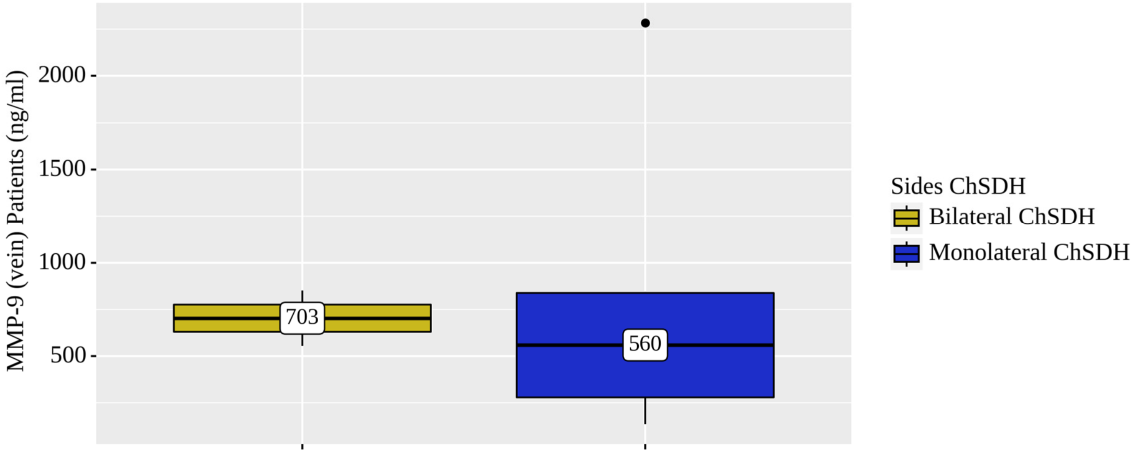

Figure 49 – Analysis of MMP-9 (vein) Patients conditioning on Sides ChSDH

When evaluating the dependence of the probability of Monolateral ChSDH on the MMP-9 (vein) Patients using the ROC analysis, the following curve was obtained.

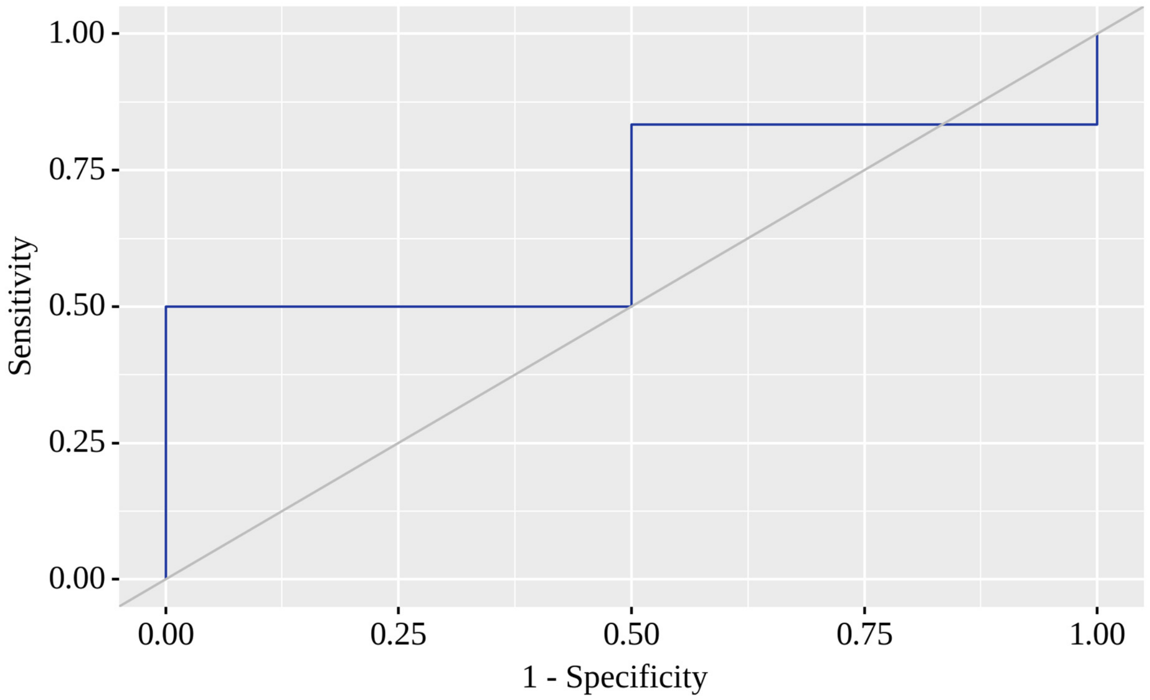

Figure 50 – ROC-curve characterizing the dependence of the probability Sides ChSDH on MMP-9 (vein) Patients

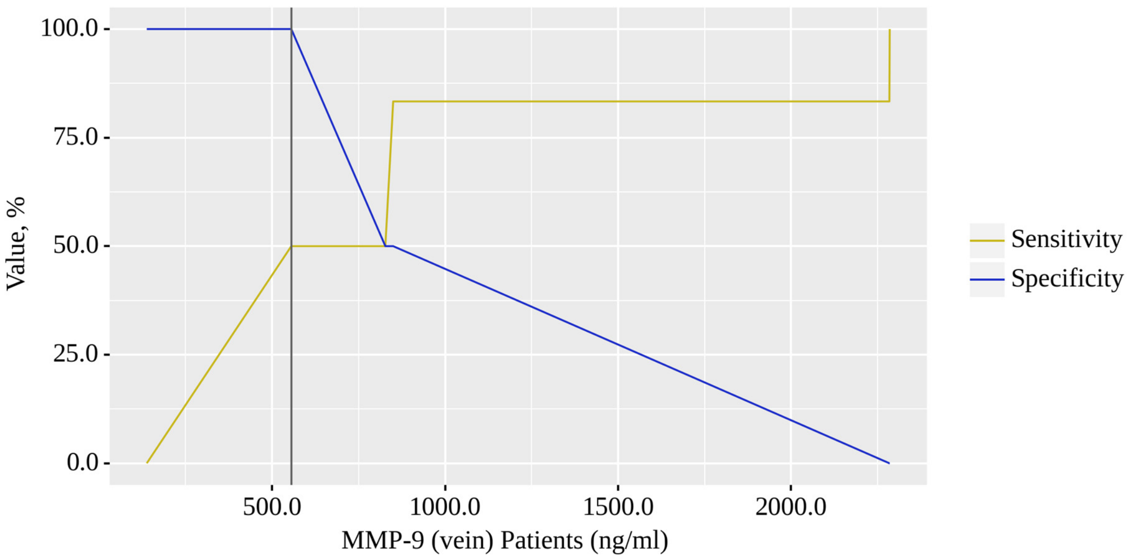

Figure 51 - Analysis of the sensitivity and specificity of Sides ChSDH depending on MMP-9 (vein) Patients

Table 37 – Threshold MMP-9 (vein) Patients

Threshold	Sensitivity (Se), %	Specificity (Sp), %	PPV	NPV
850	83.3	50.0	83.3	50.0

**827**	**50.0**	**50.0**	**75.0**	**25.0**
556	50.0	100.0	100.0	40.0

The area under the ROC curve comprised 0.667 ± 0.245 with 95% CI: 0.186 - 1.000. The resulting model was not statistically significant (p = 0.505).

The cut-off value of MMP-9 (vein) Patients which corresponds to the highest Youden's J statistic is 555.600 ng/ml. If MMP-9 (vein) Patients was less than this value, Monolateral ChSDH was predicted. The sensitivity and specificity of the method were 50.0% and 100.0%, respectively.

We performed analysis of MMP-9 (vein) Patients conditioning on Rebleeding on CT scans.

Table 38 – Analysis of MMP-9 (vein) Patients conditioning on Rebleeding on CT scans

Variable	Categories	MMP-9 (vein) Patients (ng/ml)			p
		Me	$Q_1 – Q_3$	n	
Rebleeding on CT scans	none	284	242 – 358	4	0.021*
	rebleeded	846	838 – 1208	4	

* – differences are statistically significant (p < 0.05)

In accordance with the presented table, when comparing of MMP-9 (vein) Patients, statistically significant differences were revealed depending on Rebleeding on CT scans (p = 0.021) *(applied method: Mann-Whitney U-test)*.

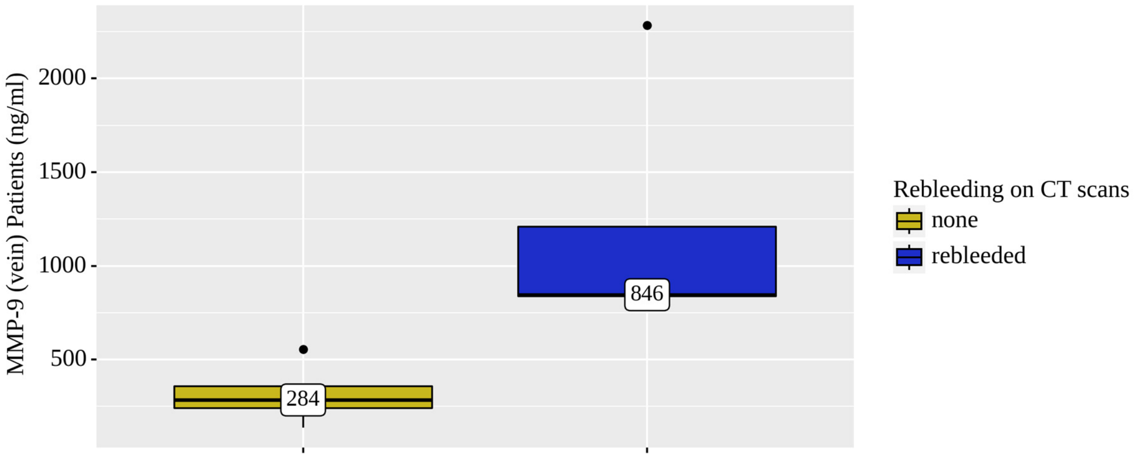

Figure 52 – Analysis of MMP-9 (vein) Patients conditioning on Rebleeding on CT scans

When evaluating the dependence of the probability of rebleeded on the MMP-9 (vein) Patients using the ROC analysis, the following curve was obtained.

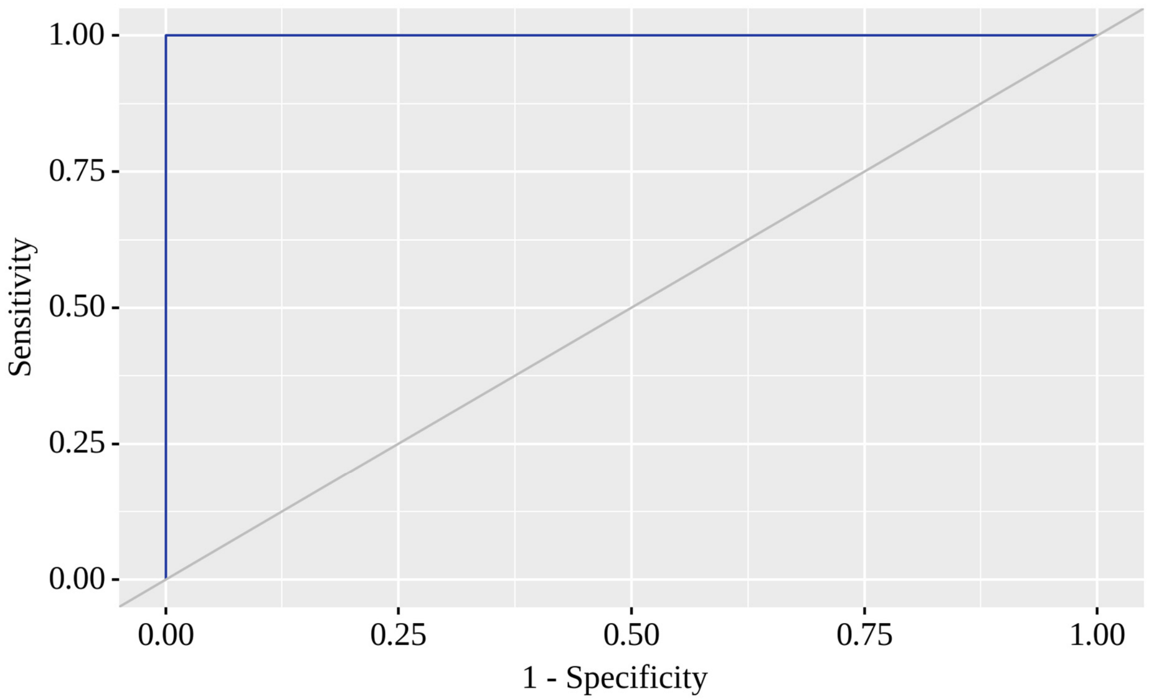

Figure 53 – ROC-curve characterizing the dependence of the probability Rebleeding on CT scans on MMP-9 (vein) Patients

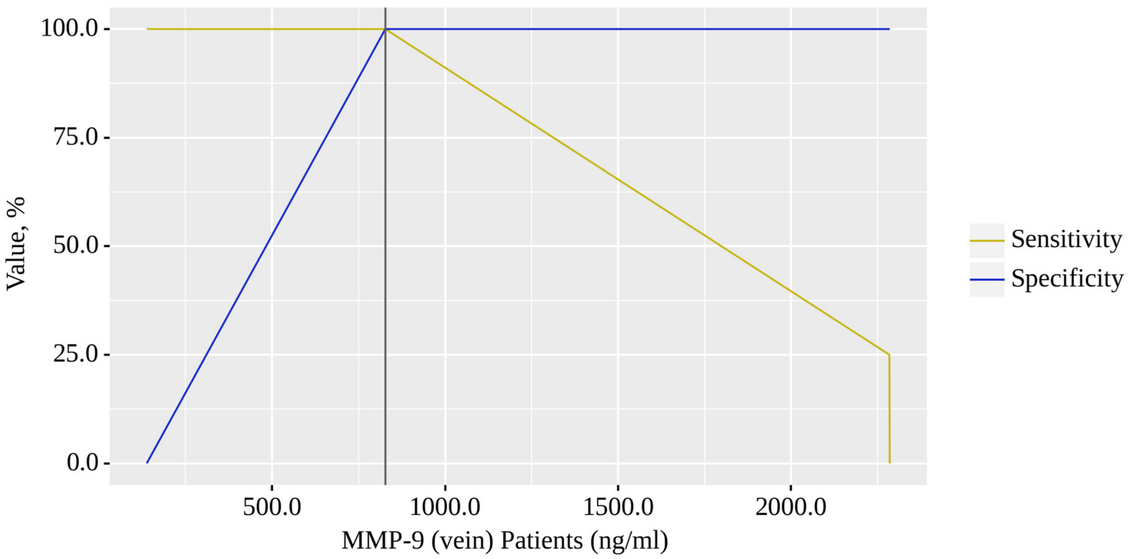

Figure 54 - Analysis of the sensitivity and specificity of Rebleeding on CT scans depending on MMP-9 (vein) Patients

Table 39 – Threshold MMP-9 (vein) Patients

Threshold	Sensitivity (Se), %	Specificity (Sp), %	PPV	NPV
**827**	**100.0**	**100.0**	**100.0**	**100.0**

The area under the ROC curve comprised 1.000 ± 0.000 with 95% CI: 1.000 - 1.000. The resulting model was statistically significant (p = 0.021).

The cut-off value of MMP-9 (vein) Patients which corresponds to the highest Youden's J statistic is 827.400 ng/ml. If MMP-9 (vein) Patients was greater than or equal to this value, rebleeded was predicted. The sensitivity and specificity of the method were 100.0% and 100.0%, respectively.

Correlation analysis of the association between MMP-9 (vein) Patients and Total volume Pre Embol ChSDs was performed.

Table 40 – Results of the correlation analysis of the association between MMP-9 (vein) Patients and Total volume Pre Embol ChSDs

Variable	Correlation characteristics		
	ρ	Strength of the association assesed using Chaddock scale	p
MMP-9 (vein) Patients – Total volume Pre Embol ChSDs	0.695	Close	0.056

A close correlation positive association between Total volume Pre Embol ChSDs and MMP-9 (vein) Patients was estimated.

Observed dependence of Total volume Pre Embol ChSDs from MMP-9 (vein) Patients is described by a linear regression equation:

$$Y_{\text{Total volume Pre Embol ChSDs}} = 0.054 \times X_{\text{MMP-9 (vein) Patients}} + 61.546$$

With an 1 ng/ml increase of MMP-9 (vein) Patients 0.054 ml change of Total volume Pre Embol ChSDs should be expected. According to the coefficient of determination R^2 of the resulting model, 30.6% of the observed variance of Total volume Pre Embol ChSDs were explained..

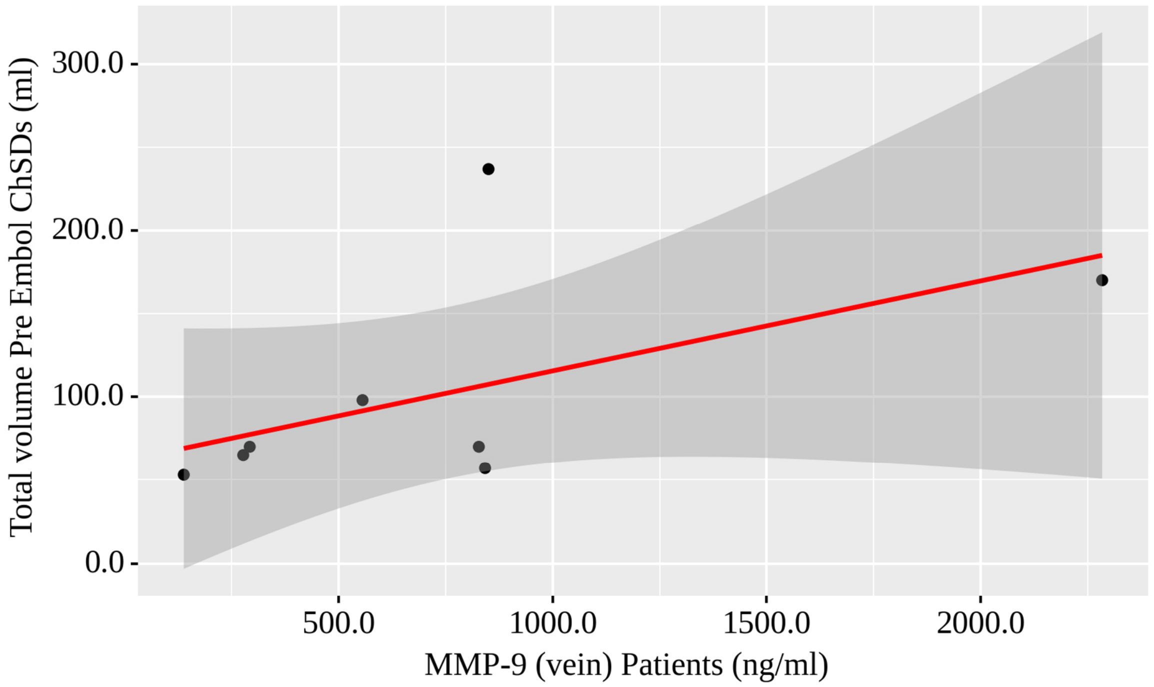

Figure 55 – Regression line characterizing the dependence of Total volume Pre Embol ChSDs from MMP-9 (vein) Patients

Analysis of MMP-9 (vein) Patients was performed conditioning on Surgery.

Table 41 – Analysis of MMP-9 (vein) Patients conditioning on Surgery

Variable	Categories	MMP-9 (vein) Patients (ng/ml)			p
		Me	$Q_1 - Q_3$	n	
Surgery	none	699	451 – 1202	4	0.773
	operated	560	288 – 833	4	

When comparing of MMP-9 (vein) Patients depending on Surgery no statistically significant differences were revealed ($p = 0.773$) *(applied method: Mann-Whitney U-test)*.

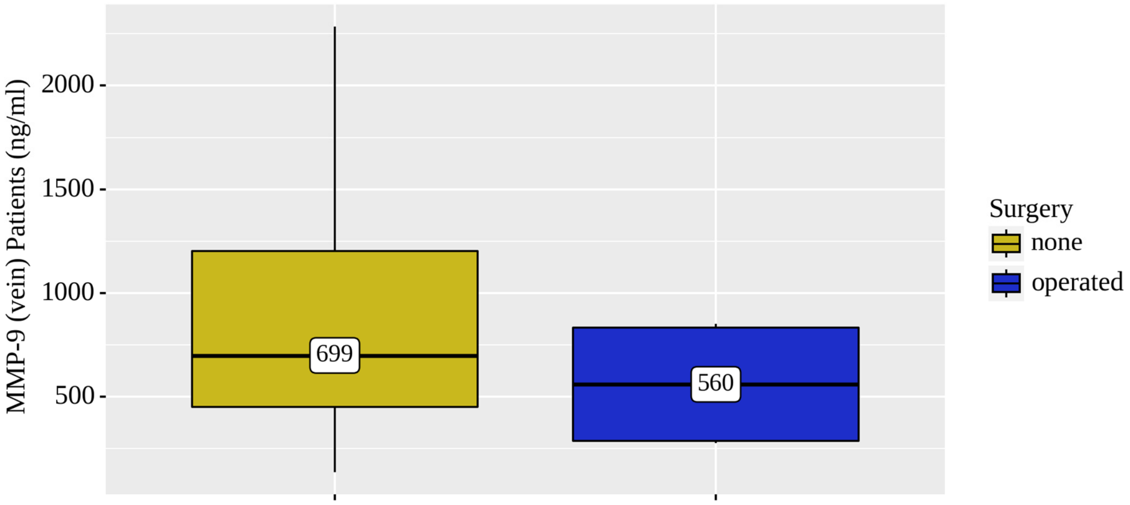

Figure 56 – Analysis of MMP-9 (vein) Patients conditioning on Surgery

When evaluating the dependence of the probability of operated on the MMP-9 (vein) Patients using the ROC analysis, the following curve was obtained.

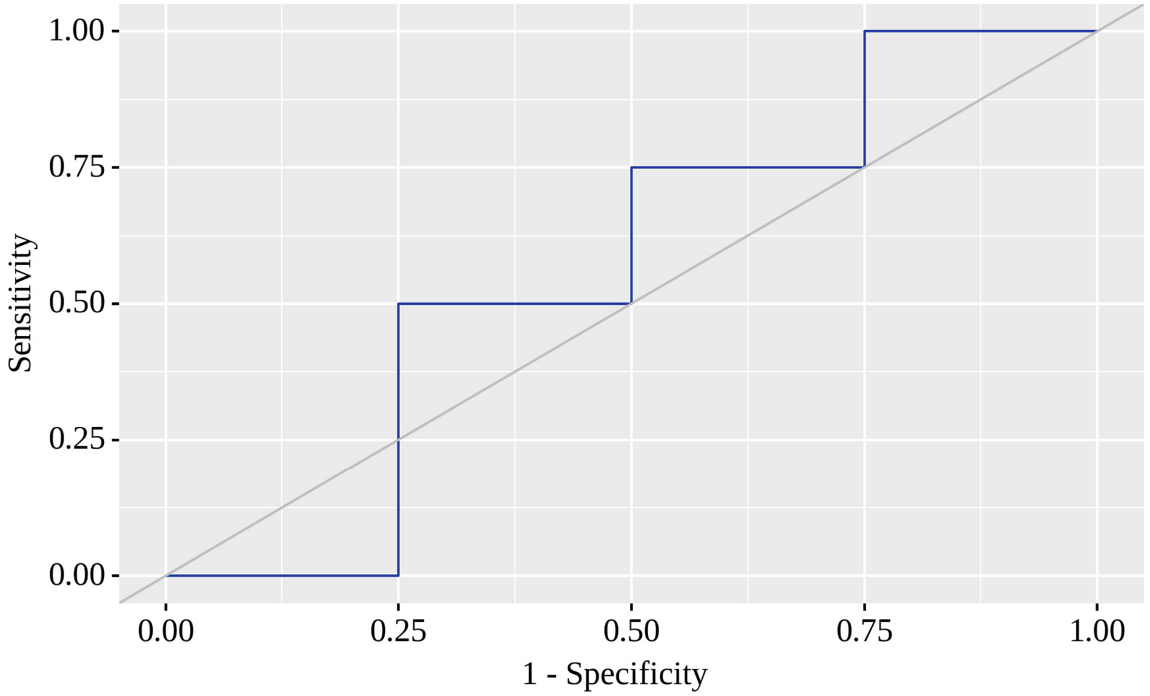

Figure 57 – ROC-curve characterizing the dependence of the probability Surgery on MMP-9 (vein) Patients

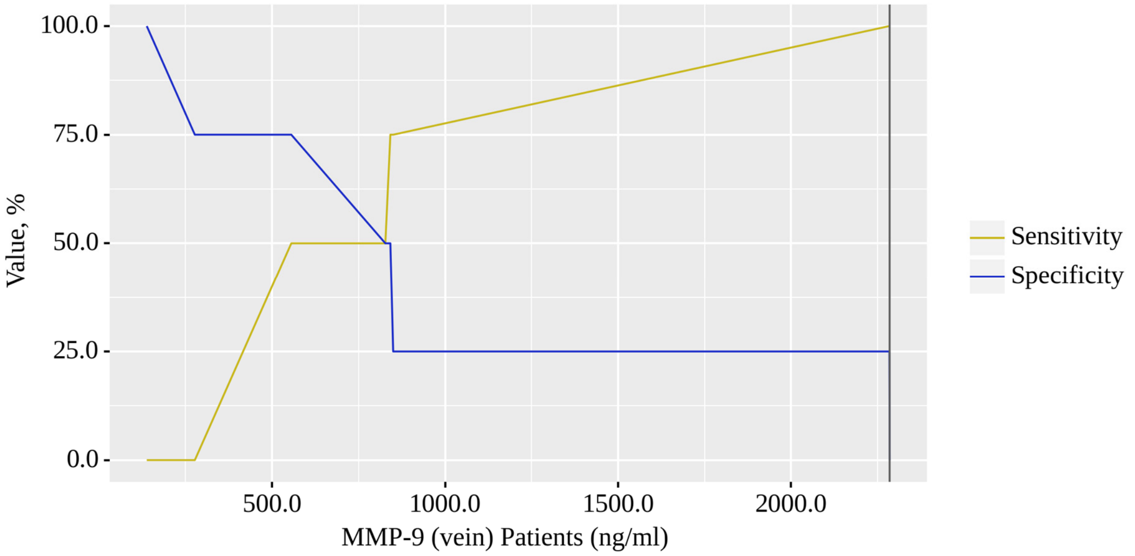

Figure 58 - Analysis of the sensitivity and specificity of Surgery depending on MMP-9 (vein) Patients

Table 42 – Threshold MMP-9 (vein) Patients

Threshold	Sensitivity (Se), %	Specificity (Sp), %	PPV	NPV
842	75.0	50.0	60.0	66.7
**827**	**50.0**	**50.0**	**50.0**	**50.0**
556	50.0	75.0	66.7	60.0

The area under the ROC curve comprised 0.562 ± 0.215 with 95% CI: 0.142 - 0.983. The resulting model was not statistically significant (p = 0.773).

The cut-off value of MMP-9 (vein) Patients which corresponds to the highest Youden's J statistic is 2284.000 ng/ml. If MMP-9 (vein) Patients was less than this value, operated was predicted. The sensitivity and specificity of the method were 100.0% and 25.0%, respectively.

We performed a correlation analysis of the association between MMP-9 (artery) Patients and MMP-9 (vein) Patients.

Table 43 – Results of the correlation analysis of the association between MMP-9 (artery) Patients and MMP-9 (vein) Patients

Variable	Correlation characteristics		
	ρ	Strength of the association assesed using Chaddock scale	p

MMP-9 (artery) Patients – MMP-9 (vein) Patients	0.750	Strong	0.052

A strong correlation positive association between MMP-9 (vein) Patients and MMP-9 (artery) Patients was estimated.

Observed dependence of MMP-9 (vein) Patients from MMP-9 (artery) Patients is described by a linear regression equation:

$$Y_{\text{MMP-9 (vein) Patients}} = 0.877 \times X_{\text{MMP-9 (artery) Patients}} + 264.971$$

With an 1 ng/ml increase of MMP-9 (artery) Patients 0.877 ng/ml change of MMP-9 (vein) Patients should be expected. According to the coefficient of determination R^2 of the resulting model, 25.2% of the observed variance of MMP-9 (vein) Patients were explained..

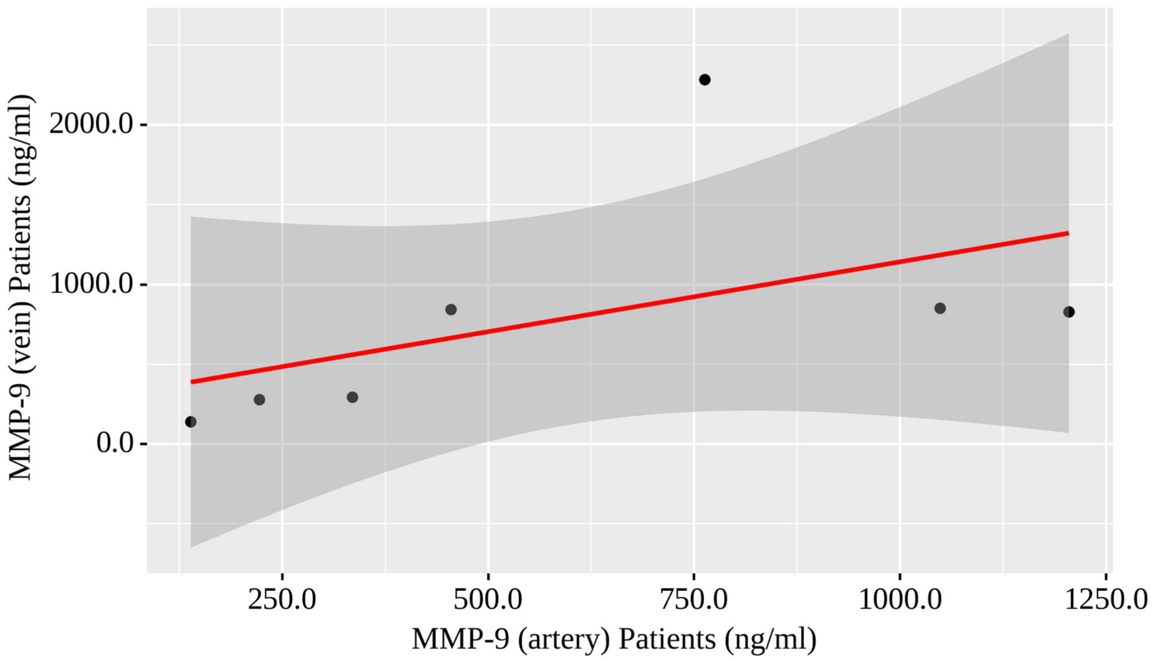

Figure 59 – Regression line characterizing the dependence of MMP-9 (vein) Patients from MMP-9 (artery) Patients

Analysis of MMP-9 (artery) Patients was performed conditioning on Sides ChSDH.

Table 44 – Analysis of MMP-9 (artery) Patients conditioning on Sides ChSDH

Variable	Categories	MMP-9 (artery) Patients (ng/ml)			p
		Me	$Q_1 – Q_3$	n	

Sides ChSDH	Bilateral ChSDH	1049	1049 – 1049	1	0.317
	Monolateral ChSDH	395	251 – 686	6	

When comparing of MMP-9 (artery) Patients depending on Sides ChSDH no statistically significant differences were revealed (p = 0.317) *(applied method: Mann-Whitney U-test)*.

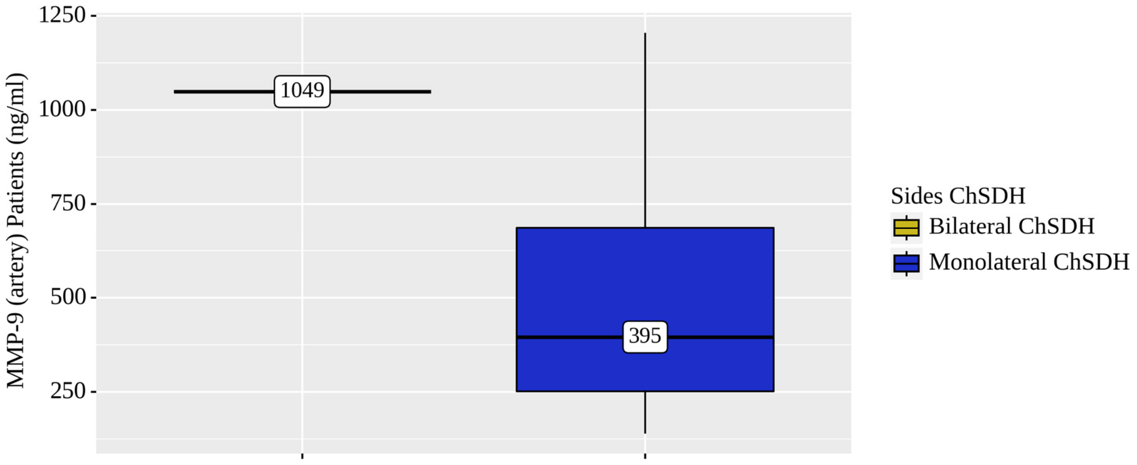

Figure 60 – Analysis of MMP-9 (artery) Patients conditioning on Sides ChSDH

When evaluating the dependence of the probability of Monolateral ChSDH on the MMP-9 (artery) Patients using the ROC analysis, the following curve was obtained.

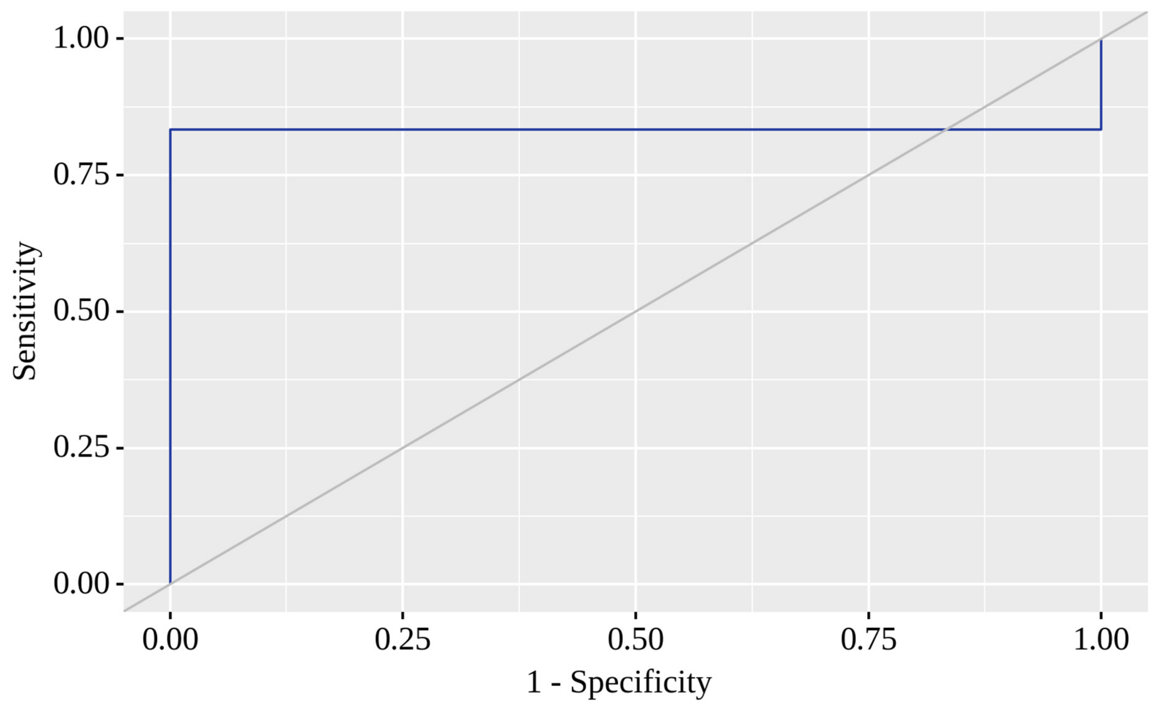

Figure 61 – ROC-curve characterizing the dependence of the probability Sides ChSDH on MMP-9 (artery) Patients

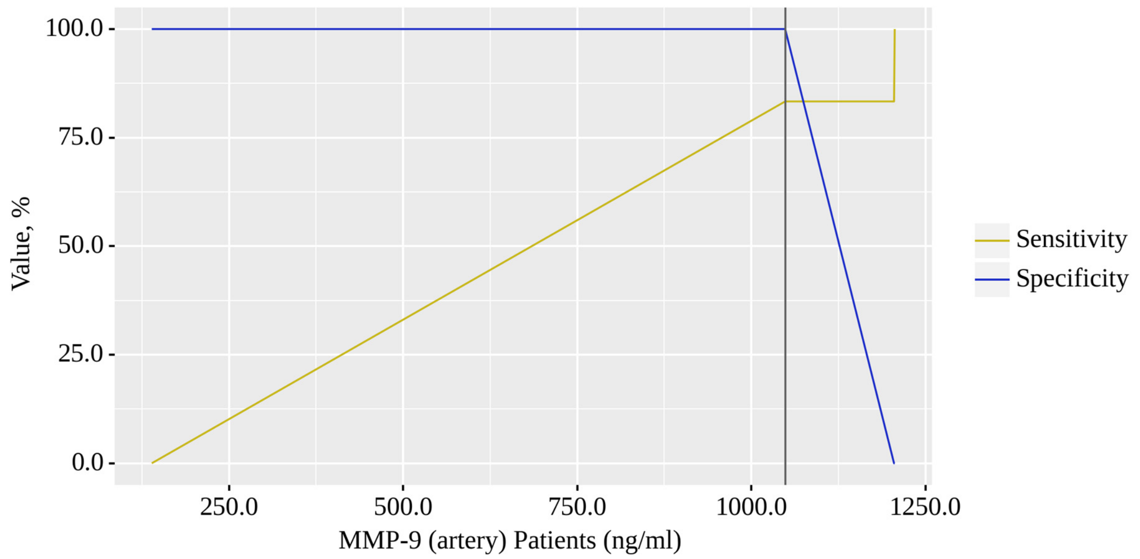

Figure 62 - Analysis of the sensitivity and specificity of Sides ChSDH depending on MMP-9 (artery) Patients

Table 45 – Threshold MMP-9 (artery) Patients

Threshold	Sensitivity (Se), %	Specificity (Sp), %	PPV	NPV
**1049**	**83.3**	**100.0**	**100.0**	**50.0**

The area under the ROC curve comprised 0.833 ± 0.275 with 95% CI: 0.294 - 1.000. The resulting model was not statistically significant (p = 0.317).

The cut-off value of MMP-9 (artery) Patients which corresponds to the highest Youden's J statistic is 1048.600 ng/ml. If MMP-9 (artery) Patients was less than this value, Monolateral ChSDH was predicted. The sensitivity and specificity of the method were 83.3% and 100.0%, respectively.

We performed a correlation analysis of the association between MMP-9 (artery) Patients and Total volume Pre Embol ChSDs.

Table 46 – Results of the correlation analysis of the association between MMP-9 (artery) Patients and Total volume Pre Embol ChSDs

Variable	Correlation characteristics		
	ρ	Strength of the association assesed using Chaddock scale	p
MMP-9 (artery) Patients – Total volume Pre Embol ChSDs	0.721	Strong	0.068

A strong correlation positive association between Total volume Pre Embol ChSDs and MMP-9 (artery) Patients was estimated.

Observed dependence of Total volume Pre Embol ChSDs from MMP-9 (artery) Patients is described by a linear regression equation:

$$Y_{\text{Total volume Pre Embol ChSDs}} = 0.1 \times X_{\text{MMP-9 (artery) Patients}} + 43.639$$

With an 1 ng/ml increase of MMP-9 (artery) Patients 0.1 ml change of Total volume Pre Embol ChSDs should be expected. According to the coefficient of determination R^2 of the resulting model, 33.8% of the observed variance of Total volume Pre Embol ChSDs were explained..

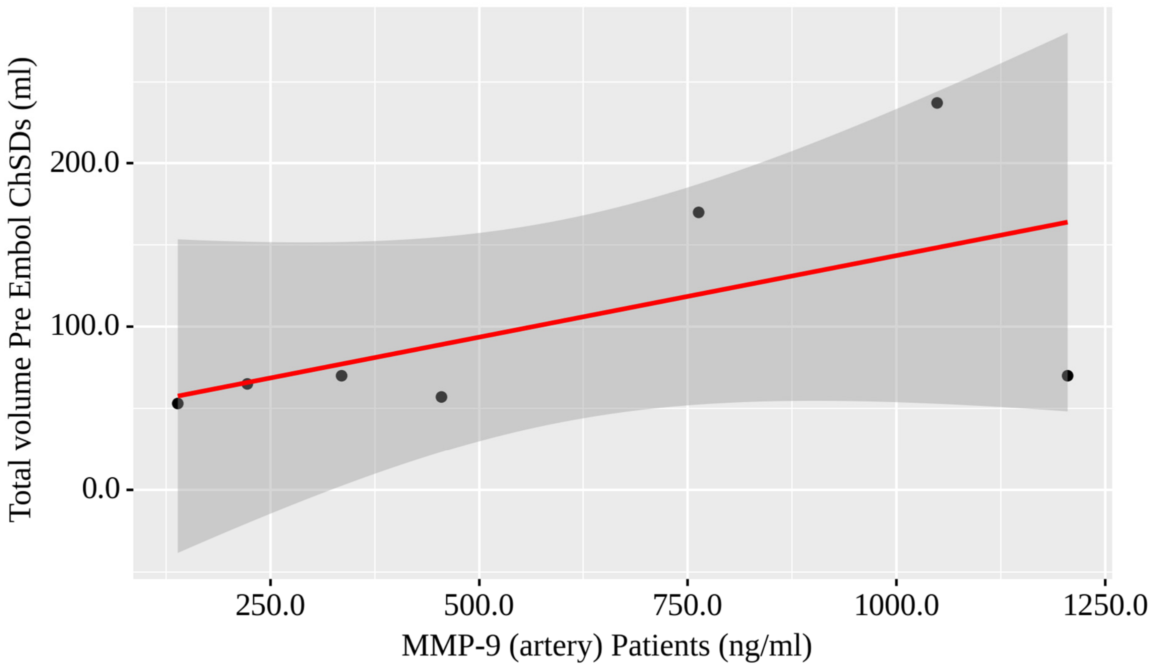

Figure 63 – Regression line characterizing the dependence of Total volume Pre Embol ChSDs from MMP-9 (artery) Patients

Analysis of MMP-9 (artery) Patients was performed conditioning on Rebleeding on CT scans.

Table 47 – Analysis of MMP-9 (artery) Patients conditioning on Rebleeding on CT scans

Variable	Categories	MMP-9 (artery) Patients (ng/ml)			p
		M ± SD	95% CI	n	
Rebleeding on CT scans	none	232 ± 98	-12 – 477	3	0.025*
	rebleeded	868 ± 331	342 – 1394	4	

* – differences are statistically significant ($p < 0.05$)

Statistically significant differences were revealed when comparing of MMP-9 (artery) Patients depending on Rebleeding on CT scans ($p = 0.025$) *(applied method: Student's t-test)*.

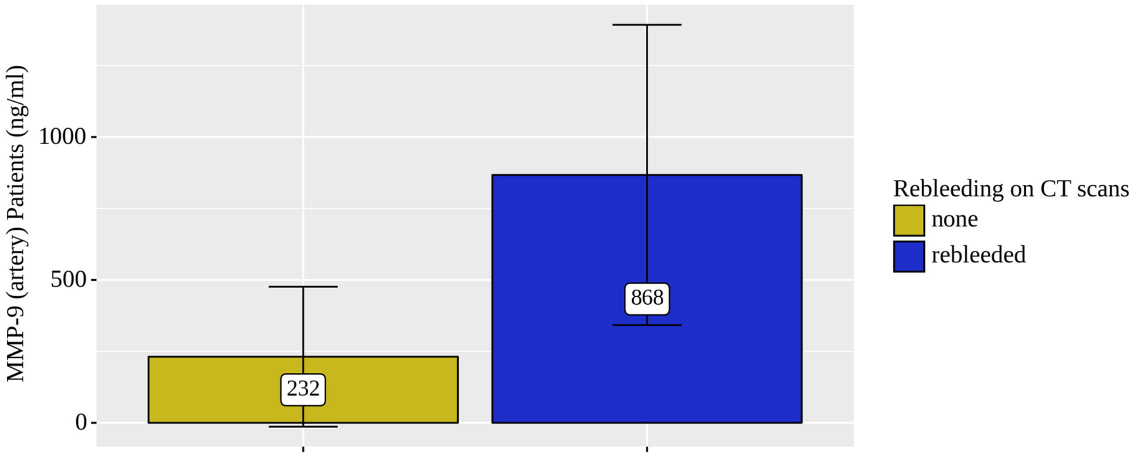

Figure 64 – Analysis of MMP-9 (artery) Patients conditioning on Rebleeding on CT scans

When evaluating the dependence of the probability of rebleeded on the MMP-9 (artery) Patients using the ROC analysis, the following curve was obtained.

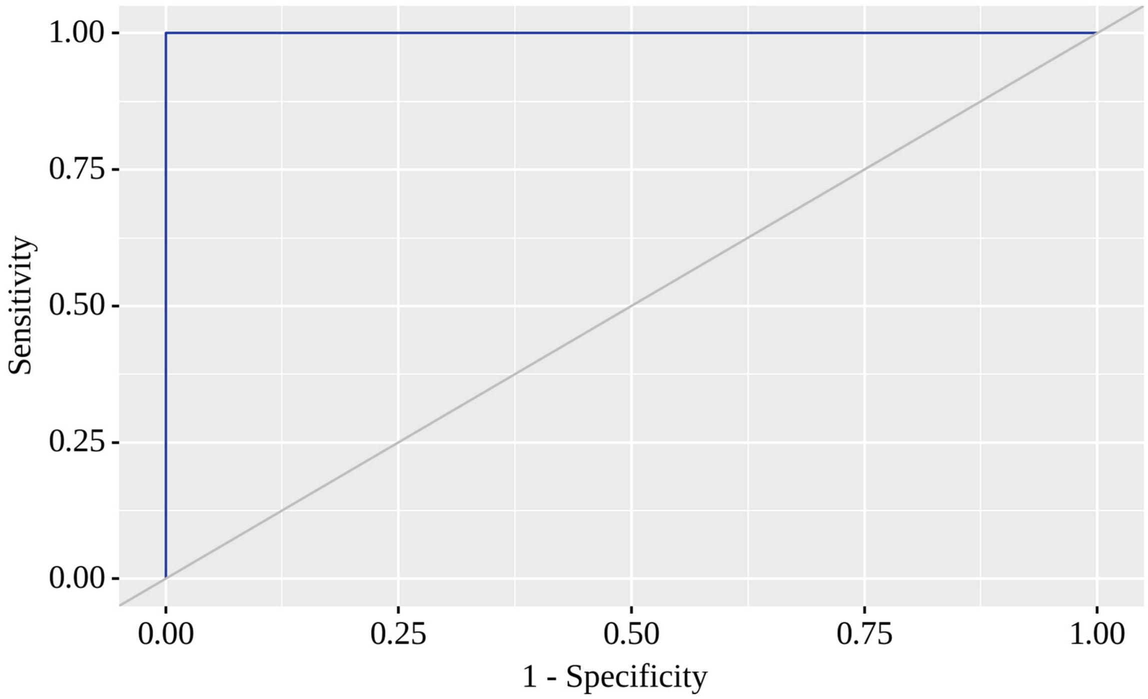

Figure 65 – ROC-curve characterizing the dependence of the probability Rebleeding on CT scans on MMP-9 (artery) Patients

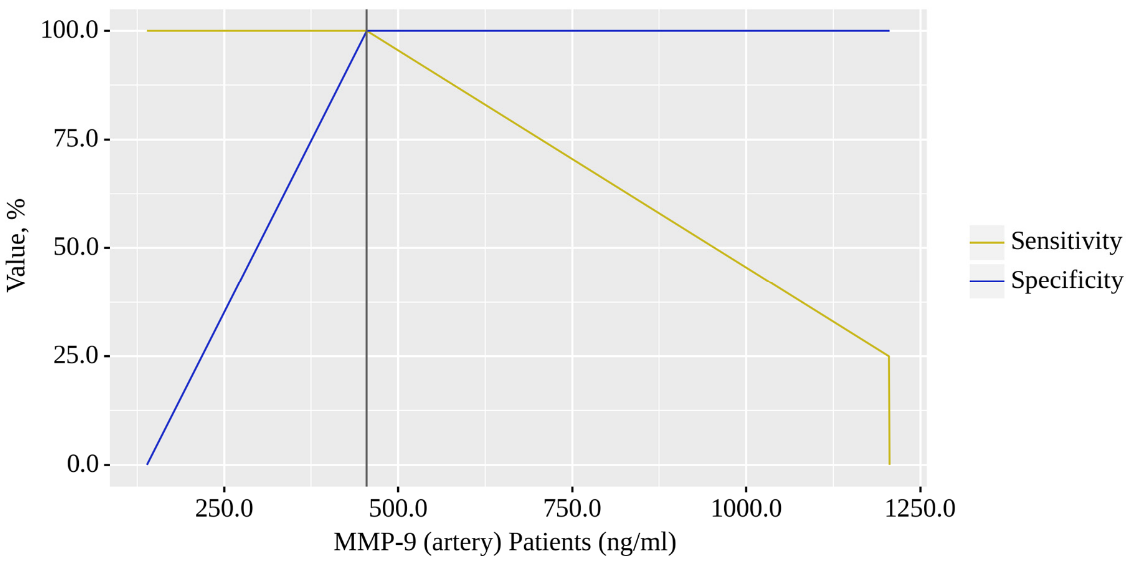

Figure 66 - Analysis of the sensitivity and specificity of Rebleeding on CT scans depending on MMP-9 (artery) Patients

Table 48 – Threshold MMP-9 (artery) Patients

Threshold	Sensitivity (Se), %	Specificity (Sp), %	PPV	NPV
**455**	**100.0**	**100.0**	**100.0**	**100.0**

The area under the ROC curve comprised 1.000 ± 0.000 with 95% CI: 1.000 - 1.000. The resulting model was statistically significant (p = 0.034).

The cut-off value of MMP-9 (artery) Patients which corresponds to the highest Youden's J statistic is 455.000 ng/ml. If MMP-9 (artery) Patients was greater than or equal to this value, rebleeded was predicted. The sensitivity and specificity of the method were 100.0% and 100.0%, respectively.

We performed analysis of MMP-9 (artery) Patients conditioning on Surgery.

Table 49 – Analysis of MMP-9 (artery) Patients conditioning on Surgery

Variable	Categories	MMP-9 (artery) Patients (ng/ml)			p
		M ± SD	95% CI	n	
Surgery	none	452 ± 312	-322 – 1227	3	0.482
	operated	703 ± 496	-86 – 1492	4	

When comparing of MMP-9 (artery) Patients depending on Surgery no statistically significant differences were revealed (p = 0.482) *(applied method: Student's t-test)*.

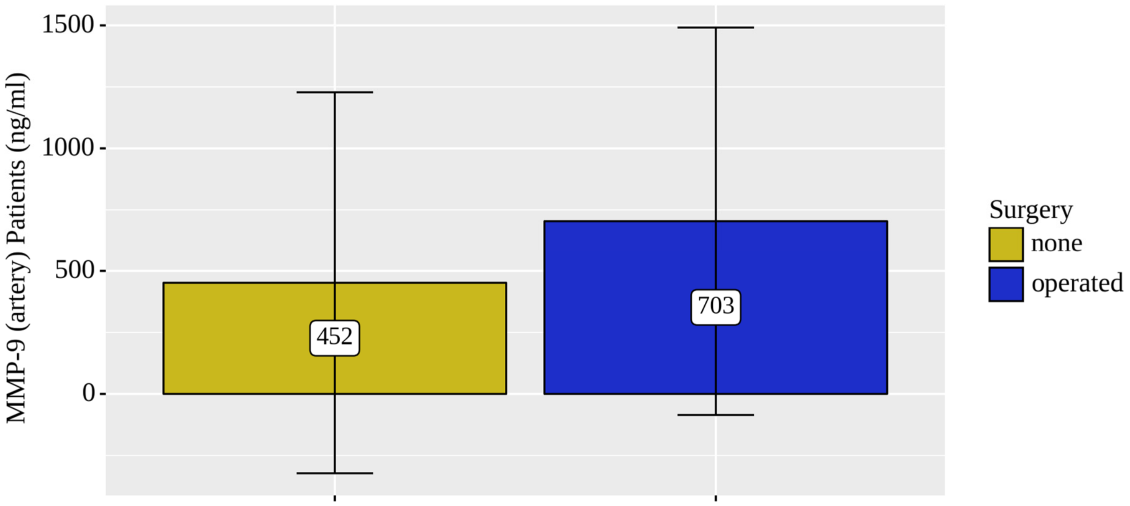

Figure 67 – Analysis of MMP-9 (artery) Patients conditioning on Surgery

When evaluating the dependence of the probability of operated on the MMP-9 (artery) Patients using the ROC analysis, the following curve was obtained.

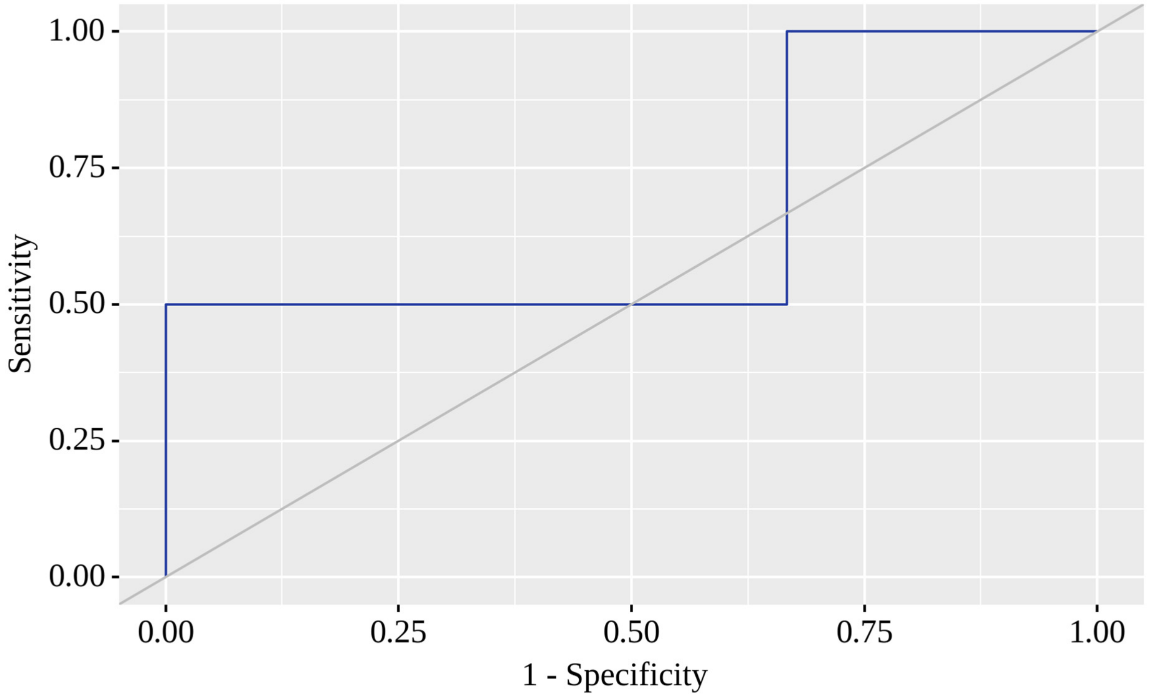

Figure 68 – ROC-curve characterizing the dependence of the probability Surgery on MMP-9 (artery) Patients

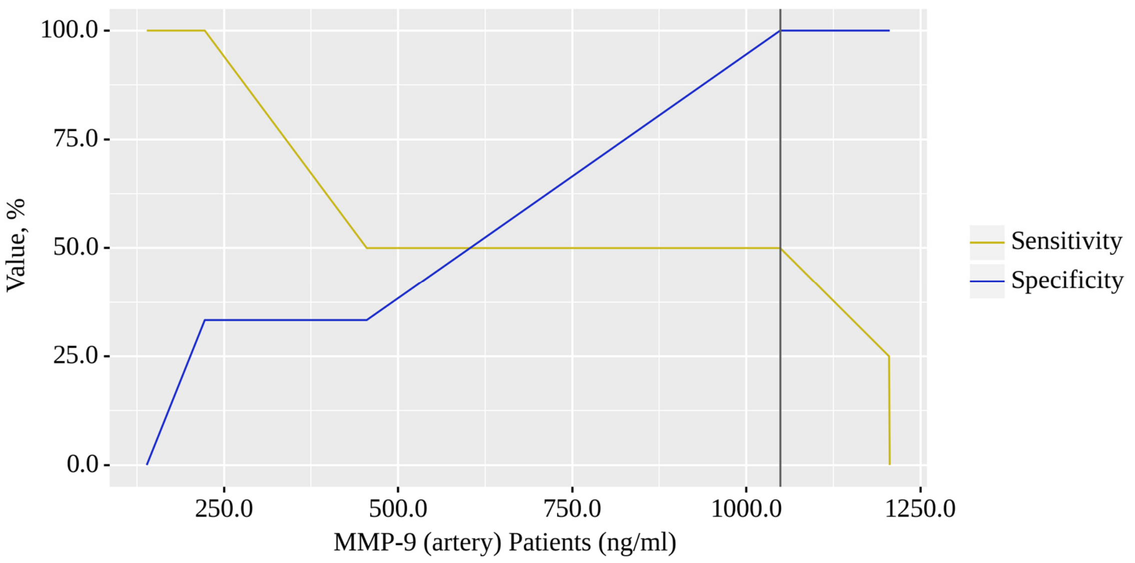

Figure 69 - Analysis of the sensitivity and specificity of Surgery depending on MMP-9 (artery) Patients

Table 50 – Threshold MMP-9 (artery) Patients

Threshold	Sensitivity (Se), %	Specificity (Sp), %	PPV	NPV
**1049**	**50.0**	**100.0**	**100.0**	**60.0**

The area under the ROC curve comprised 0.667 ± 0.217 with 95% CI: 0.241 - 1.000. The resulting model was not statistically significant (p = 0.480).

The cut-off value of MMP-9 (artery) Patients which corresponds to the highest Youden's J statistic is 1048.600 ng/ml. If MMP-9 (artery) Patients was greater than or equal to this value, operated was predicted. The sensitivity and specificity of the method were 50.0% and 100.0%, respectively.

Analysis of Angio-2 (vein) P/HV was performed conditioning on Patients/Healthy volunteers.

Table 51 – Analysis of Angio-2 (vein) P/HV conditioning on Patients/Healthy volunteers

Variable	Categories	Angio-2 (vein) P/HV (pg/ml)			p
		Me	$Q_1 – Q_3$	n	
Patients/Healthy volunteers	patient	2608	2292 – 5182	8	0.023*
	healthy volunteer	2186	2048 – 2400	33	

* – differences are statistically significant (p < 0.05)

In accordance with the presented table, when comparing of Angio-2 (vein) P/HV, statistically significant differences were revealed depending on Patients/Healthy volunteers (p = 0.023) *(applied method: Mann-Whitney U-test)*.

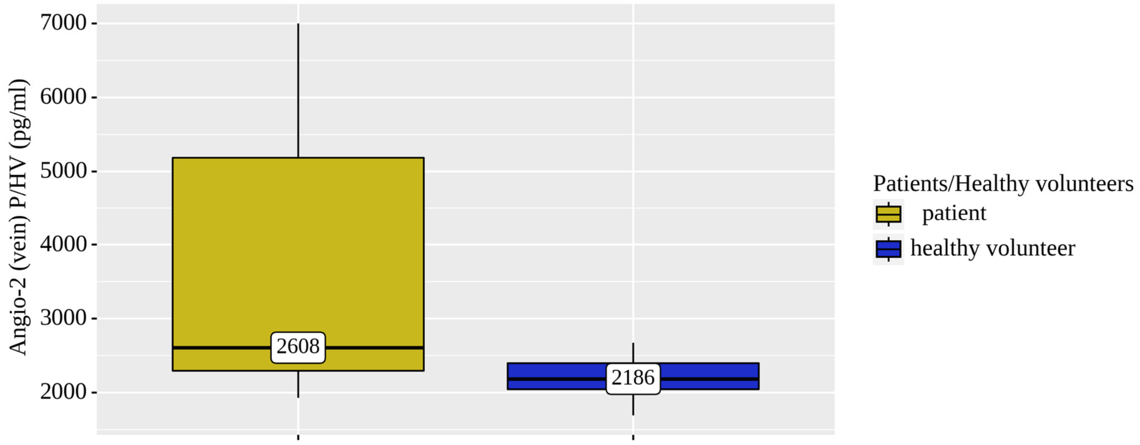

Figure 70 – Analysis of Angio-2 (vein) P/HV conditioning on Patients/Healthy volunteers

When evaluating the dependence of the probability of healthy volunteer on the Angio-2 (vein) P/HV using the ROC analysis, the following curve was obtained.

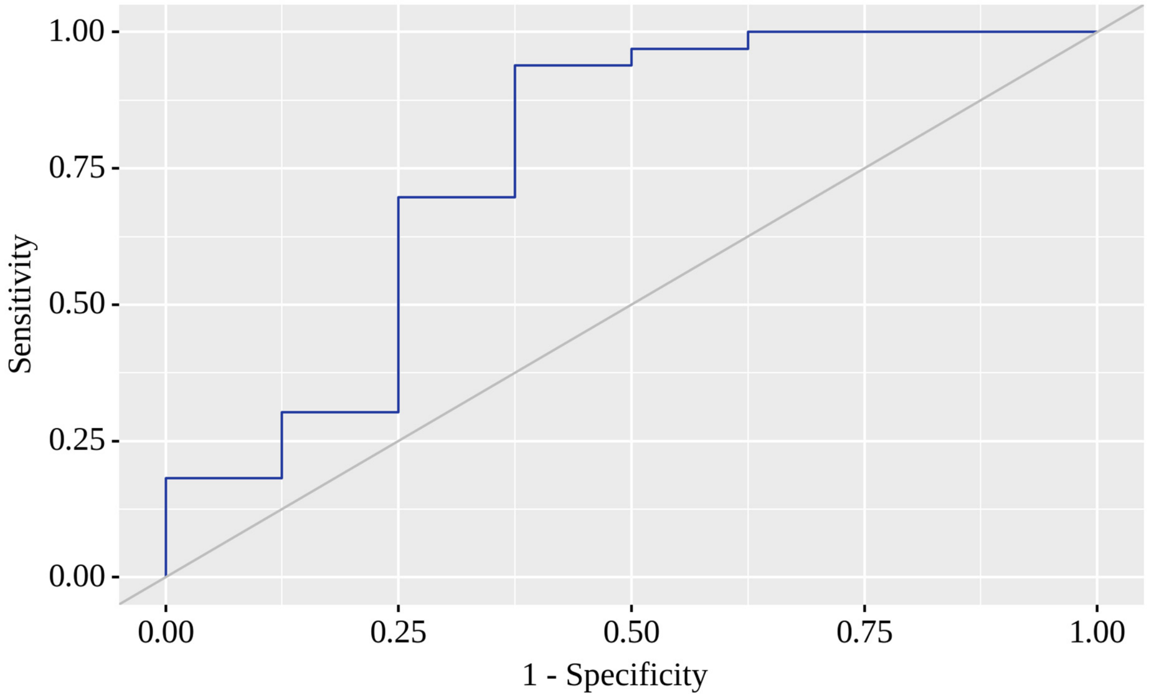

Figure 71 – ROC-curve characterizing the dependence of the probability Patients/Healthy volunteers on Angio-2 (vein) P/HV

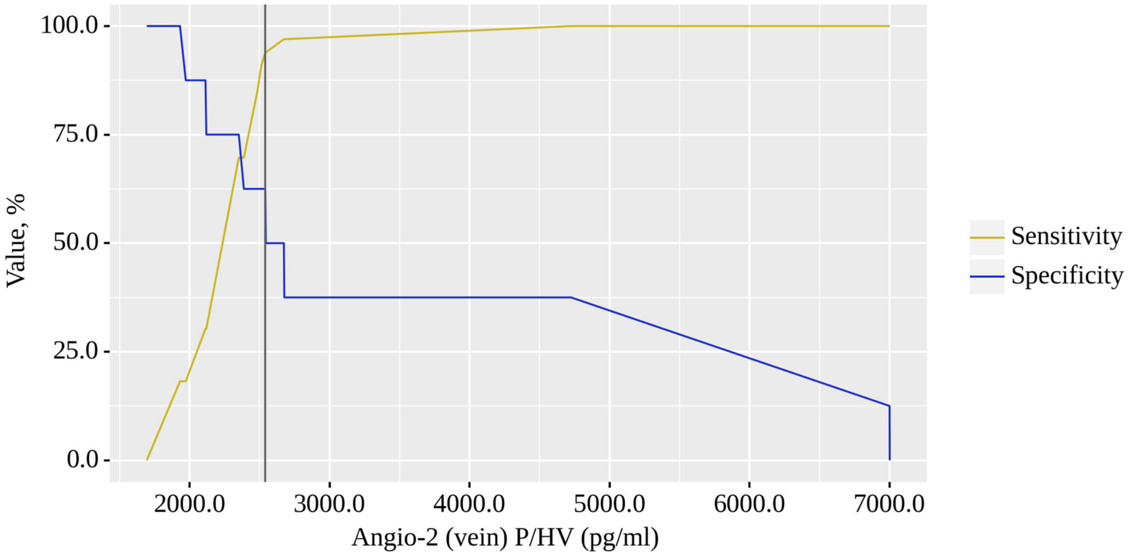

Figure 72 - Analysis of the sensitivity and specificity of Patients/Healthy volunteers depending on Angio-2 (vein) P/HV

Table 52 – Threshold Angio-2 (vein) P/HV

Threshold	Sensitivity (Se), %	Specificity (Sp), %	PPV	NPV
2674	97.0	50.0	88.9	80.0
2545	93.9	50.0	88.6	66.7
2541	93.9	62.5	91.2	71.4
2513	90.9	62.5	90.9	62.5
2484	84.8	62.5	90.3	50.0
2388	69.7	62.5	88.5	33.3
**2352**	**69.7**	**75.0**	**92.0**	**37.5**

The area under the ROC curve comprised 0.761 ± 0.106 with 95% CI: 0.555 - 0.968. The resulting model was statistically significant (p = 0.023).

The cut-off value of Angio-2 (vein) P/HV which corresponds to the highest Youden's J statistic is 2541.000 pg/ml. If Angio-2 (vein) P/HV was less than this value, healthy volunteer was predicted. The sensitivity and specificity of the method were 93.9% and 62.5%, respectively.

We performed analysis of Angio-2 (vein) Patients conditioning on Sides ChSDH.

Table 53 – Analysis of Angio-2 (vein) Patients conditioning on Sides ChSDH

Variable	Categories	Angio-2 (vein) Patients (pg/ml)			p
		Me	$Q_1 – Q_3$	n	
Sides ChSDH	Bilateral ChSDH	4466	3199 – 5733	2	1.000
	Monolateral ChSDH	2608	2399 – 4212	6	

When comparing of Angio-2 (vein) Patients depending on Sides ChSDH there were no statistically significant differences (p = 1.000) *(applied method: Mann-Whitney U-test)*.

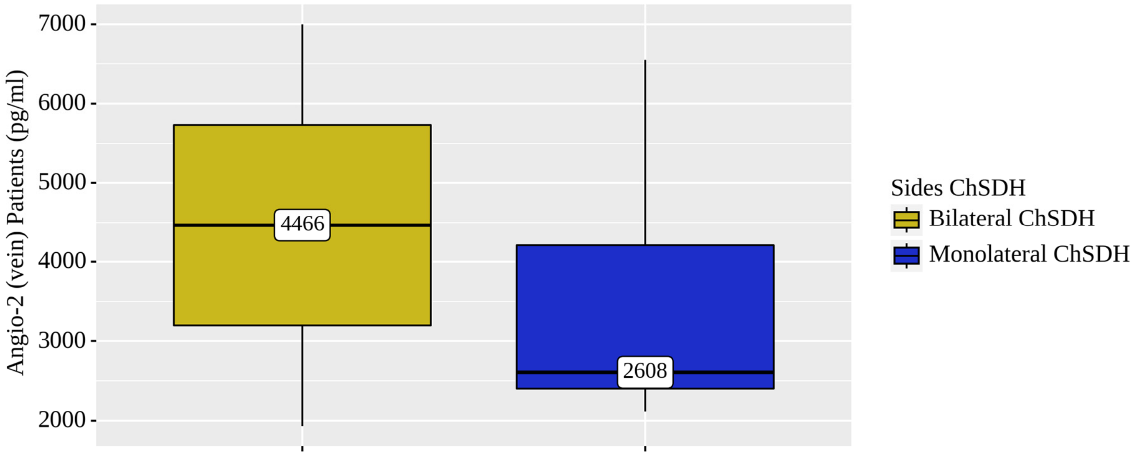

Figure 73 – Analysis of Angio-2 (vein) Patients conditioning on Sides ChSDH

When evaluating the dependence of the probability of Monolateral ChSDH on the Angio-2 (vein) Patients using the ROC analysis, the following curve was obtained.

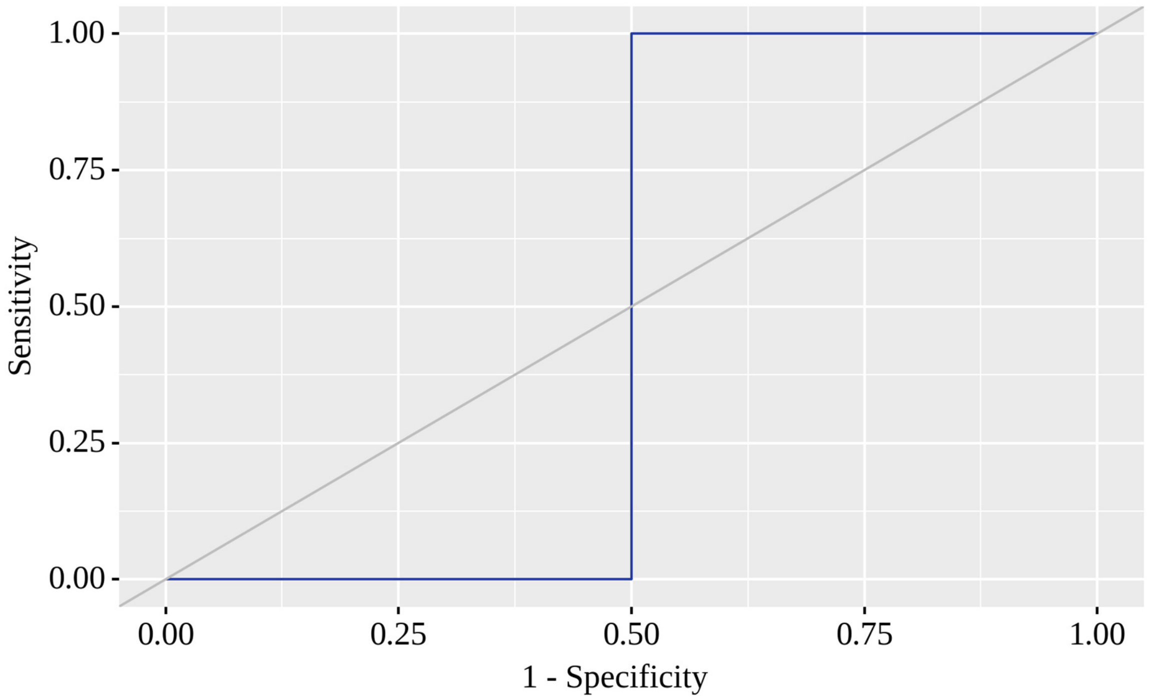

Figure 74 – ROC-curve characterizing the dependence of the probability Sides ChSDH on Angio-2 (vein) Patients

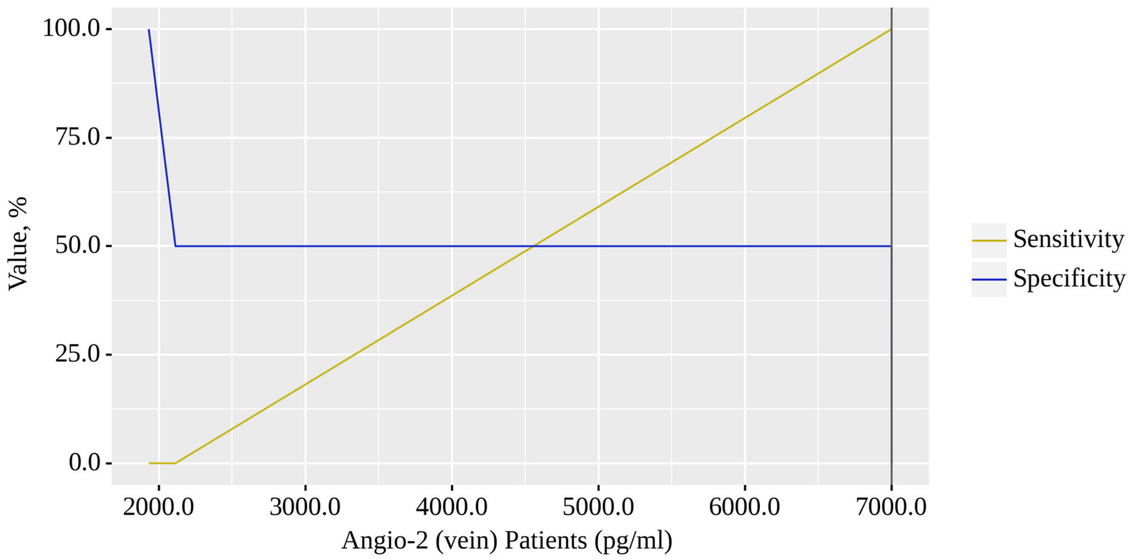

Figure 75 - Analysis of the sensitivity and specificity of Sides ChSDH depending on Angio-2 (vein) Patients

Table 54 – Threshold Angio-2 (vein) Patients

Threshold	Sensitivity (Se), %	Specificity (Sp), %	PPV	NPV
**7000**	**100.0**	**50.0**	**85.7**	**100.0**

The area under the ROC curve comprised 0.500 ± 0.250 with 95% CI: 0.010 - 0.990. The resulting model was not statistically significant (p = 1.000).

The cut-off value of Angio-2 (vein) Patients which corresponds to the highest Youden's J statistic is 7000.000 pg/ml. If Angio-2 (vein) Patients was less than this value, Monolateral ChSDH was predicted. The sensitivity and specificity of the method were 100.0% and 50.0%, respectively.

Analysis of Angio-2 (vein) Patients was performed conditioning on Rebleeding on CT scans.

Table 55 – Analysis of Angio-2 (vein) Patients conditioning on Rebleeding on CT scans

Variable	Categories	Angio-2 (vein) Patients (pg/ml)			p
		M ± SD	95% CI	n	
Rebleeding on CT scans	none	3425 ± 2110	68 – 6782	4	0.703
	rebleeded	4048 ± 2294	397 – 7698	4	

When comparing of Angio-2 (vein) Patients depending on Rebleeding on CT scans no statistically significant differences were revealed (p = 0.703) *(applied method: Student's t-test)*.

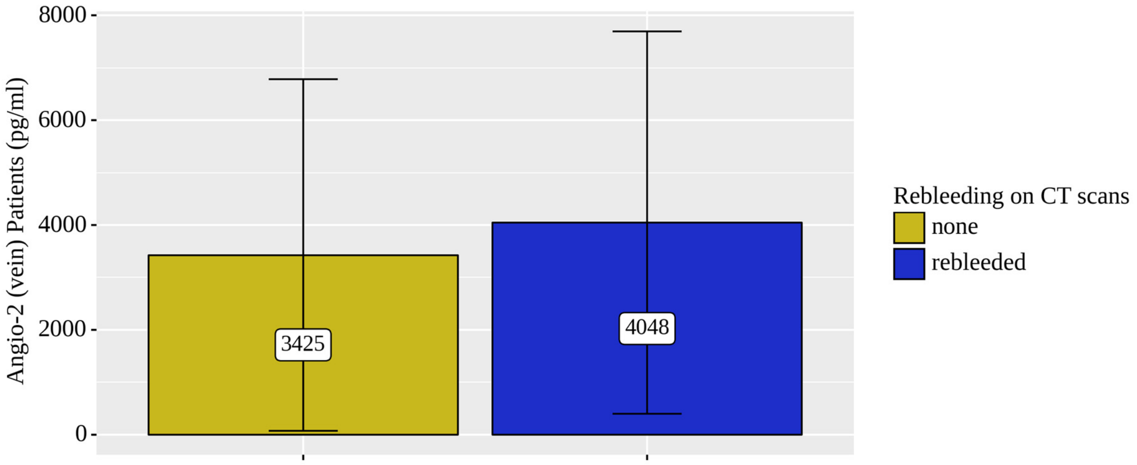

Figure 76 – Analysis of Angio-2 (vein) Patients conditioning on Rebleeding on CT scans

When evaluating the dependence of the probability of rebleeded on the Angio-2 (vein) Patients using the ROC analysis, the following curve was obtained.

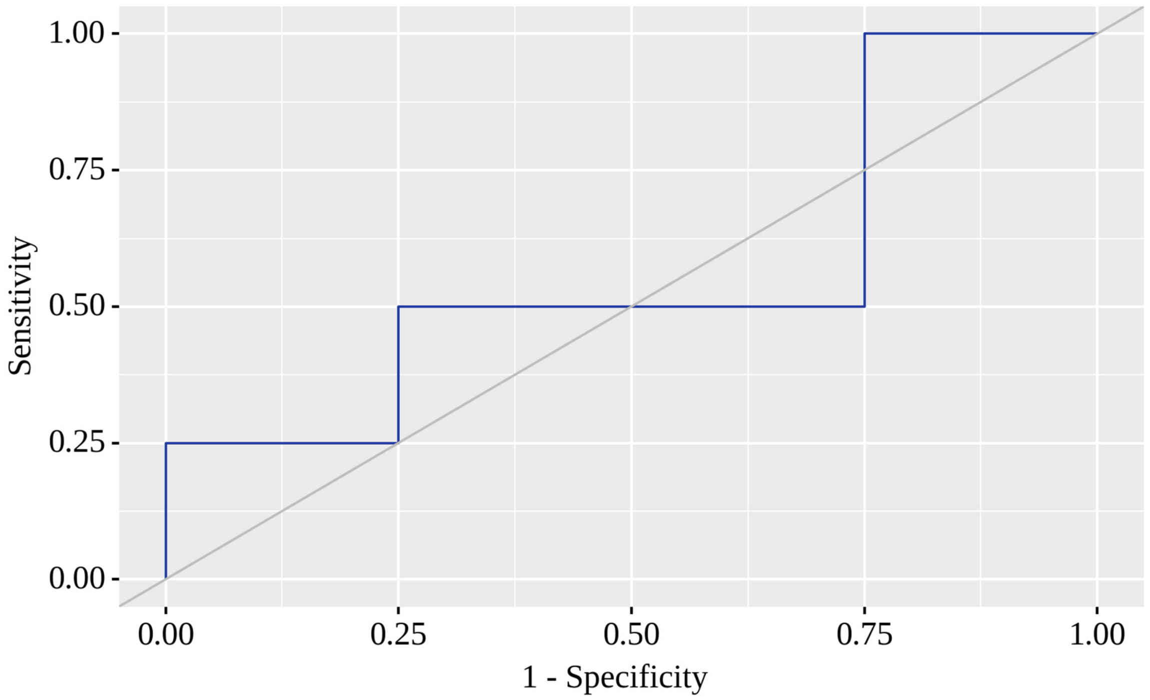

Figure 77 – ROC-curve characterizing the dependence of the probability Rebleeding on CT scans on Angio-2 (vein) Patients

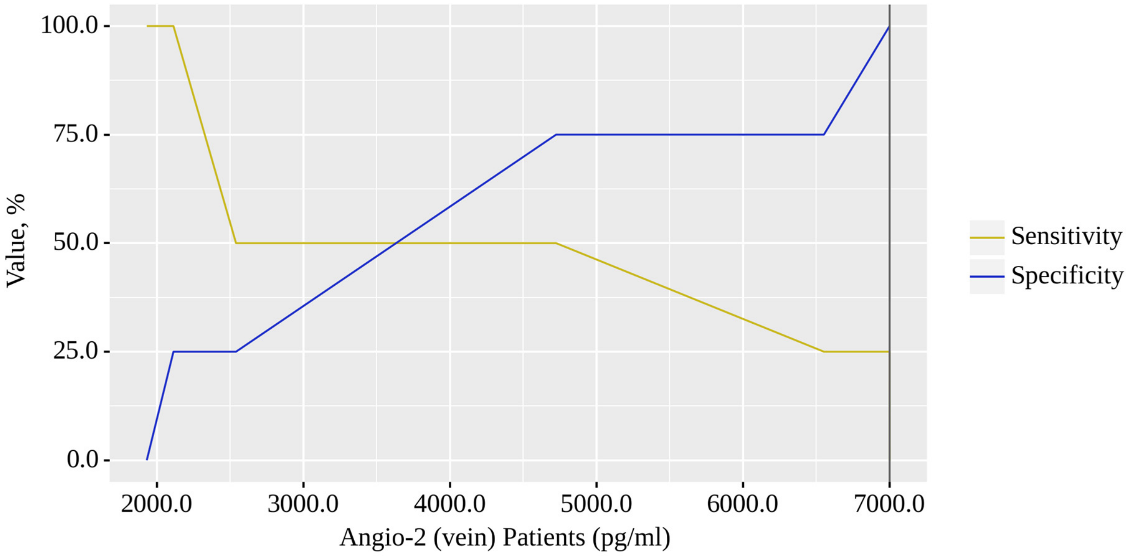

Figure 78 - Analysis of the sensitivity and specificity of Rebleeding on CT scans depending on Angio-2 (vein) Patients

Table 56 – Threshold Angio-2 (vein) Patients

Threshold	Sensitivity (Se), %	Specificity (Sp), %	PPV	NPV

**4725**	**50.0**	**75.0**	**66.7**	**60.0**

The area under the ROC curve comprised 0.562 ± 0.215 with 95% CI: 0.142 - 0.983. The resulting model was not statistically significant (p = 0.773).

The cut-off value of Angio-2 (vein) Patients which corresponds to the highest Youden's J statistic is 7000.000 pg/ml. If Angio-2 (vein) Patients was greater than or equal to this value, rebleeded was predicted. The sensitivity and specificity of the method were 25.0% and 100.0%, respectively.

Correlation analysis of the association between Angio-2 (vein) Patients and Total volume Pre Embol ChSDs was performed.

Table 57 – Results of the correlation analysis of the association between Angio-2 (vein) Patients and Total volume Pre Embol ChSDs

Variable	Correlation characteristics		
	ρ	Strength of the association assesed using Chaddock scale	p
Angio-2 (vein) Patients – Total volume Pre Embol ChSDs	-0.120	Weak	0.778

A weak correlation negative association between Total volume Pre Embol ChSDs and Angio-2 (vein) Patients was estimated.

Observed dependence of Total volume Pre Embol ChSDs from Angio-2 (vein) Patients is described by a linear regression equation:

$$Y_{\text{Total volume Pre Embol ChSDs}} = 0.01 \times X_{\text{Angio-2 (vein) Patients}} + 64.069$$

With an 1 pg/ml increase of Angio-2 (vein) Patients 0.01 ml change of Total volume Pre Embol ChSDs should be expected. According to the coefficient of determination R^2 of the resulting model, 10.3% of the observed variance of Total volume Pre Embol ChSDs were explained..

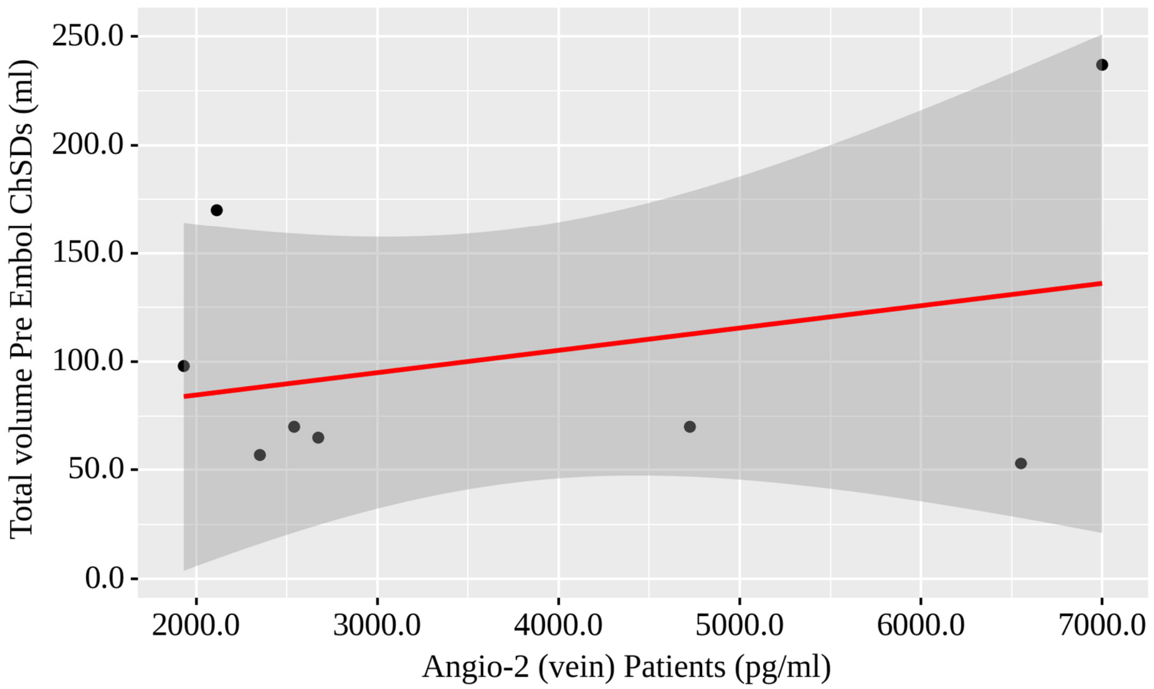

Figure 79 – Regression line characterizing the dependence of Total volume Pre Embol ChSDs from Angio-2 (vein) Patients

We performed analysis of Angio-2 (vein) Patients conditioning on Surgery.

Table 58 – Analysis of Angio-2 (vein) Patients conditioning on Surgery

Variable	Categories	Angio-2 (vein) Patients (pg/ml)			p
		Me	$Q_1 – Q_3$	n	
Surgery	none	2233	2068 – 3402	4	0.149
	operated	3700	2641 – 5294	4	

When comparing of Angio-2 (vein) Patients depending on Surgery no statistically significant differences were revealed (p = 0.149) *(applied method: Mann-Whitney U-test)*.

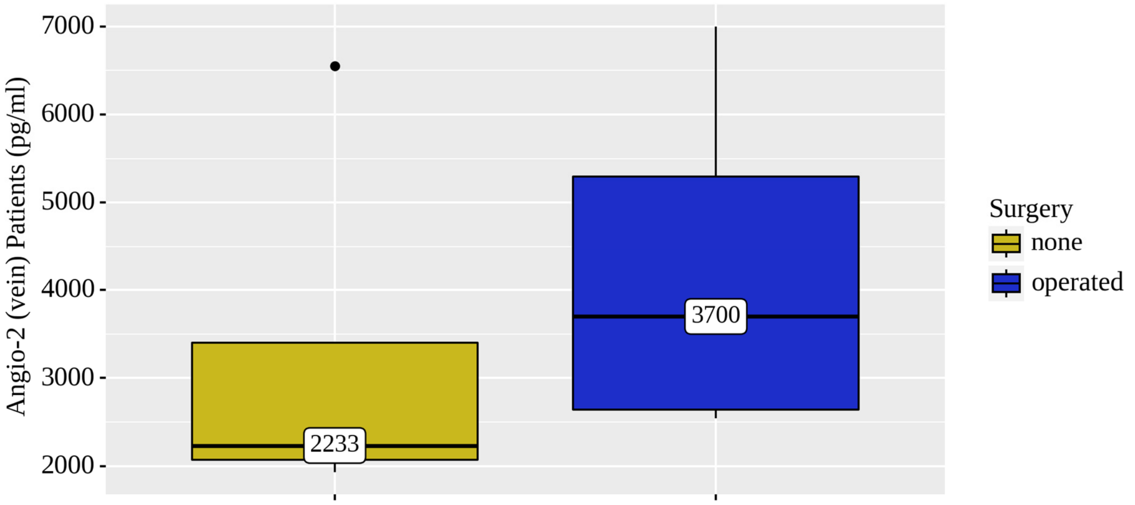

Figure 80 – Analysis of Angio-2 (vein) Patients conditioning on Surgery

When evaluating the dependence of the probability of operated on the Angio-2 (vein) Patients using the ROC analysis, the following curve was obtained.

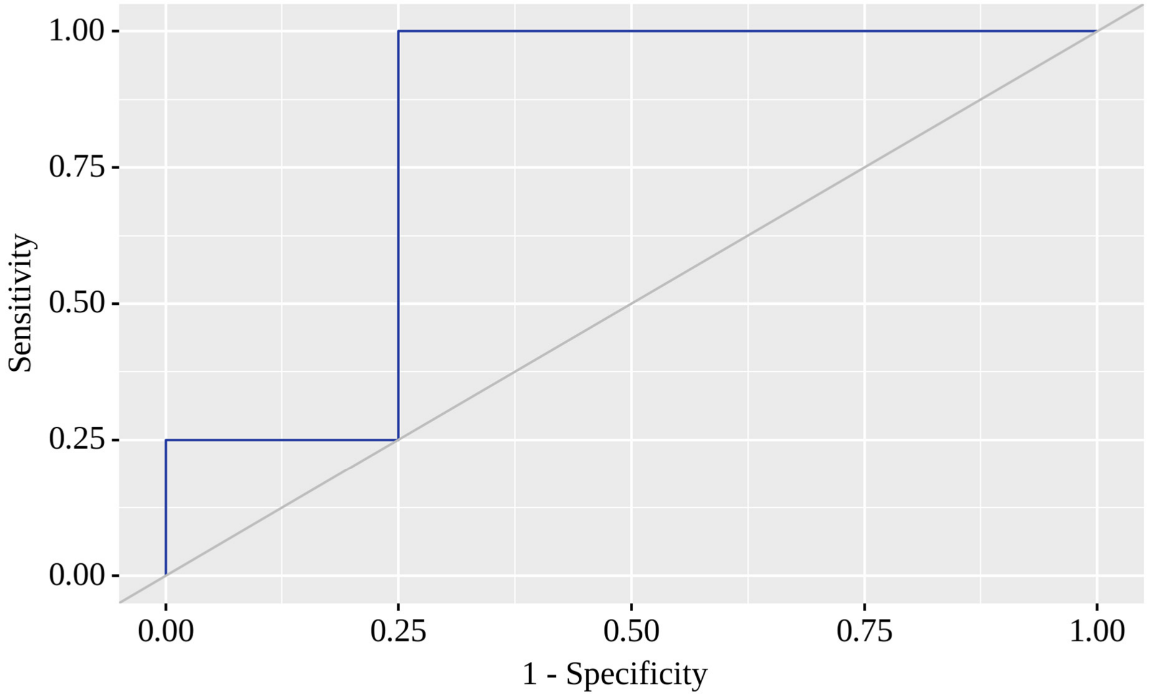

Figure 81 – ROC-curve characterizing the dependence of the probability Surgery on Angio-2 (vein) Patients

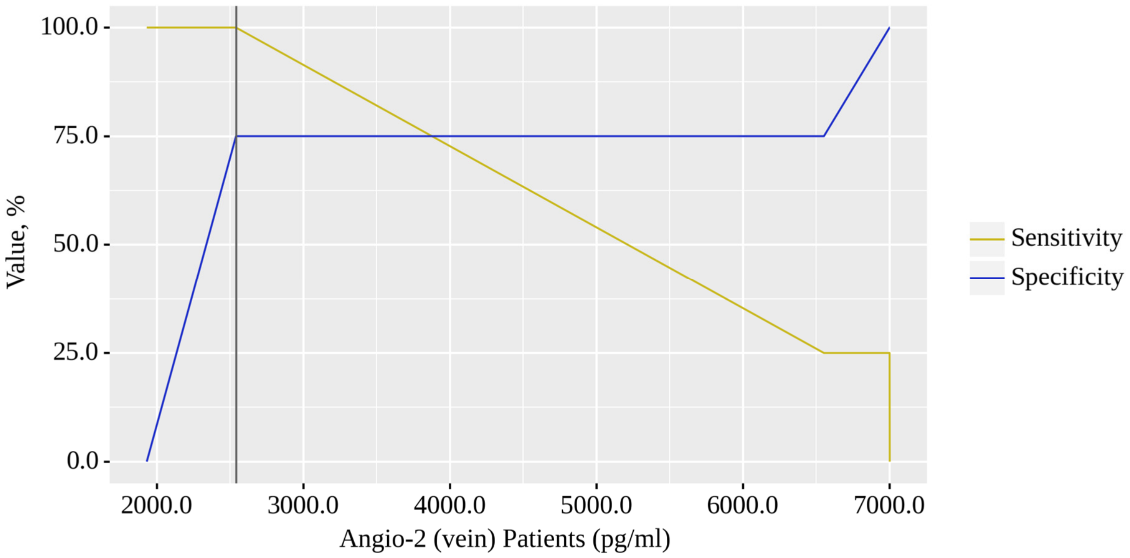

Figure 82 - Analysis of the sensitivity and specificity of Surgery depending on Angio-2 (vein) Patients

Table 59 – Threshold Angio-2 (vein) Patients

Threshold	Sensitivity (Se), %	Specificity (Sp), %	PPV	NPV
**2541**	**100.0**	**75.0**	**80.0**	**100.0**

The area under the ROC curve comprised 0.812 ± 0.164 with 95% CI: 0.491 - 1.000. The resulting model was not statistically significant (p = 0.149).

The cut-off value of Angio-2 (vein) Patients which corresponds to the highest Youden's J statistic is 2541.000 pg/ml. If Angio-2 (vein) Patients was greater than or equal to this value, operated was predicted. The sensitivity and specificity of the method were 100.0% and 75.0%, respectively.

Correlation analysis of the association between Angio-2 (artery) Patients and Angio-2 (vein) Patients was performed.

Table 60 – Results of the correlation analysis of the association between Angio-2 (artery) Patients and Angio-2 (vein) Patients

Variable	Correlation characteristics		
	r_{xy}	Strength of the association assesed using Chaddock scale	p
Angio-2 (artery) Patients – Angio-2 (vein) Patients	0.988	Functional	< 0.001*

* – differences are statistically significant ($p < 0.05$)

A functional correlation positive association between Angio-2 (vein) Patients and Angio-2 (artery) Patients was estimated.

Observed dependence of Angio-2 (vein) Patients from Angio-2 (artery) Patients is described by a linear regression equation:

$$Y_{\text{Angio-2 (vein) Patients}} = 0.914 \times X_{\text{Angio-2 (artery) Patients}} + 522.478$$

With an 1 pg/ml increase of Angio-2 (artery) Patients 0.914 pg/ml change of Angio-2 (vein) Patients should be expected. According to the coefficient of determination R^2 of the resulting model, 97.6% of the observed variance of Angio-2 (vein) Patients were explained..

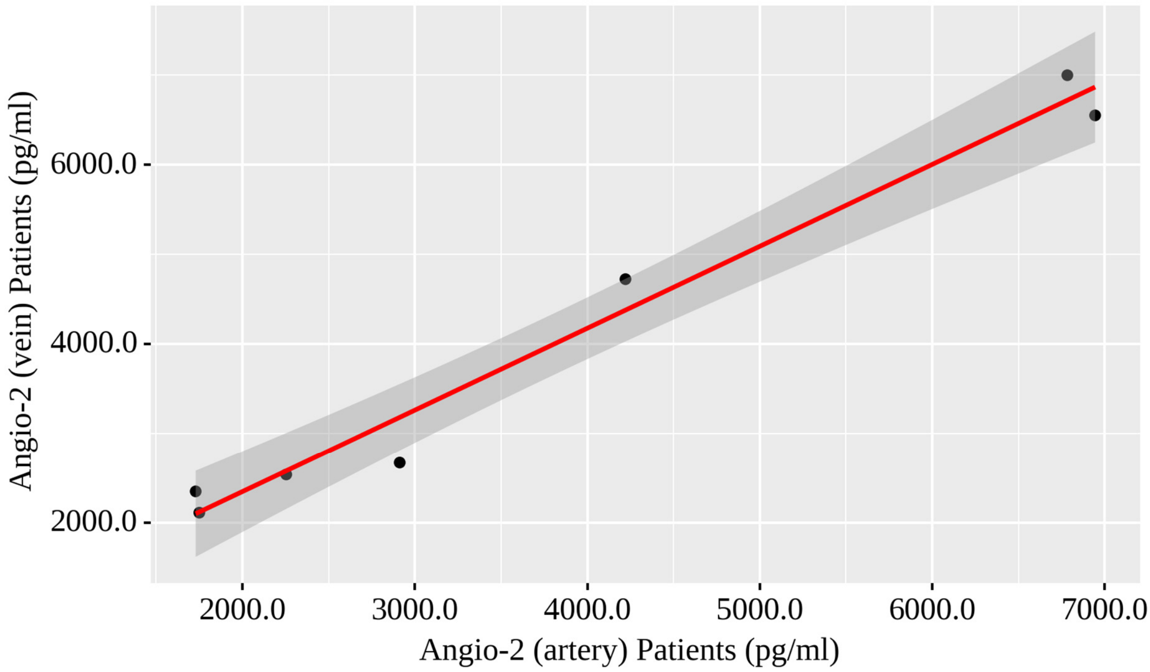

Figure 83 – Regression line characterizing the dependence of Angio-2 (vein) Patients from Angio-2 (artery) Patients

We performed analysis of Angio-2 (artery) Patients conditioning on Sides ChSDH.

Table 61 – Analysis of Angio-2 (artery) Patients conditioning on Sides ChSDH

Variable	Categories	Angio-2 (artery) Patients (pg/ml)			p
		Me	$Q_1 – Q_3$	n	
Sides ChSDH	Bilateral ChSDH	6783	6783 – 6783	1	0.317

	Monolateral ChSDH	2583	1876 – 3894	6	

When comparing of Angio-2 (artery) Patients depending on Sides ChSDH no statistically significant differences were revealed (p = 0.317) *(applied method: Mann-Whitney U-test)*.

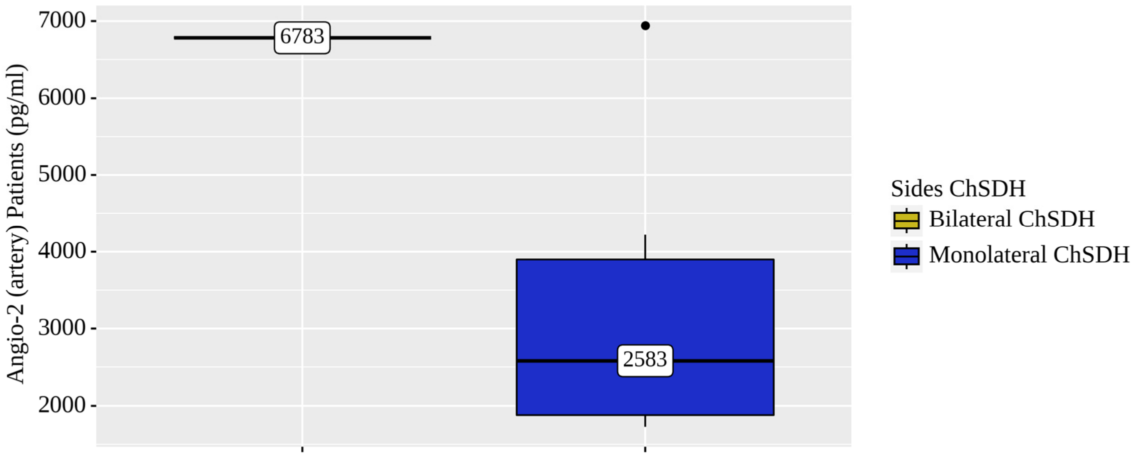

Figure 84 – Analysis of Angio-2 (artery) Patients conditioning on Sides ChSDH

When evaluating the dependence of the probability of Monolateral ChSDH on the Angio-2 (artery) Patients using the ROC analysis, the following curve was obtained.

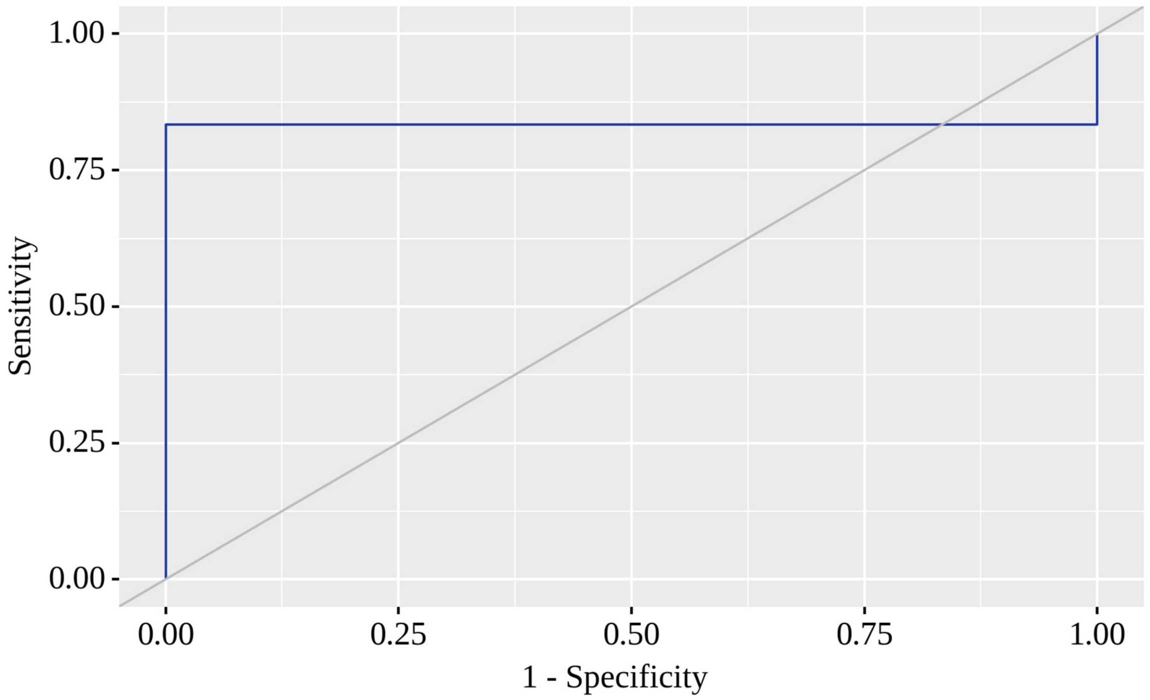

Figure 85 – ROC-curve characterizing the dependence of the probability Sides ChSDH on Angio-2 (artery) Patients

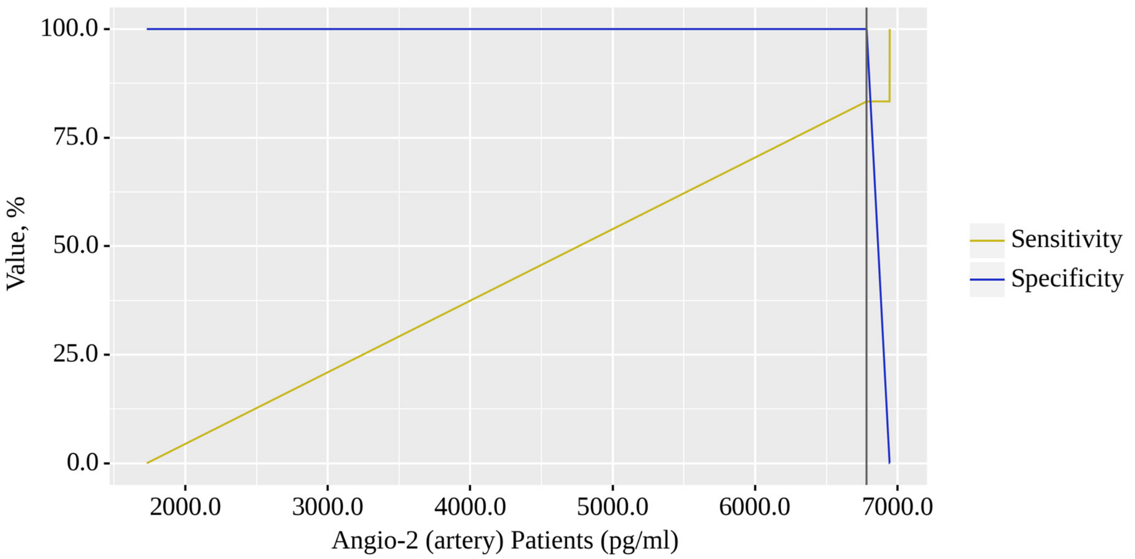

Figure 86 - Analysis of the sensitivity and specificity of Sides ChSDH depending on Angio-2 (artery) Patients

Table 62 – Threshold Angio-2 (artery) Patients

Threshold	Sensitivity (Se), %	Specificity (Sp), %	PPV	NPV
**6783**	**83.3**	**100.0**	**100.0**	**50.0**

The area under the ROC curve comprised 0.833 ± 0.275 with 95% CI: 0.294 - 1.000. The resulting model was not statistically significant (p = 0.317).

The cut-off value of Angio-2 (artery) Patients which corresponds to the highest Youden's J statistic is 6783.000 pg/ml. If Angio-2 (artery) Patients was less than this value, Monolateral ChSDH was predicted. The sensitivity and specificity of the method were 83.3% and 100.0%, respectively.

Correlation analysis of the association between Angio-2 (artery) Patients and Total volume Pre Embol ChSDs was performed.

Table 63 – Results of the correlation analysis of the association between Angio-2 (artery) Patients and Total volume Pre Embol ChSDs

Variable	Correlation characteristics		
	ρ	Strength of the association assesed using Chaddock scale	p
Angio-2 (artery) Patients – Total volume Pre Embol ChSDs	-0.036	None	0.939

There was no association between Total volume Pre Embol ChSDs and Angio-2 (artery) Patients.

Observed dependence of Total volume Pre Embol ChSDs from Angio-2 (artery) Patients is described by a linear regression equation:

$$Y_{\text{Total volume Pre Embol ChSDs}} = 0.009 \times X_{\text{Angio-2 (artery) Patients}} + 69.493$$

With an 1 pg/ml increase of Angio-2 (artery) Patients 0.009 ml change of Total volume Pre Embol ChSDs should be expected. According to the coefficient of determination R^2 of the resulting model, 7.8% of the observed variance of Total volume Pre Embol ChSDs were explained..

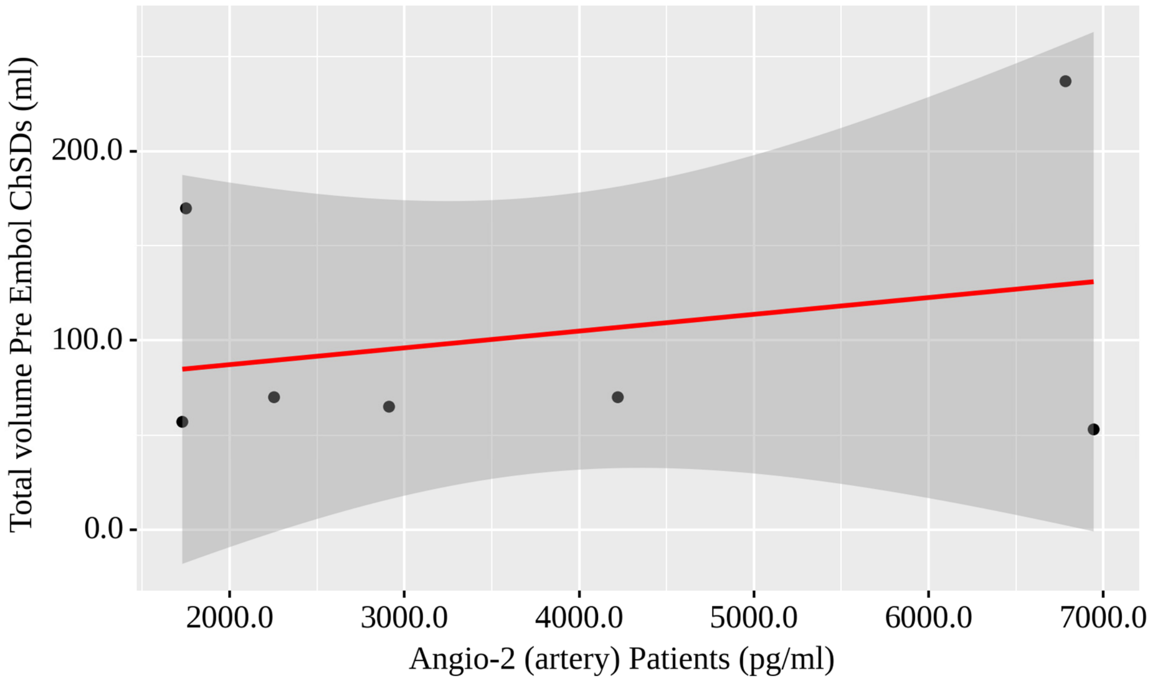

Figure 87 – Regression line characterizing the dependence of Total volume Pre Embol ChSDs from Angio-2 (artery) Patients

Analysis of Angio-2 (artery) Patients was performed conditioning on Rebleeding on CT scans.

Table 64 – Analysis of Angio-2 (artery) Patients conditioning on Rebleeding on CT scans

Variable	Categories	Angio-2 (artery) Patients (pg/ml)			p
		M ± SD	95% CI	n	
Rebleeding on CT scans	none	4037 ± 2539	-2271 – 10344	3	0.834
	rebleeded	3621 ± 2411	-216 – 7457	4	

When comparing of Angio-2 (artery) Patients depending on Rebleeding on CT scans there were no statistically significant differences (p = 0.834) *(applied method: Student's t-test)*.

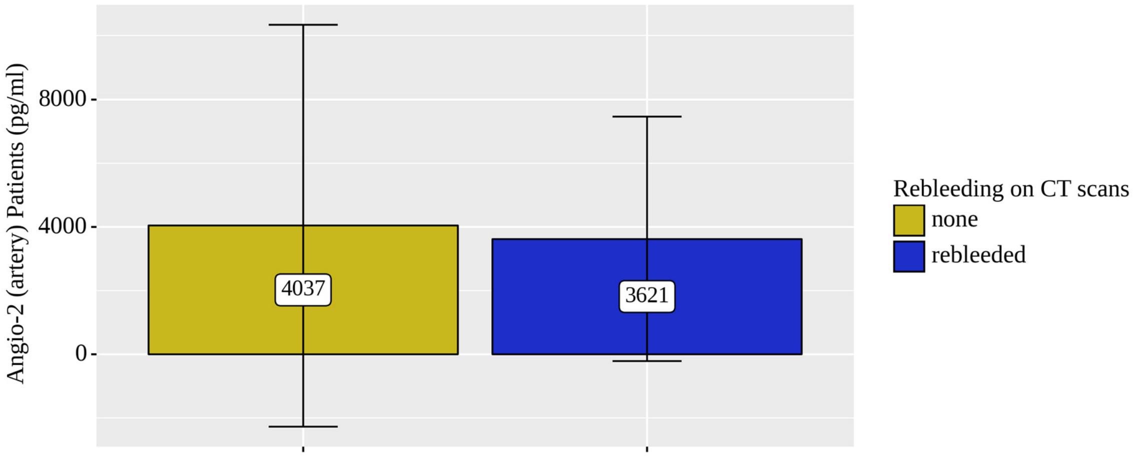

Figure 88 – Analysis of Angio-2 (artery) Patients conditioning on Rebleeding on CT scans

When evaluating the dependence of the probability of rebleeded on the Angio-2 (artery) Patients using the ROC analysis, the following curve was obtained.

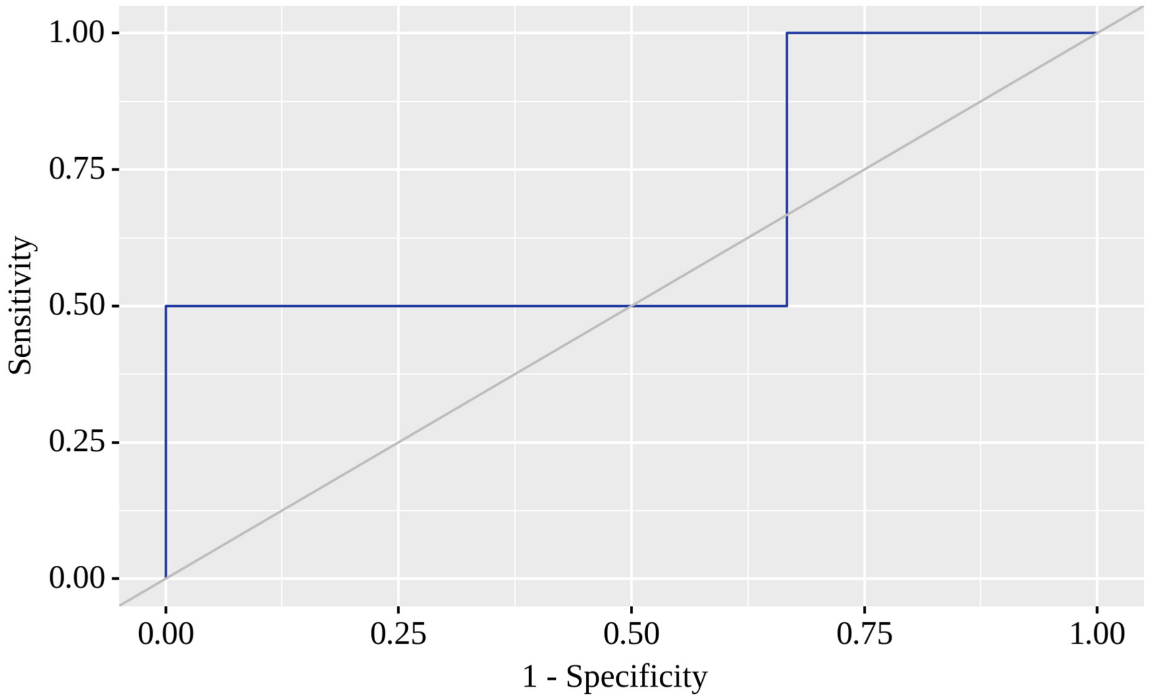

Figure 89 – ROC-curve characterizing the dependence of the probability Rebleeding on CT scans on Angio-2 (artery) Patients

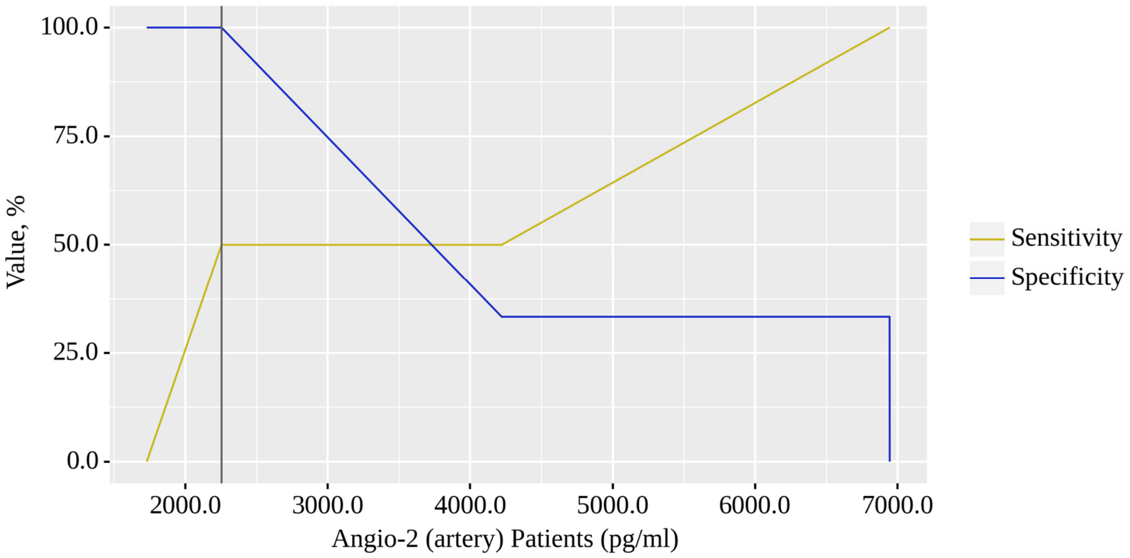

Figure 90 - Analysis of the sensitivity and specificity of Rebleeding on CT scans depending on Angio-2 (artery) Patients

Table 65 – Threshold Angio-2 (artery) Patients

Threshold	Sensitivity (Se), %	Specificity (Sp), %	PPV	NPV
**2254**	**50.0**	**100.0**	**100.0**	**60.0**

The area under the ROC curve comprised 0.667 ± 0.224 with 95% CI: 0.228 - 1.000. The resulting model was not statistically significant ($p = 0.480$).

The cut-off value of Angio-2 (artery) Patients which corresponds to the highest Youden's J statistic is 2254.000 pg/ml. If Angio-2 (artery) Patients was less than this value, rebleeded was predicted. The sensitivity and specificity of the method were 50.0% and 100.0%, respectively.

Analysis of Angio-2 (artery) Patients was performed conditioning on Surgery.

Table 66 – Analysis of Angio-2 (artery) Patients conditioning on Surgery

Variable	Categories	Angio-2 (artery) Patients (pg/ml)			p
		Me	$Q_1 – Q_3$	n	
Surgery	none	1750	1740 – 4347	3	0.480
	operated	3566	2748 – 4862	4	

When comparing of Angio-2 (artery) Patients depending on Surgery there were no statistically significant differences (p = 0.480) *(applied method: Mann-Whitney U-test)*.

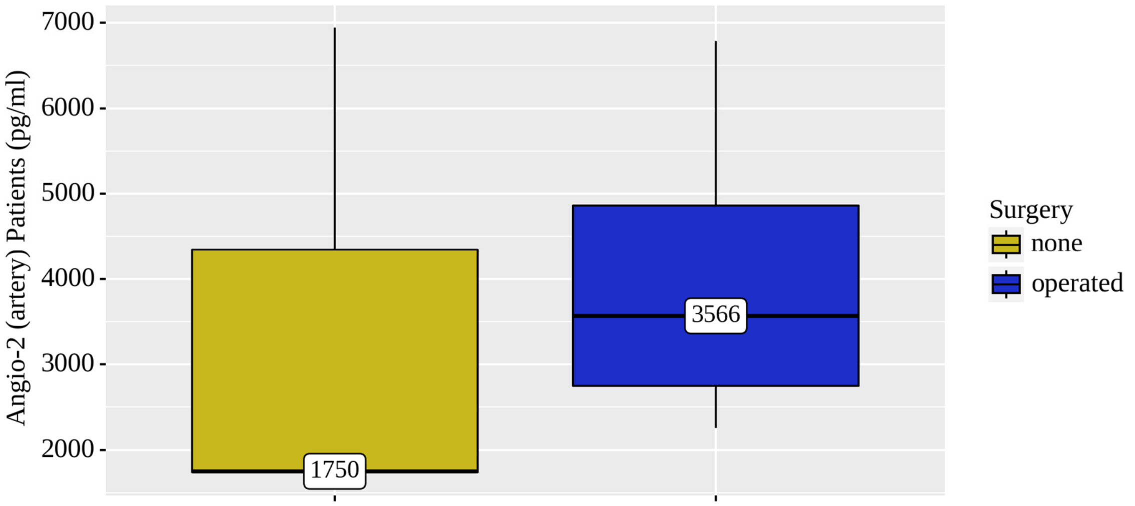

Figure 91 – Analysis of Angio-2 (artery) Patients conditioning on Surgery

When evaluating the dependence of the probability of operated on the Angio-2 (artery) Patients using the ROC analysis, the following curve was obtained.

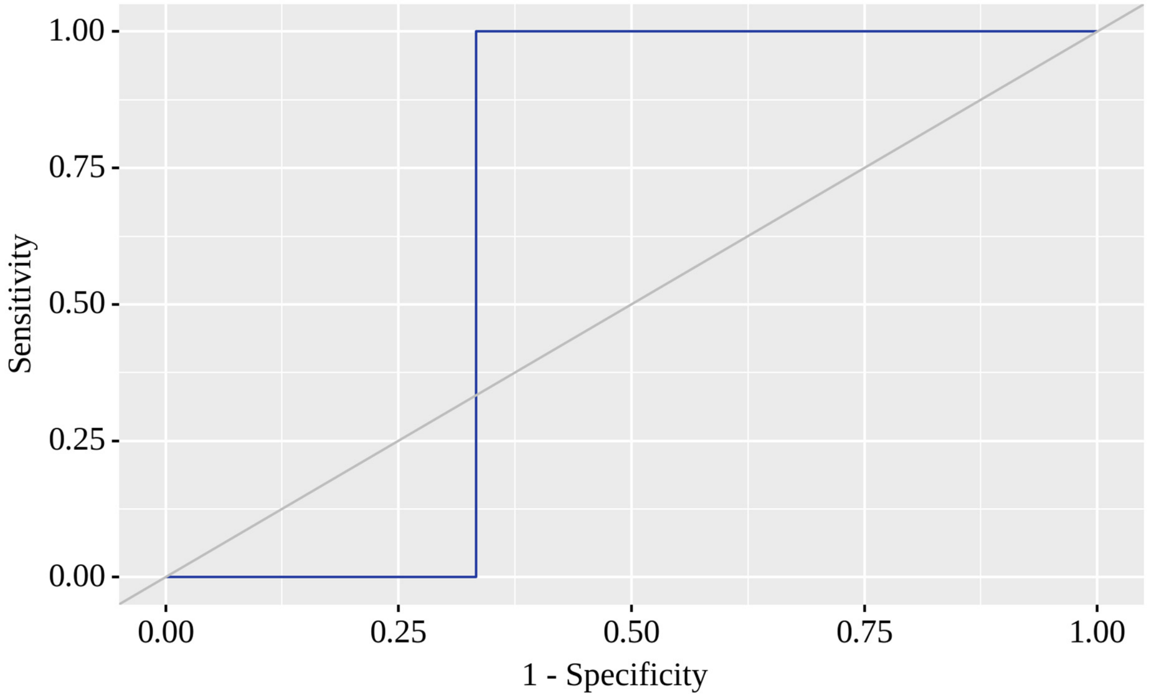

Figure 92 – ROC-curve characterizing the dependence of the probability Surgery on Angio-2 (artery) Patients

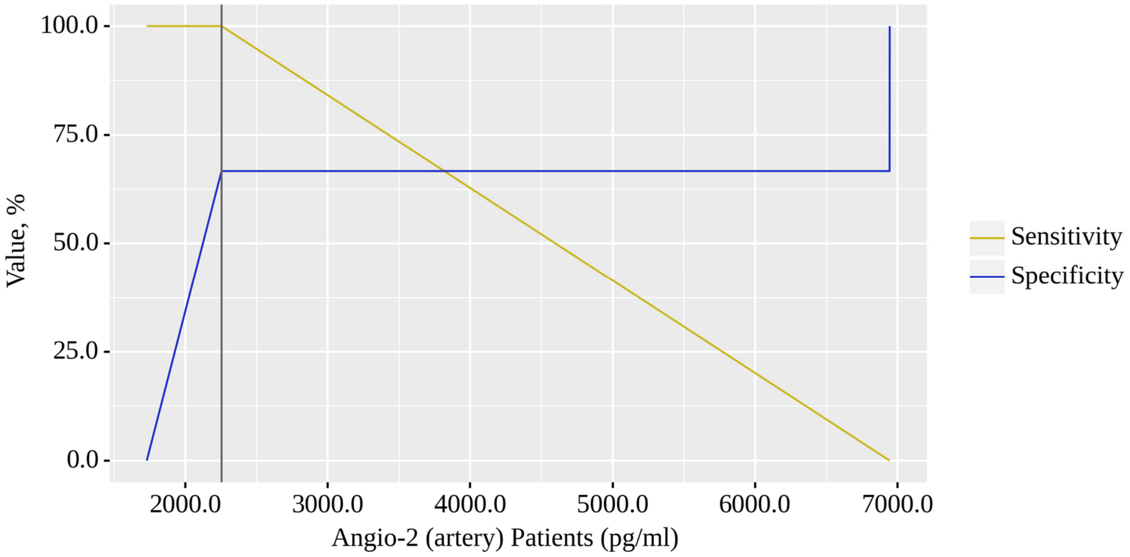

Figure 93 - Analysis of the sensitivity and specificity of Surgery depending on Angio-2 (artery) Patients

Table 67 – Threshold Angio-2 (artery) Patients

Threshold	Sensitivity (Se), %	Specificity (Sp), %	PPV	NPV
**2254**	**100.0**	**66.7**	**80.0**	**100.0**

The area under the ROC curve comprised 0.667 ± 0.217 with 95% CI: 0.241 - 1.000. The resulting model was not statistically significant ($p = 0.480$).

The cut-off value of Angio-2 (artery) Patients which corresponds to the highest Youden's J statistic is 2254.000 pg/ml. If Angio-2 (artery) Patients was greater than or equal to this value, operated was predicted. The sensitivity and specificity of the method were 100.0% and 66.7%, respectively.

We performed analysis of TGF-β1 (vein) P/HV conditioning on Patients/Healthy volunteers.

Table 68 – Analysis of TGF-β1 (vein) P/HV conditioning on Patients/Healthy volunteers

Variable	Categories	TGF-β1 (vein) P/HV (pg/ml)			p
		M ± SD	95% CI	n	
Patients/Healthy volunteers	patient	7462 ± 3497	4539 – 10386	8	0.026*
	healthy volunteer	10936 ± 411	10748 – 11123	21	

* – differences are statistically significant ($p < 0.05$)

According to the data obtained when comparing of TGF-β1 (vein) P/HV statistically significant differences were revealed depending on Patients/Healthy volunteers (p = 0.026) *(applied method: Welch's t-test)*.

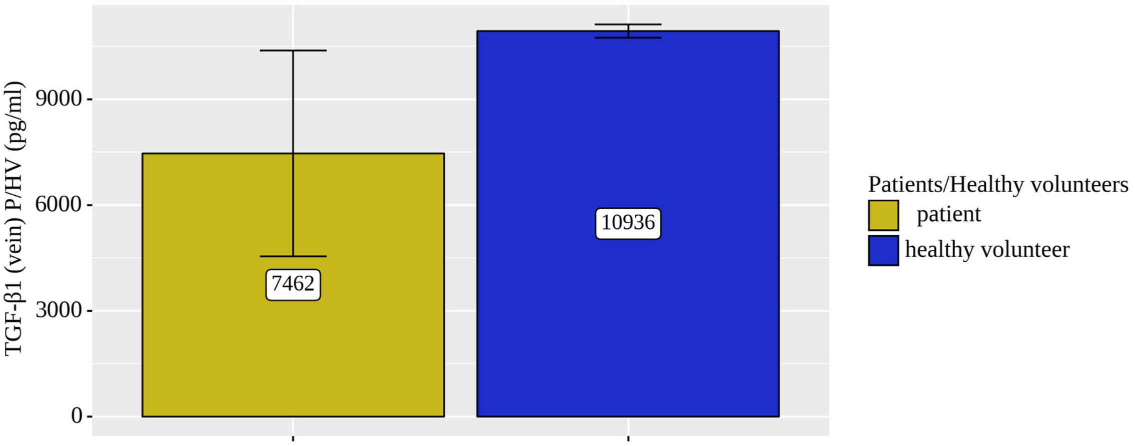

Figure 94 – Analysis of TGF-β1 (vein) P/HV conditioning on Patients/Healthy volunteers

When evaluating the dependence of the probability of healthy volunteer on the TGF-β1 (vein) P/HV using the ROC analysis, the following curve was obtained.

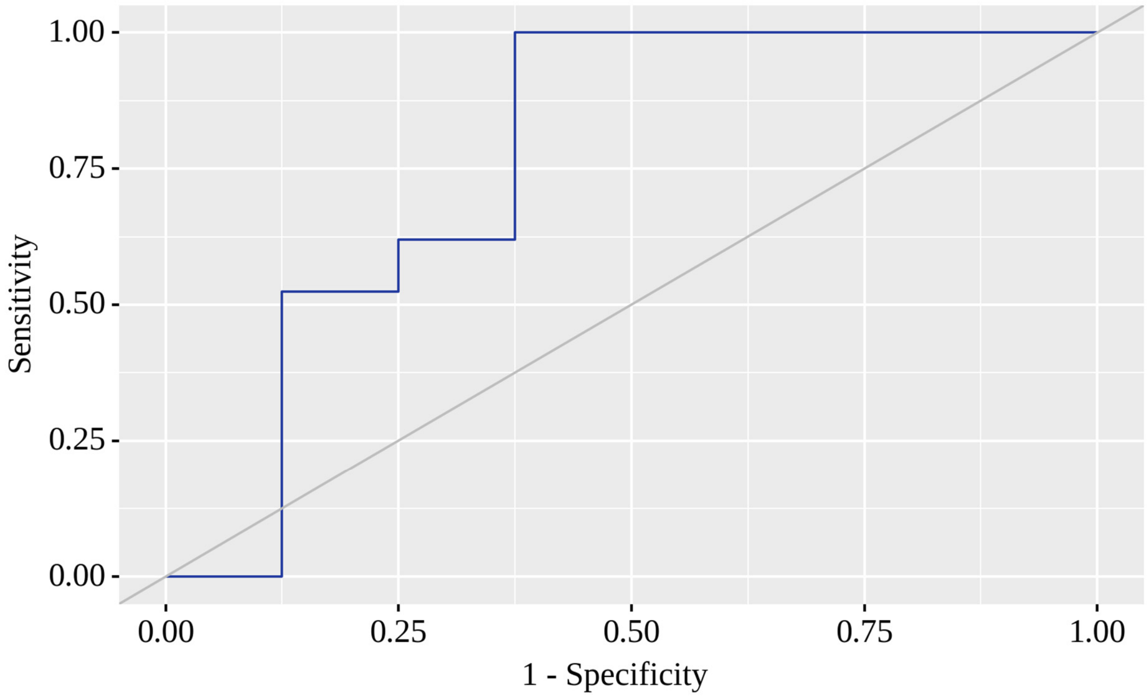

Figure 95 – ROC-curve characterizing the dependence of the probability Patients/Healthy volunteers on TGF-β1 (vein) P/HV

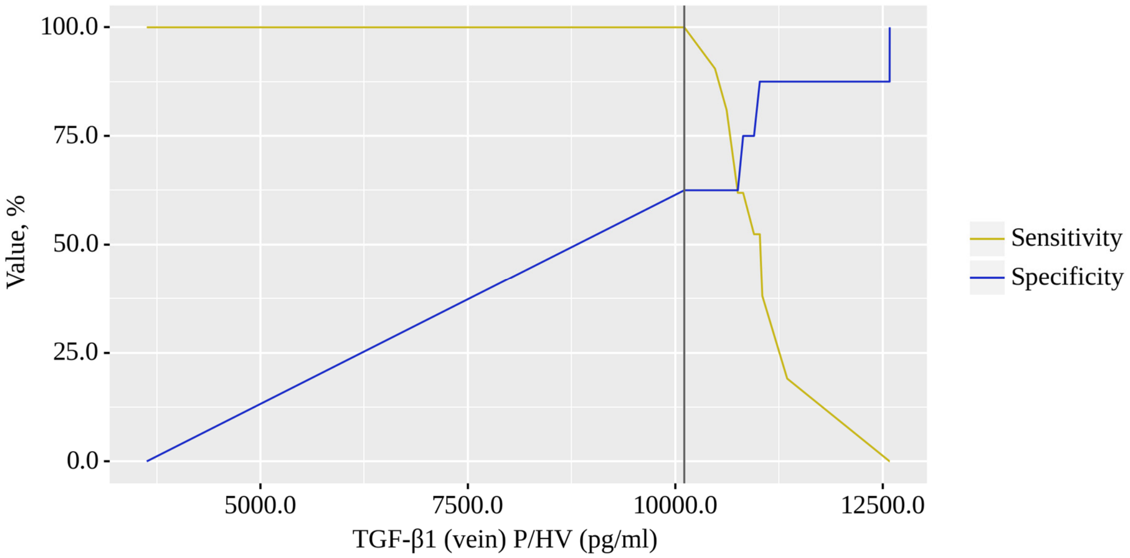

Figure 96 - Analysis of the sensitivity and specificity of Patients/Healthy volunteers depending on TGF-β1 (vein) P/HV

Table 69 – Threshold TGF-β1 (vein) P/HV

Threshold	Sensitivity (Se), %	Specificity (Sp), %	PPV	NPV
11020	52.4	87.5	91.7	41.2
10950	52.4	75.0	84.6	37.5
10819	61.9	75.0	86.7	42.9
**10755**	**61.9**	**62.5**	**81.2**	**38.5**
10620	81.0	62.5	85.0	55.6
10480	90.5	62.5	86.4	71.4
10110	100.0	62.5	87.5	100.0

The area under the ROC curve comprised 0.768 ± 0.091 with 95% CI: 0.589 - 0.946. The resulting model was statistically significant ($p = 0.028$).

The cut-off value of TGF-β1 (vein) P/HV which corresponds to the highest Youden's J statistic is 10110.000 pg/ml. If TGF-β1 (vein) P/HV was greater than or equal to this value, healthy volunteer was predicted. The sensitivity and specificity of the method were 100.0% and 62.5%, respectively.

Analysis of TGF-β1 (vein) Patients was performed conditioning on Sides ChSDH.

Table 70 – Analysis of TGF-β1 (vein) Patients conditioning on Sides ChSDH

Variable	Categories	TGF-β1 (vein) Patients (pg/ml)	p

		Me	$Q_1 - Q_3$	n	
Sides ChSDH	Bilateral ChSDH	8895	7965 – 9825	2	0.505
	Monolateral ChSDH	5378	4185 – 9709	6	

When comparing of TGF-β1 (vein) Patients depending on Sides ChSDH no statistically significant differences were revealed ($p = 0.505$) *(applied method: Mann-Whitney U-test)*.

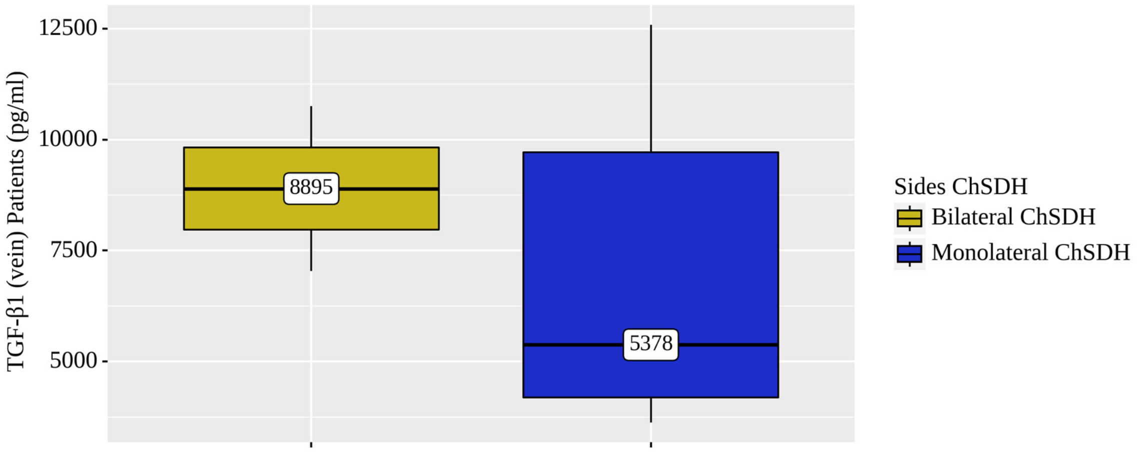

Figure 97 – Analysis of TGF-β1 (vein) Patients conditioning on Sides ChSDH

When evaluating the dependence of the probability of Monolateral ChSDH on the TGF-β1 (vein) Patients using the ROC analysis, the following curve was obtained.

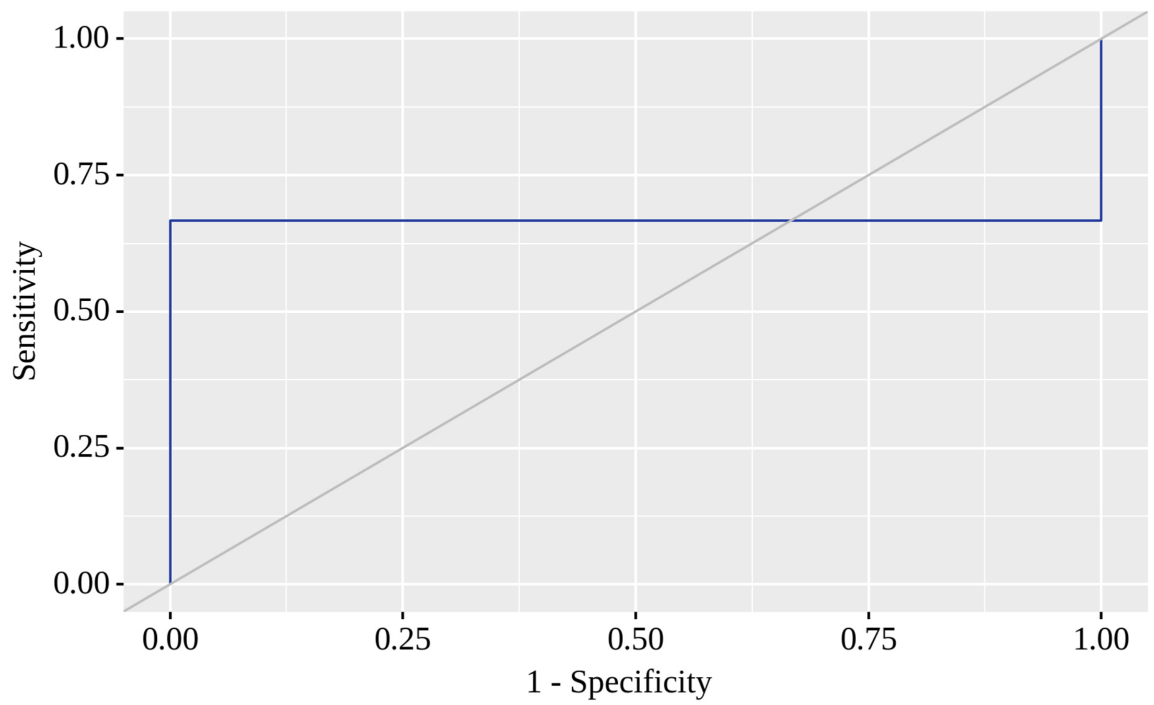

Figure 98 – ROC-curve characterizing the dependence of the probability Sides ChSDH on TGF-β1 (vein) Patients

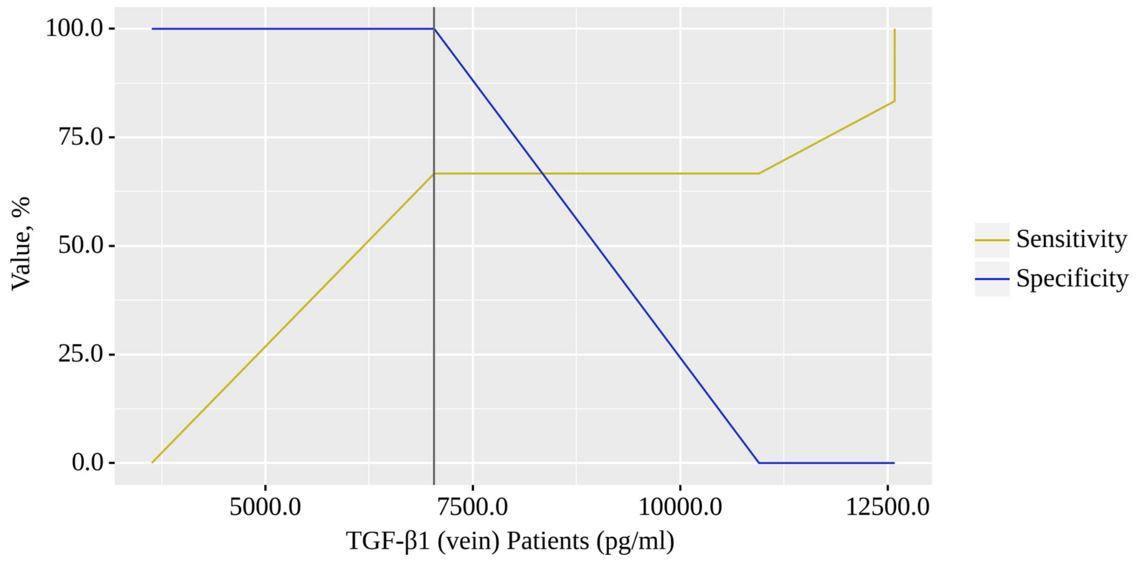

Figure 99 - Analysis of the sensitivity and specificity of Sides ChSDH depending on TGF-β1 (vein) Patients

Table 71 – Threshold TGF-β1 (vein) Patients

Threshold	Sensitivity (Se), %	Specificity (Sp), %	PPV	NPV
**7035**	**66.7**	**100.0**	**100.0**	**50.0**

The area under the ROC curve comprised 0.667 ± 0.245 with 95% CI: 0.186 - 1.000. The resulting model was not statistically significant (p = 0.505).

The cut-off value of TGF-β1 (vein) Patients which corresponds to the highest Youden's J statistic is 7035.000 pg/ml. If TGF-β1 (vein) Patients was less than this value, Monolateral ChSDH was predicted. The sensitivity and specificity of the method were 66.7% and 100.0%, respectively.

We performed analysis of TGF-β1 (vein) Patients conditioning on Rebleeding on CT scans.

Table 72 – Analysis of TGF-β1 (vein) Patients conditioning on Rebleeding on CT scans

Variable	Categories	TGF-β1 (vein) Patients (pg/ml)			p
		M ± SD	95% CI	n	
Rebleeding on CT scans	none	6900 ± 3052	2043 – 11757	4	0.684
	rebleeded	8025 ± 4287	1204 – 14846	4	

When comparing of TGF-β1 (vein) Patients depending on Rebleeding on CT scans no statistically significant differences were revealed (p = 0.684) *(applied method: Student's t-test)*.

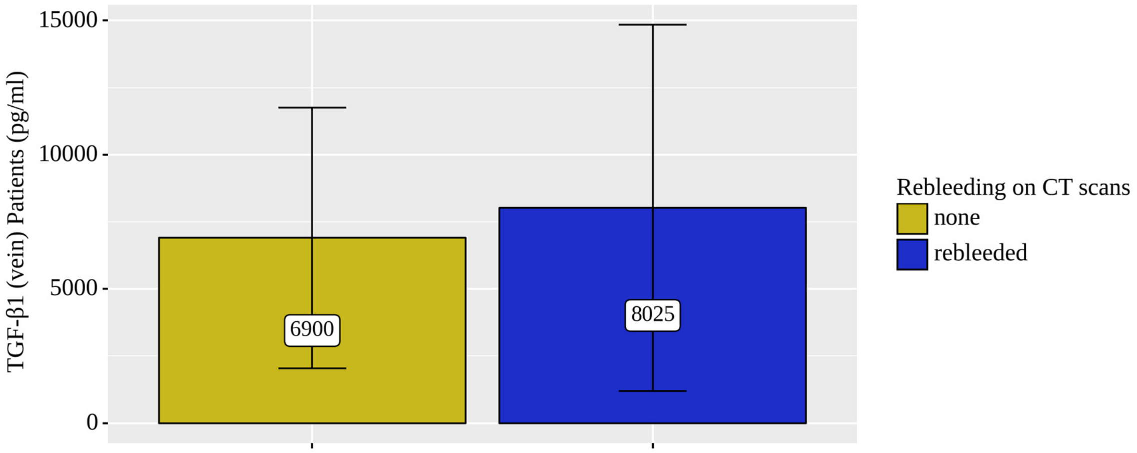

Figure 100 – Analysis of TGF-β1 (vein) Patients conditioning on Rebleeding on CT scans

When evaluating the dependence of the probability of rebleeded on the TGF-β1 (vein) Patients using the ROC analysis, the following curve was obtained.

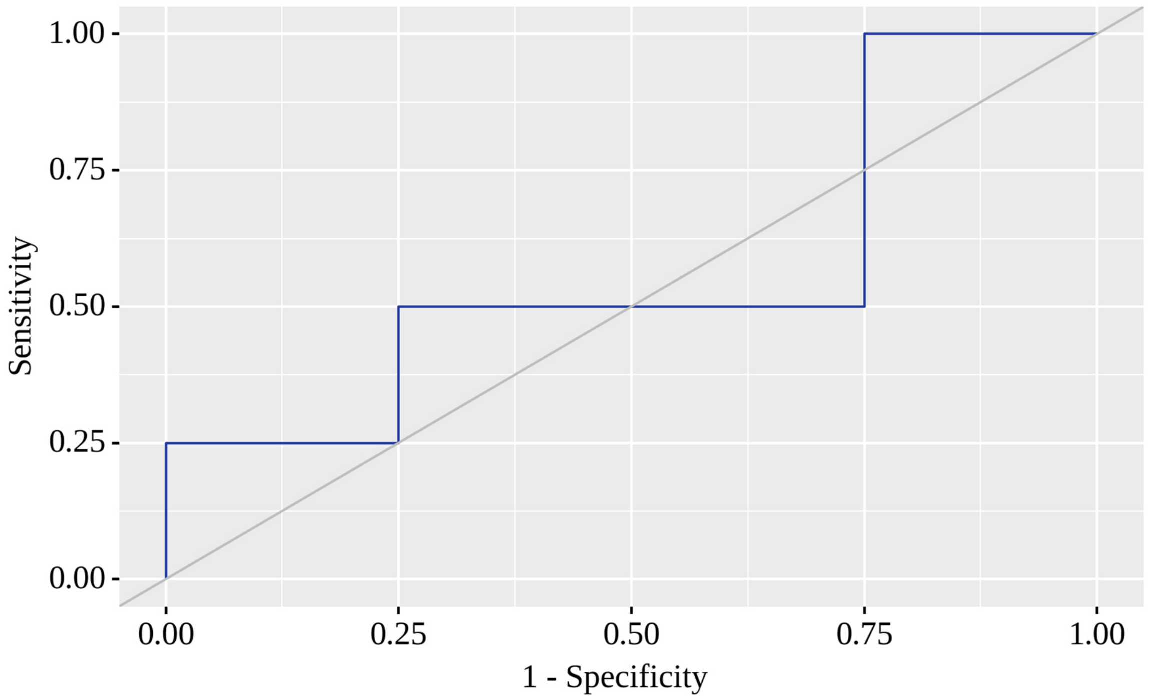

Figure 101 – ROC-curve characterizing the dependence of the probability Rebleeding on CT scans on TGF-β1 (vein) Patients

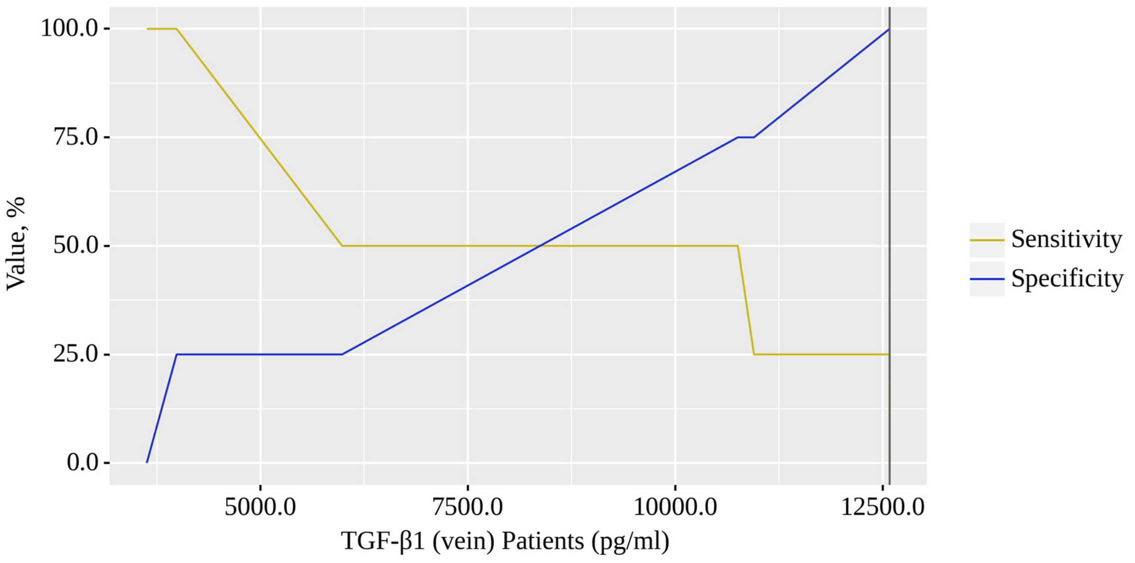

Figure 102 - Analysis of the sensitivity and specificity of Rebleeding on CT scans depending on TGF-β1 (vein) Patients

Table 73 – Threshold TGF-β1 (vein) Patients

Threshold	Sensitivity (Se), %	Specificity (Sp), %	PPV	NPV

**10755**	**50.0**	**75.0**	**66.7**	**60.0**

The area under the ROC curve comprised 0.562 ± 0.215 with 95% CI: 0.142 - 0.983. The resulting model was not statistically significant ($p = 0.773$).

The cut-off value of TGF-β1 (vein) Patients which corresponds to the highest Youden's J statistic is 12585.000 pg/ml. If TGF-β1 (vein) Patients was greater than or equal to this value, rebleeded was predicted. The sensitivity and specificity of the method were 25.0% and 100.0%, respectively.

We performed a correlation analysis of the association between TGF-β1 (vein) Patients and Total volume Pre Embol ChSDs.

Table 74 – Results of the correlation analysis of the association between TGF-β1 (vein) Patients and Total volume Pre Embol ChSDs

Variable	Correlation characteristics		
	ρ	Strength of the association assesed using Chaddock scale	p
TGF-β1 (vein) Patients – Total volume Pre Embol ChSDs	0.455	Moderate	0.257

A moderate correlation positive association between Total volume Pre Embol ChSDs and TGF-β1 (vein) Patients was estimated.

Observed dependence of Total volume Pre Embol ChSDs from TGF-β1 (vein) Patients is described by a linear regression equation:

$$Y_{\text{Total volume Pre Embol ChSDs}} = 0.013 \times X_{\text{TGF-β1 (vein) Patients}} + 6.352$$

With an 1 pg/ml increase of TGF-β1 (vein) Patients 0.013 ml change of Total volume Pre Embol ChSDs should be expected. According to the coefficient of determination R^2 of the resulting model, 46.3% of the observed variance of Total volume Pre Embol ChSDs were explained..

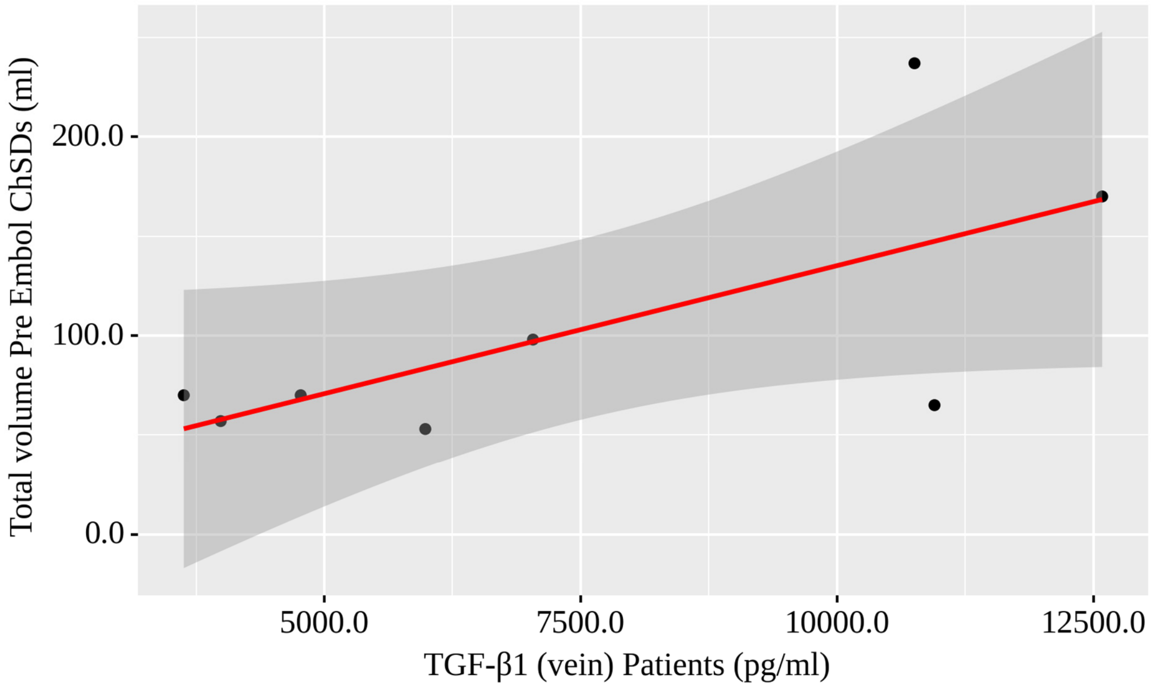

Figure 103 – Regression line characterizing the dependence of Total volume Pre Embol ChSDs from TGF-β1 (vein) Patients

We performed analysis of TGF-β1 (vein) Patients conditioning on Surgery.

Table 75 – Analysis of TGF-β1 (vein) Patients conditioning on Surgery

Variable	Categories	TGF-β1 (vein) Patients (pg/ml)			p
		M ± SD	95% CI	n	
Surgery	none	7399 ± 3681	1542 – 13256	4	0.963
	operated	7526 ± 3870	1369 – 13684	4	

When comparing of TGF-β1 (vein) Patients depending on Surgery there were no statistically significant differences ($p = 0.963$) *(applied method: Student's t-test)*.

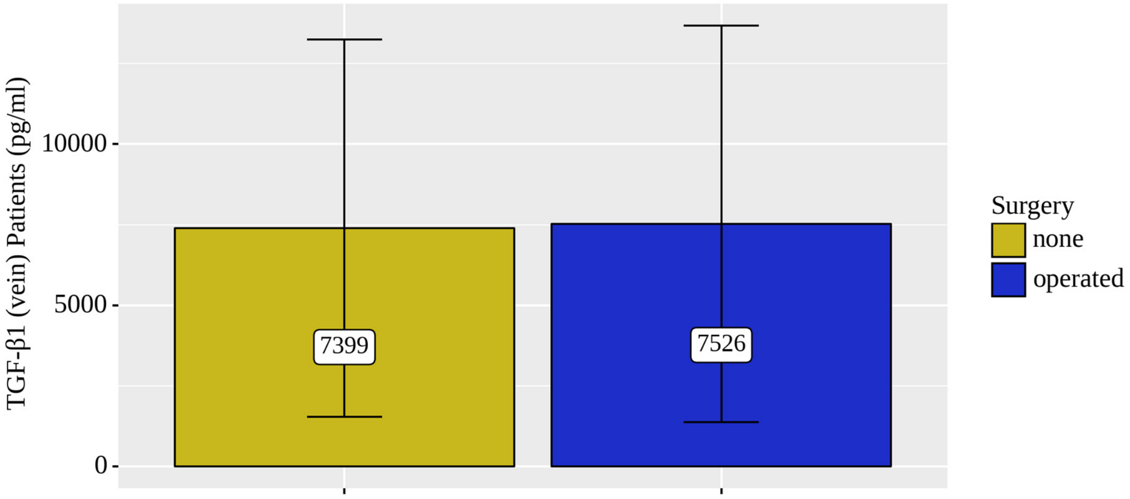

Figure 104 – Analysis of TGF-β1 (vein) Patients conditioning on Surgery

When evaluating the dependence of the probability of operated on the TGF-β1 (vein) Patients using the ROC analysis, the following curve was obtained.

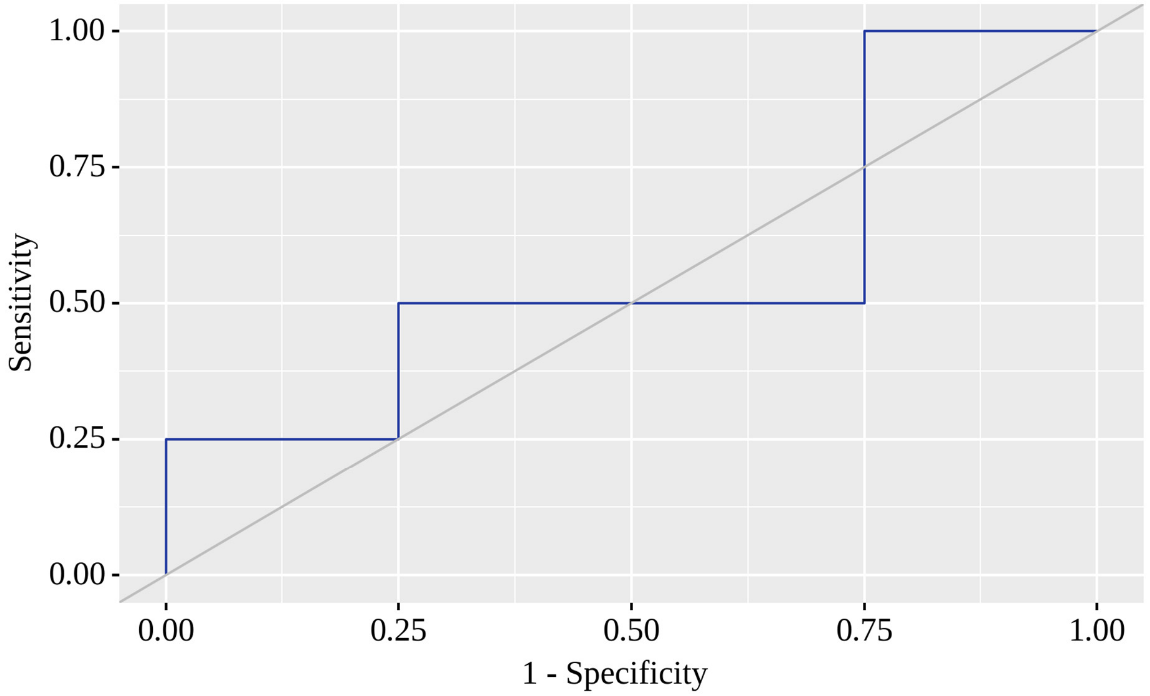

Figure 105 – ROC-curve characterizing the dependence of the probability Surgery on TGF-β1 (vein) Patients

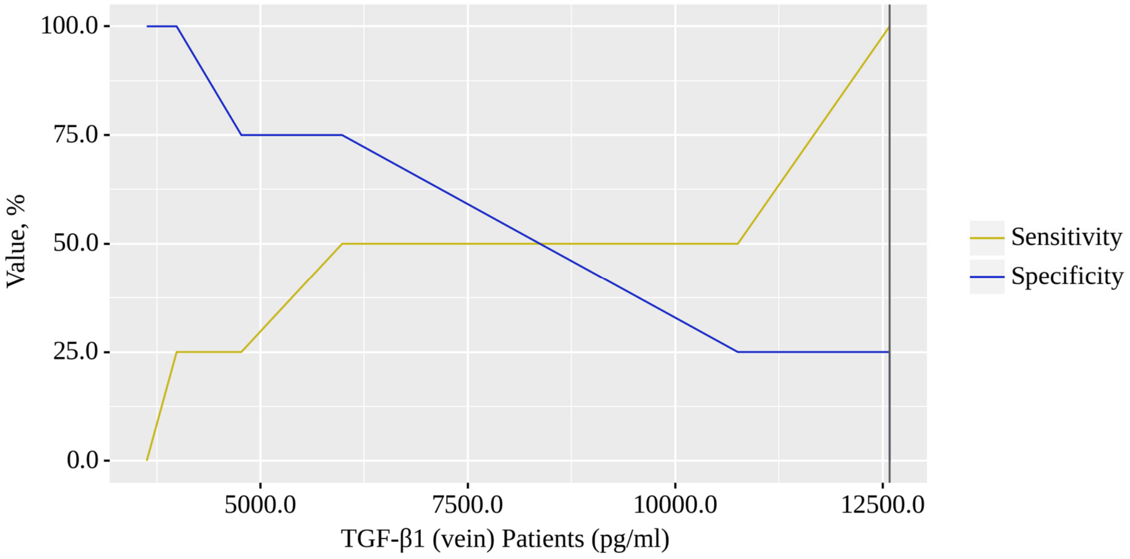

Figure 106 - Analysis of the sensitivity and specificity of Surgery depending on TGF-β1 (vein) Patients

Table 76 – Threshold TGF-β1 (vein) Patients

Threshold	Sensitivity (Se), %	Specificity (Sp), %	PPV	NPV
**5985**	**50.0**	**75.0**	**66.7**	**60.0**

The area under the ROC curve comprised 0.562 ± 0.215 with 95% CI: 0.142 - 0.983. The resulting model was not statistically significant (p = 0.773).

The cut-off value of TGF-β1 (vein) Patients which corresponds to the highest Youden's J statistic is 12585.000 pg/ml. If TGF-β1 (vein) Patients was less than this value, operated was predicted. The sensitivity and specificity of the method were 100.0% and 25.0%, respectively.

Correlation analysis of the association between TGF-β1 (artery) Patients and TGF-β1 (vein) Patients was performed.

Table 77 – Results of the correlation analysis of the association between TGF-β1 (artery) Patients and TGF-β1 (vein) Patients

Variable	Correlation characteristics		
	r_{xy}	Strength of the association assesed using Chaddock scale	p
TGF-β1 (artery) Patients – TGF-β1 (vein) Patients	0.624	Close	0.135

A close correlation positive association between TGF-β1 (vein) Patients and TGF-β1 (artery) Patients was estimated.

Observed dependence of TGF-β1 (vein) Patients from TGF-β1 (artery) Patients is described by a linear regression equation:

$$Y_{\text{TGF-β1 (vein) Patients}} = 1.773 \times X_{\text{TGF-β1 (artery) Patients}} - 8200.276$$

With an 1 pg/ml increase of TGF-β1 (artery) Patients 1.773 pg/ml change of TGF-β1 (vein) Patients should be expected. According to the coefficient of determination R^2 of the resulting model, 38.9% of the observed variance of TGF-β1 (vein) Patients were explained..

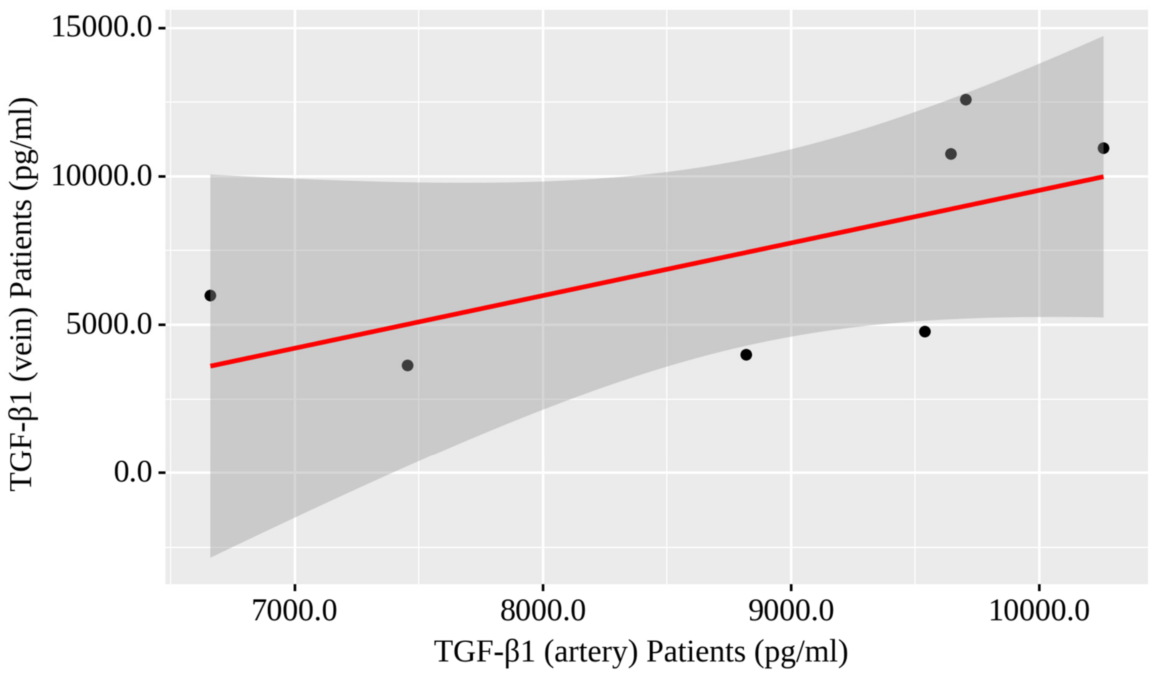

Figure 107 – Regression line characterizing the dependence of TGF-β1 (vein) Patients from TGF-β1 (artery) Patients

Analysis of TGF-β1 (artery) Patients was performed conditioning on Sides ChSDH.

Table 78 – Analysis of TGF-β1 (artery) Patients conditioning on Sides ChSDH

Variable	Categories	TGF-β1 (artery) Patients (pg/ml)			p
		Me	$Q_1 – Q_3$	n	
Sides ChSDH	Bilateral ChSDH	9645	9645 – 9645	1	0.617

	Monolateral ChSDH	9180	7796 – 9664	6	

When comparing of TGF-β1 (artery) Patients depending on Sides ChSDH no statistically significant differences were revealed (p = 0.617) *(applied method: Mann-Whitney U-test)*.

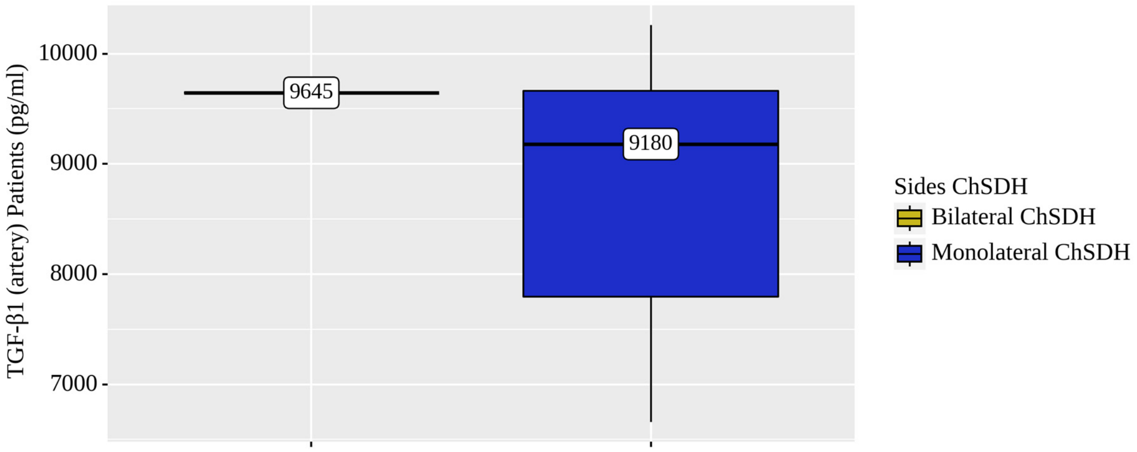

Figure 108 – Analysis of TGF-β1 (artery) Patients conditioning on Sides ChSDH

When evaluating the dependence of the probability of Monolateral ChSDH on the TGF-β1 (artery) Patients using the ROC analysis, the following curve was obtained.

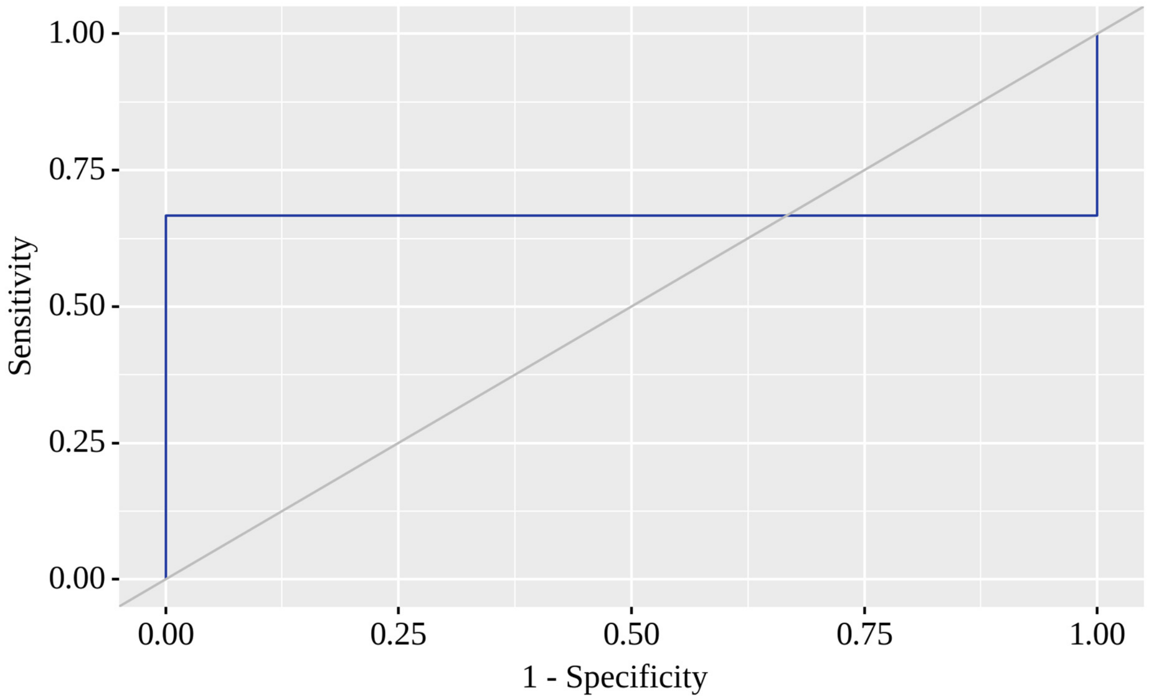

Figure 109 – ROC-curve characterizing the dependence of the probability Sides ChSDH on TGF-β1 (artery) Patients

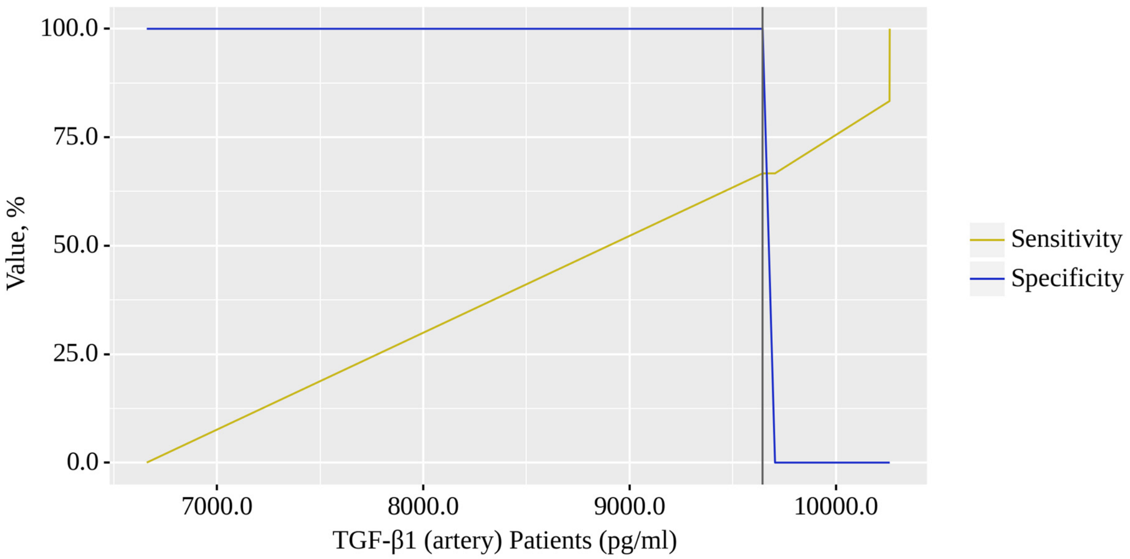

Figure 110 - Analysis of the sensitivity and specificity of Sides ChSDH depending on TGF-β1 (artery) Patients

Table 79 – Threshold TGF-β1 (artery) Patients

Threshold	Sensitivity (Se), %	Specificity (Sp), %	PPV	NPV
**9645**	**66.7**	**100.0**	**100.0**	**33.3**

The area under the ROC curve comprised 0.667 ± 0.333 with 95% CI: 0.013 - 1.000. The resulting model was not statistically significant (p = 0.617).

The cut-off value of TGF-β1 (artery) Patients which corresponds to the highest Youden's J statistic is 9645.000 pg/ml. If TGF-β1 (artery) Patients was less than this value, Monolateral ChSDH was predicted. The sensitivity and specificity of the method were 66.7% and 100.0%, respectively.

We performed a correlation analysis of the association between TGF-β1 (artery) Patients and Total volume Pre Embol ChSDs.

Table 80 – Results of the correlation analysis of the association between TGF-β1 (artery) Patients and Total volume Pre Embol ChSDs

Variable	Correlation characteristics		
	ρ	Strength of the association assesed using Chaddock scale	p
TGF-β1 (artery) Patients – Total volume Pre Embol ChSDs	0.505	Close	0.248

A close correlation positive association between Total volume Pre Embol ChSDs and TGF-β1 (artery) Patients was estimated.

Observed dependence of Total volume Pre Embol ChSDs from TGF-β1 (artery) Patients is described by a linear regression equation:

$$Y_{\text{Total volume Pre Embol ChSDs}} = 0.023 \times X_{\text{TGF-}\beta\text{1 (artery) Patients}} - 101.945$$

With an 1 pg/ml increase of TGF-β1 (artery) Patients 0.023 ml change of Total volume Pre Embol ChSDs should be expected. According to the coefficient of determination R^2 of the resulting model, 18.4% of the observed variance of Total volume Pre Embol ChSDs were explained..

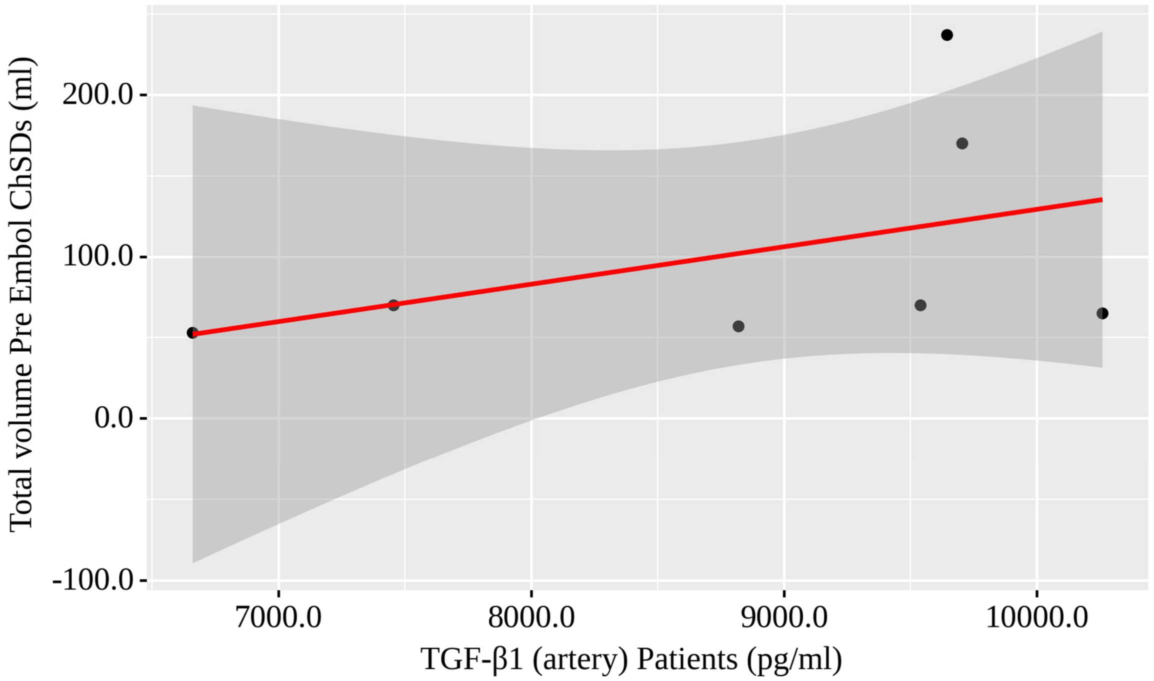

Figure 111 – Regression line characterizing the dependence of Total volume Pre Embol ChSDs from TGF-β1 (artery) Patients

Analysis of TGF-β1 (artery) Patients was performed conditioning on Rebleeding on CT scans.

Table 81 – Analysis of TGF-β1 (artery) Patients conditioning on Rebleeding on CT scans

Variable	Categories	TGF-β1 (artery) Patients (pg/ml)			p
		M ± SD	95% CI	n	
Rebleeding on CT scans	none	8125 ± 1891	3427 – 12823	3	0.227
	rebleeded	9428 ± 411	8774 – 10081	4	

When comparing of TGF-β1 (artery) Patients depending on Rebleeding on CT scans no statistically significant differences were revealed (p = 0.227) *(applied method: Student's t-test)*.

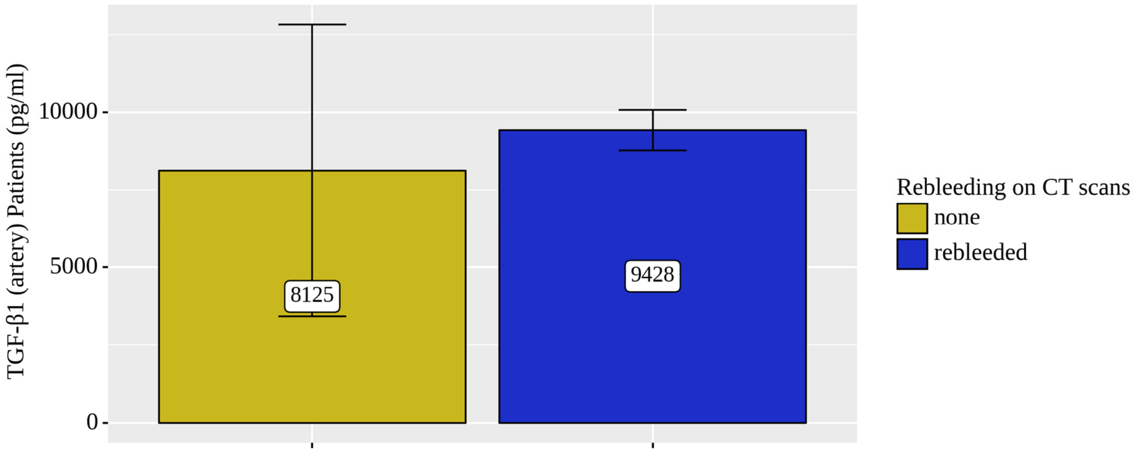

Figure 112 – Analysis of TGF-β1 (artery) Patients conditioning on Rebleeding on CT scans

When evaluating the dependence of the probability of rebleeded on the TGF-β1 (artery) Patients using the ROC analysis, the following curve was obtained.

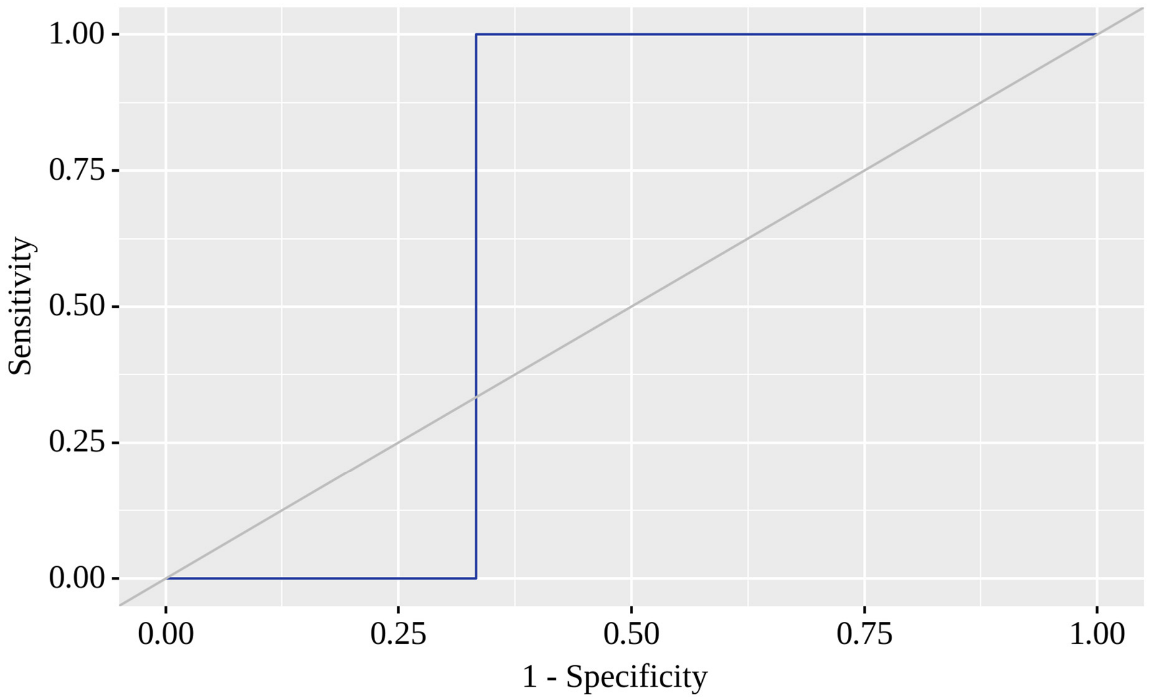

Figure 113 – ROC-curve characterizing the dependence of the probability Rebleeding on CT scans on TGF-β1 (artery) Patients

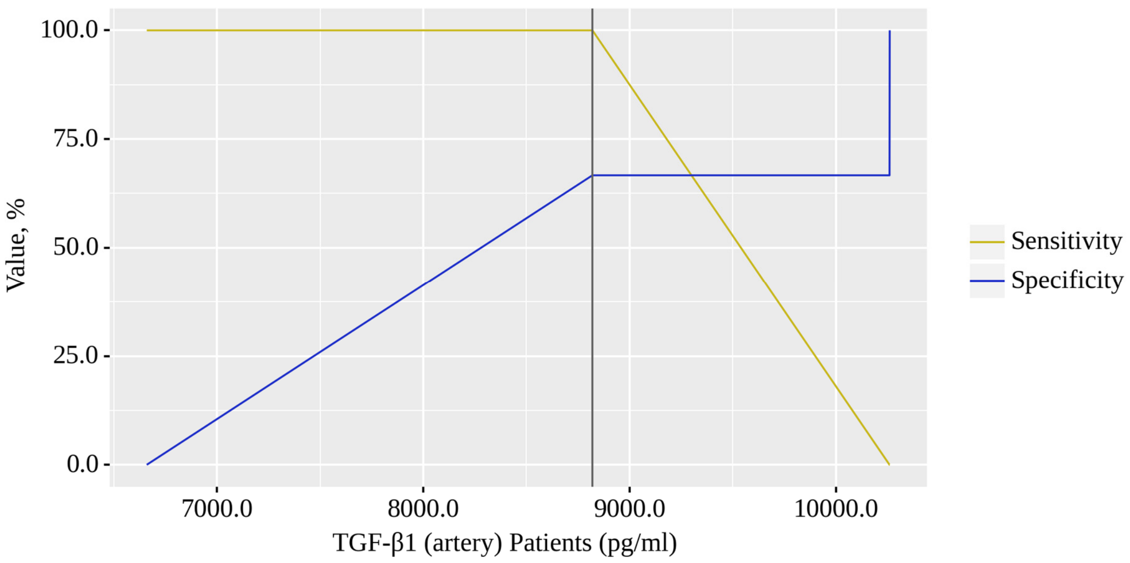

Figure 114 - Analysis of the sensitivity and specificity of Rebleeding on CT scans depending on TGF-β1 (artery) Patients

Table 82 – Threshold TGF-β1 (artery) Patients

Threshold	Sensitivity (Se), %	Specificity (Sp), %	PPV	NPV
**8820**	**100.0**	**66.7**	**80.0**	**100.0**

The area under the ROC curve comprised 0.667 ± 0.217 with 95% CI: 0.241 - 1.000. The resulting model was not statistically significant ($p = 0.480$).

The cut-off value of TGF-β1 (artery) Patients which corresponds to the highest Youden's J statistic is 8820.000 pg/ml. If TGF-β1 (artery) Patients was greater than or equal to this value, rebleeded was predicted. The sensitivity and specificity of the method were 100.0% and 66.7%, respectively.

We performed analysis of TGF-β1 (artery) Patients conditioning on Surgery.

Table 83 – Analysis of TGF-β1 (artery) Patients conditioning on Surgery

Variable	Categories	TGF-β1 (artery) Patients (pg/ml)			p
		M ± SD	95% CI	n	
Surgery	none	8395 ± 1566	4504 – 12286	3	0.464
	operated	9225 ± 1222	7281 – 11169	4	

When comparing of TGF-β1 (artery) Patients depending on Surgery there were no statistically significant differences (p = 0.464) *(applied method: Student's t-test)*.

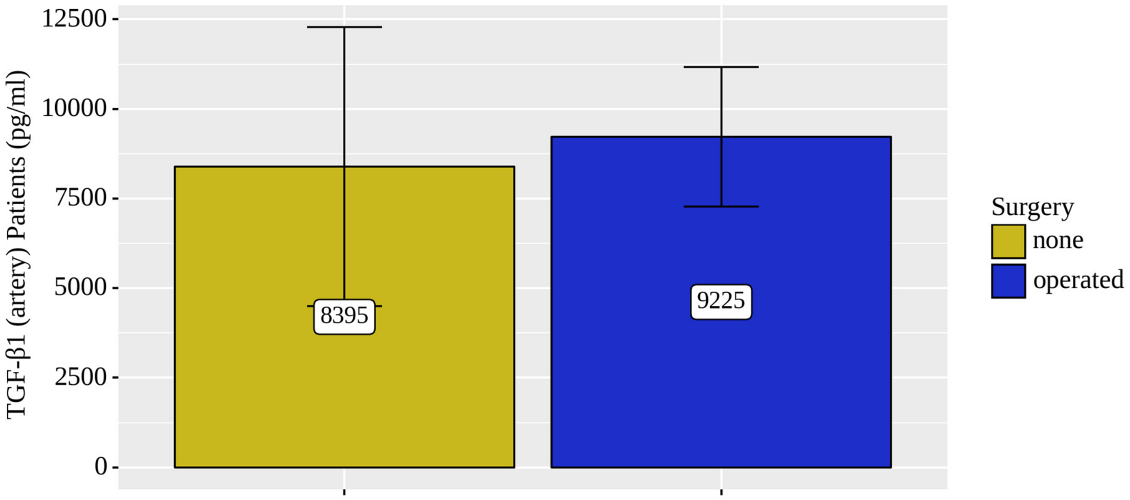

Figure 115 – Analysis of TGF-β1 (artery) Patients conditioning on Surgery

When evaluating the dependence of the probability of operated on the TGF-β1 (artery) Patients using the ROC analysis, the following curve was obtained.

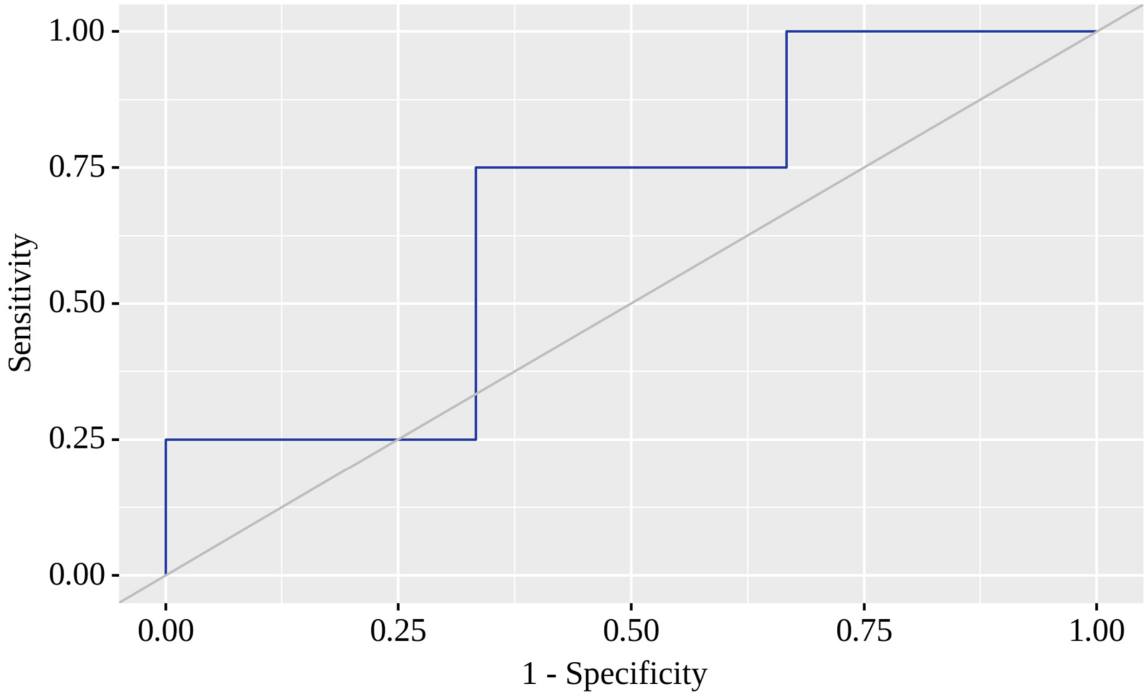

Figure 116 – ROC-curve characterizing the dependence of the probability Surgery on TGF-β1 (artery) Patients

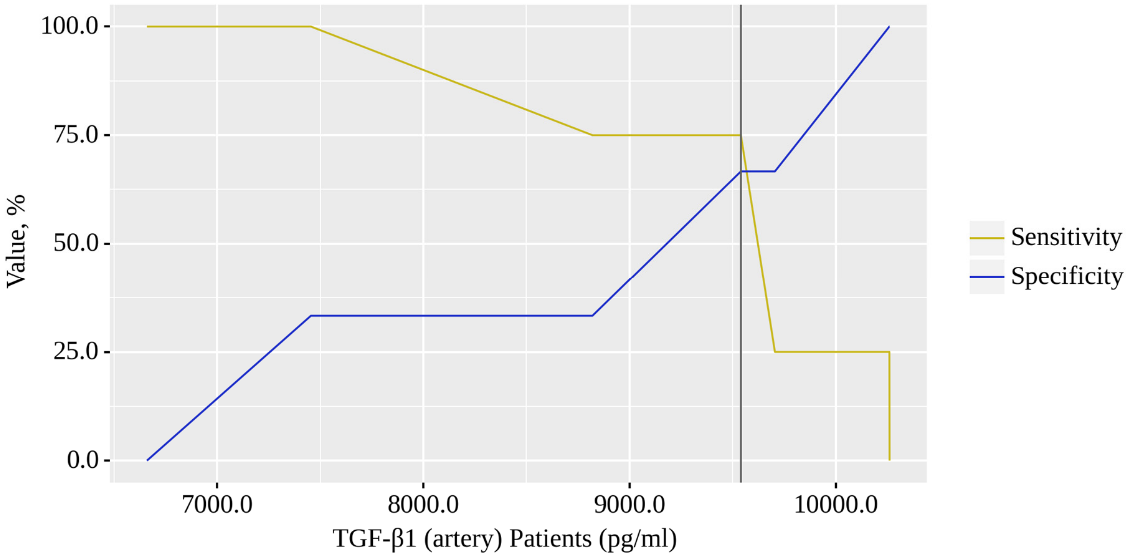

Figure 117 - Analysis of the sensitivity and specificity of Surgery depending on TGF-β1 (artery) Patients

Table 84 – Threshold TGF-β1 (artery) Patients

Threshold	Sensitivity (Se), %	Specificity (Sp), %	PPV	NPV
**9540**	**75.0**	**66.7**	**75.0**	**66.7**

The area under the ROC curve comprised 0.667 ± 0.217 with 95% CI: 0.241 - 1.000. The resulting model was not statistically significant ($p = 0.480$).

The cut-off value of TGF-β1 (artery) Patients which corresponds to the highest Youden's J statistic is 9540.000 pg/ml. If TGF-β1 (artery) Patients was greater than or equal to this value, operated was predicted. The sensitivity and specificity of the method were 75.0% and 66.7%, respectively.

Analysis of PDGF-β (vein) P/HV was performed conditioning on Patients/Healthy volunteers.

Table 85 – Analysis of PDGF-β (vein) P/HV conditioning on Patients/Healthy volunteers

Variable	Categories	PDGF-β (vein) P/HV (pg/ml)			p
		M ± SD	95% CI	n	
Patients/Healthy volunteers	patient	2372 ± 1371	1226 – 3519	8	0.038*
	healthy volunteer	3611 ± 371	3442 – 3779	21	

* – differences are statistically significant ($p < 0.05$)

According to the data obtained when comparing of PDGF-β (vein) P/HV statistically significant differences were revealed depending on Patients/Healthy volunteers ($p = 0.038$) *(applied method: Welch's t-test)*.

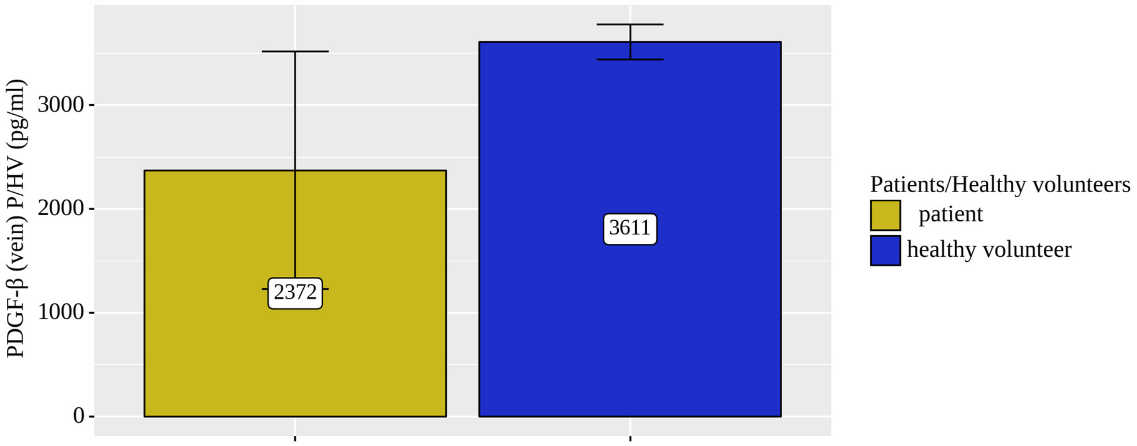

Figure 118 – Analysis of PDGF-β (vein) P/HV conditioning on Patients/Healthy volunteers

When evaluating the dependence of the probability of healthy volunteer on the PDGF-β (vein) P/HV using the ROC analysis, the following curve was obtained.

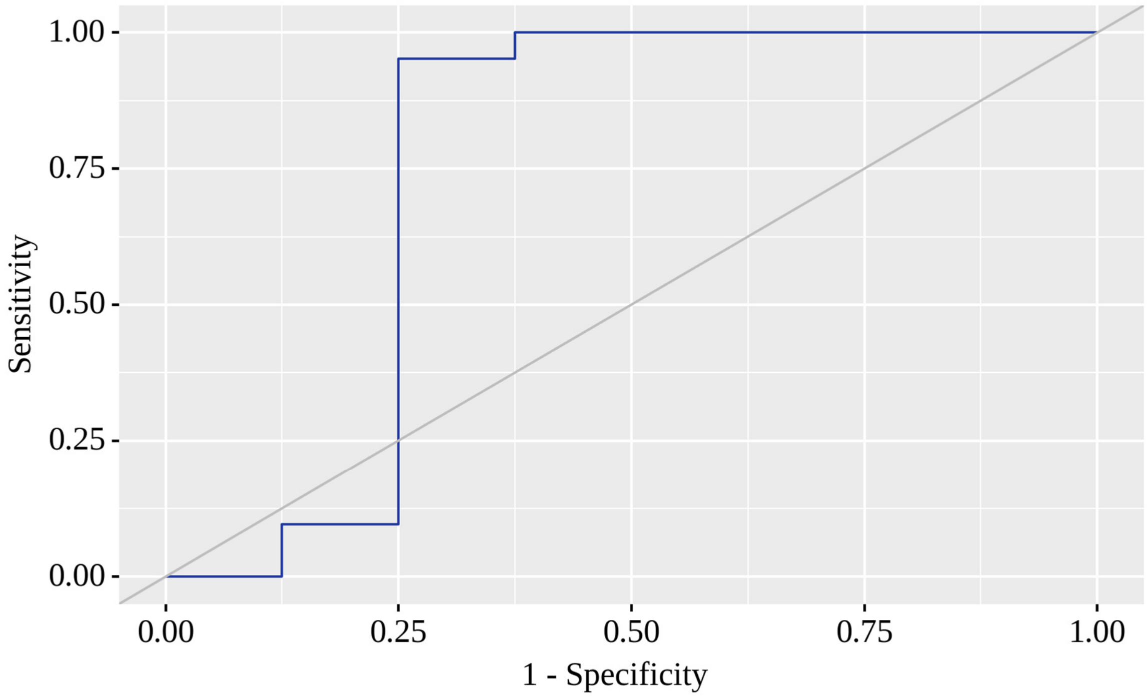

Figure 119 – ROC-curve characterizing the dependence of the probability Patients/Healthy volunteers on PDGF-β (vein) P/HV

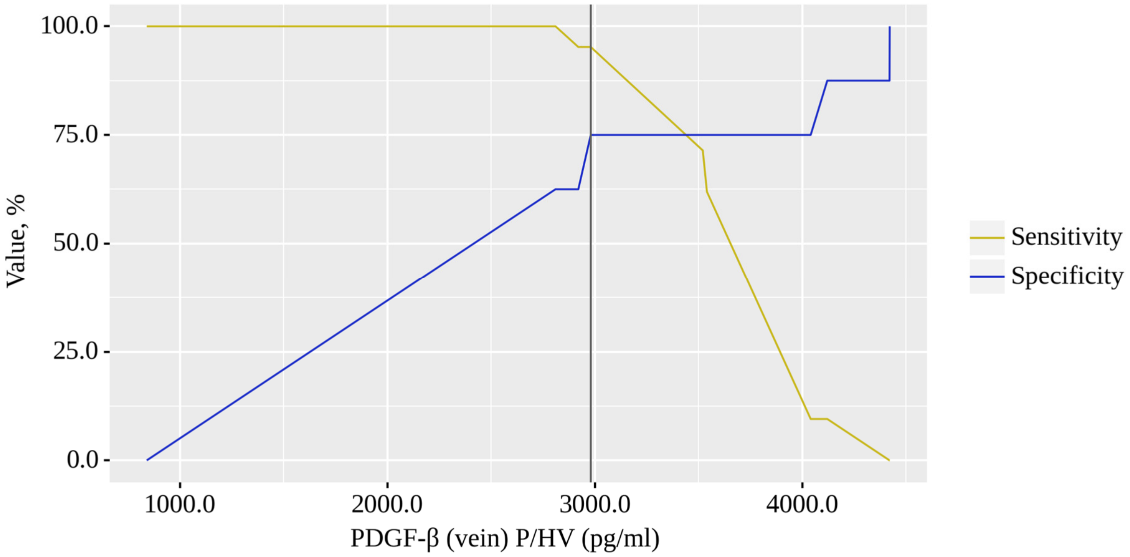

Figure 120 - Analysis of the sensitivity and specificity of Patients/Healthy volunteers depending on PDGF-β (vein) P/HV

Table 86 – Threshold PDGF-β (vein) P/HV

Threshold	Sensitivity (Se), %	Specificity (Sp), %	PPV	NPV
3540	61.9	75.0	86.7	42.9
**3520**	**71.4**	**75.0**	**88.2**	**50.0**
2980	95.2	75.0	90.9	85.7
2920	95.2	62.5	87.0	83.3
2810	100.0	62.5	87.5	100.0

The area under the ROC curve comprised 0.756 ± 0.093 with 95% CI: 0.573 - 0.939. The resulting model was statistically significant ($p = 0.036$).

The cut-off value of PDGF-β (vein) P/HV which corresponds to the highest Youden's J statistic is 2980.000 pg/ml. If PDGF-β (vein) P/HV was greater than or equal to this value, healthy volunteer was predicted. The sensitivity and specificity of the method were 95.2% and 75.0%, respectively.

Analysis of PDGF-β (vein) Patients was performed conditioning on Sides ChSDH.

Table 87 – Analysis of PDGF-β (vein) Patients conditioning on Sides ChSDH

Variable	Categories	PDGF-β (vein) Patients (pg/ml)			p
		Me	$Q_1 – Q_3$	n	

Sides ChSDH	Bilateral ChSDH	3510	3055 – 3965	2	0.182
	Monolateral ChSDH	1640	995 – 2675	6	

When comparing of PDGF-β (vein) Patients depending on Sides ChSDH there were no statistically significant differences (p = 0.182) *(applied method: Mann-Whitney U-test)*.

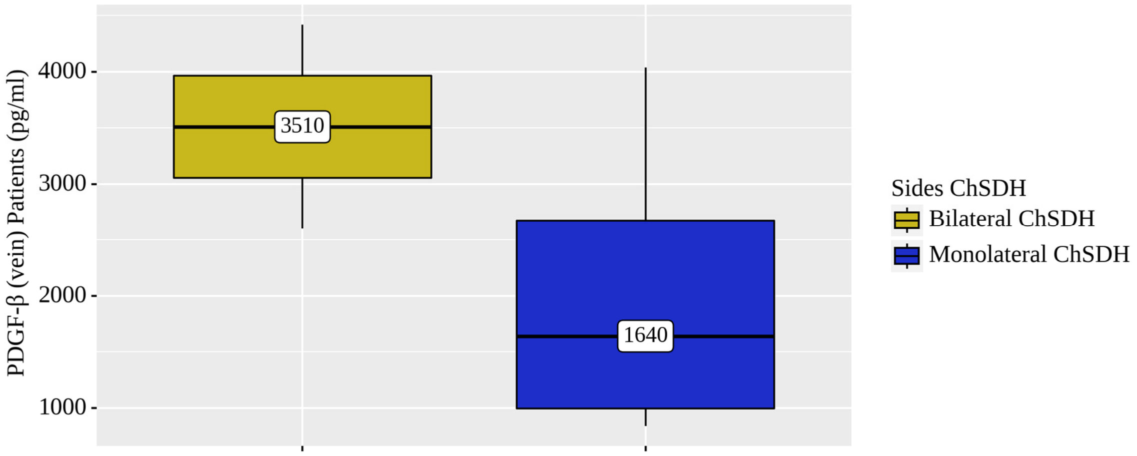

Figure 121 – Analysis of PDGF-β (vein) Patients conditioning on Sides ChSDH

When evaluating the dependence of the probability of Monolateral ChSDH on the PDGF-β (vein) Patients using the ROC analysis, the following curve was obtained.

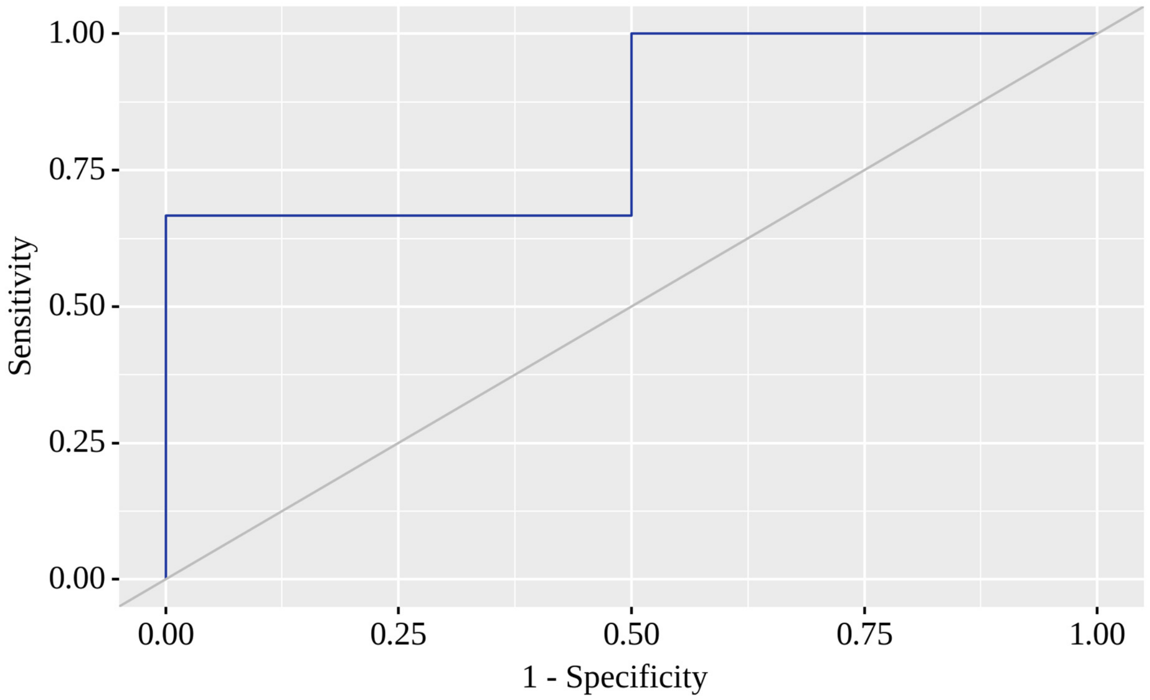

Figure 122 – ROC-curve characterizing the dependence of the probability Sides ChSDH on PDGF-β (vein) Patients

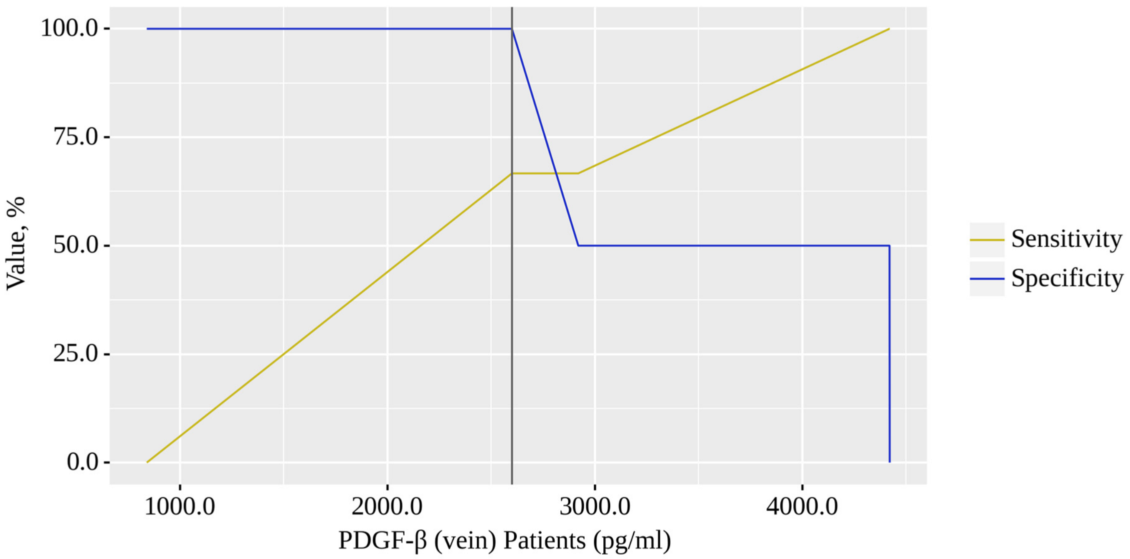

Figure 123 - Analysis of the sensitivity and specificity of Sides ChSDH depending on PDGF-β (vein) Patients

Table 88 – Threshold PDGF-β (vein) Patients

Threshold	Sensitivity (Se), %	Specificity (Sp), %	PPV	NPV
4420	100.0	50.0	85.7	100.0

**2920**	**66.7**	**50.0**	**80.0**	**33.3**
2600	66.7	100.0	100.0	50.0

The area under the ROC curve comprised 0.833 ± 0.199 with 95% CI: 0.444 - 1.000. The resulting model was not statistically significant ($p = 0.182$).

The cut-off value of PDGF-β (vein) Patients which corresponds to the highest Youden's J statistic is 2600.000 pg/ml. If PDGF-β (vein) Patients was less than this value, Monolateral ChSDH was predicted. The sensitivity and specificity of the method were 66.7% and 100.0%, respectively.

We performed analysis of PDGF-β (vein) Patients conditioning on Rebleeding on CT scans.

Table 89 – Analysis of PDGF-β (vein) Patients conditioning on Rebleeding on CT scans

Variable	Categories	PDGF-β (vein) Patients (pg/ml)			p
		M ± SD	95% CI	n	
Rebleeding on CT scans	none	2355 ± 1337	227 – 4483	4	0.974
	rebleeded	2390 ± 1611	-173 – 4953	4	

When comparing of PDGF-β (vein) Patients depending on Rebleeding on CT scans no statistically significant differences were revealed ($p = 0.974$) *(applied method: Student's t-test)*.

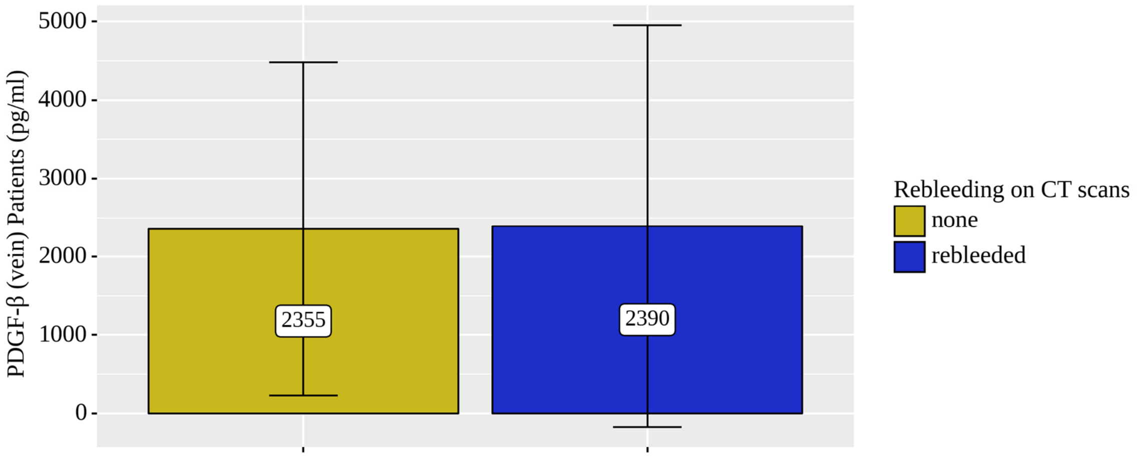

Figure 124 – Analysis of PDGF-β (vein) Patients conditioning on Rebleeding on CT scans

When evaluating the dependence of the probability of rebleeded on the PDGF-β (vein) Patients using the ROC analysis, the following curve was obtained.

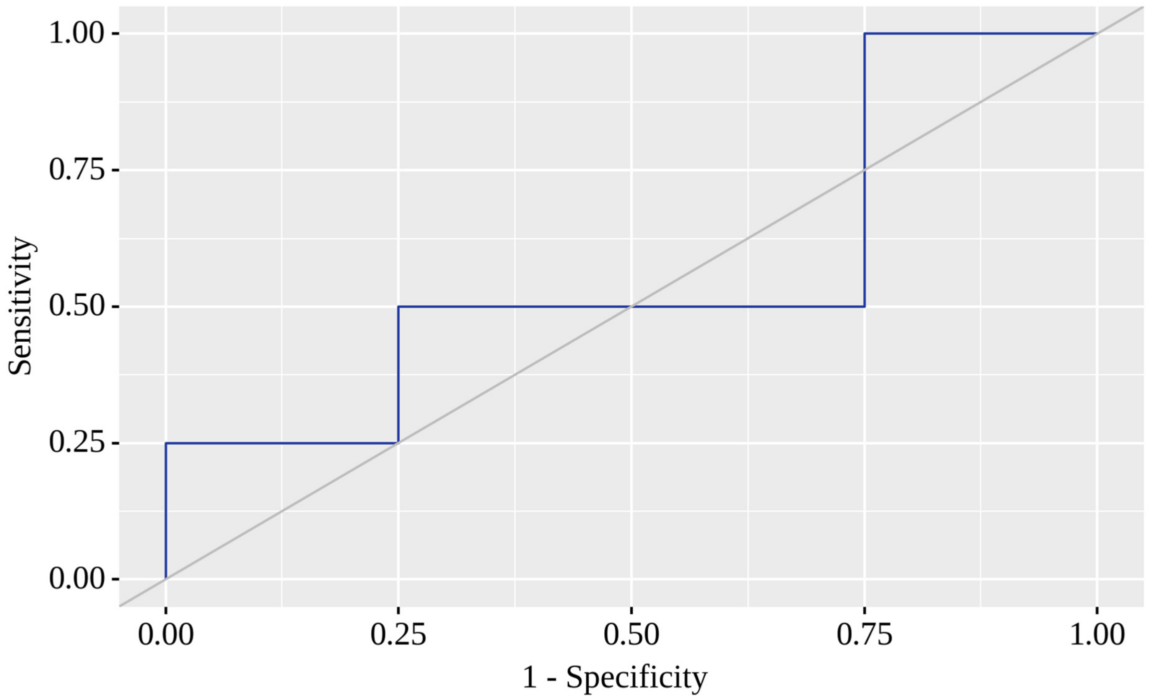

Figure 125 – ROC-curve characterizing the dependence of the probability Rebleeding on CT scans on PDGF-β (vein) Patients

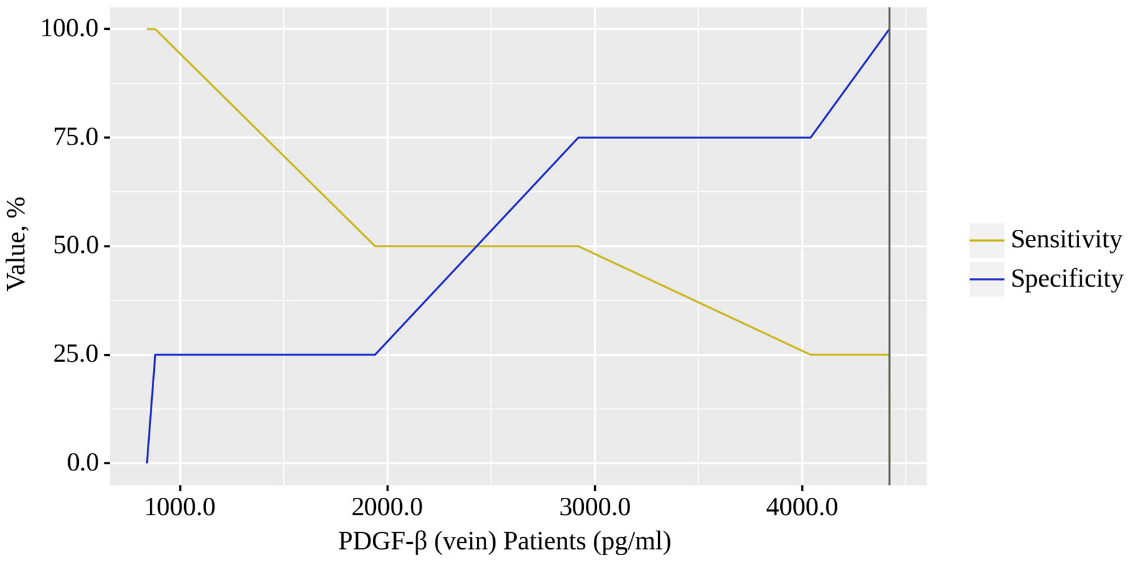

Figure 126 - Analysis of the sensitivity and specificity of Rebleeding on CT scans depending on PDGF-β (vein) Patients

Table 90 – Threshold PDGF-β (vein) Patients

Threshold	Sensitivity (Se), %	Specificity (Sp), %	PPV	NPV
**2920**	**50.0**	**75.0**	**66.7**	**60.0**

The area under the ROC curve comprised 0.562 ± 0.215 with 95% CI: 0.142 - 0.983. The resulting model was not statistically significant ($p = 0.773$).

The cut-off value of PDGF-β (vein) Patients which corresponds to the highest Youden's J statistic is 4420.000 pg/ml. If PDGF-β (vein) Patients was greater than or equal to this value, rebleeded was predicted. The sensitivity and specificity of the method were 25.0% and 100.0%, respectively.

We performed a correlation analysis of the association between PDGF-β (vein) Patients and Total volume Pre Embol ChSDs.

Table 91 – Results of the correlation analysis of the association between PDGF-β (vein) Patients and Total volume Pre Embol ChSDs

Variable	Correlation characteristics		
	ρ	Strength of the association assesed using Chaddock scale	p
PDGF-β (vein) Patients – Total volume Pre Embol ChSDs	0.503	Close	0.204

A close correlation positive association between Total volume Pre Embol ChSDs and PDGF-β (vein) Patients was estimated.

Observed dependence of Total volume Pre Embol ChSDs from PDGF-β (vein) Patients is described by a linear regression equation:

$$Y_{\text{Total volume Pre Embol ChSDs}} = 0.032 \times X_{\text{PDGF-β (vein) Patients}} + 26.48$$

With an 1 pg/ml increase of PDGF-β (vein) Patients 0.032 ml change of Total volume Pre Embol ChSDs should be expected. According to the coefficient of determination R^2 of the resulting model, 44.0% of the observed variance of Total volume Pre Embol ChSDs were explained..

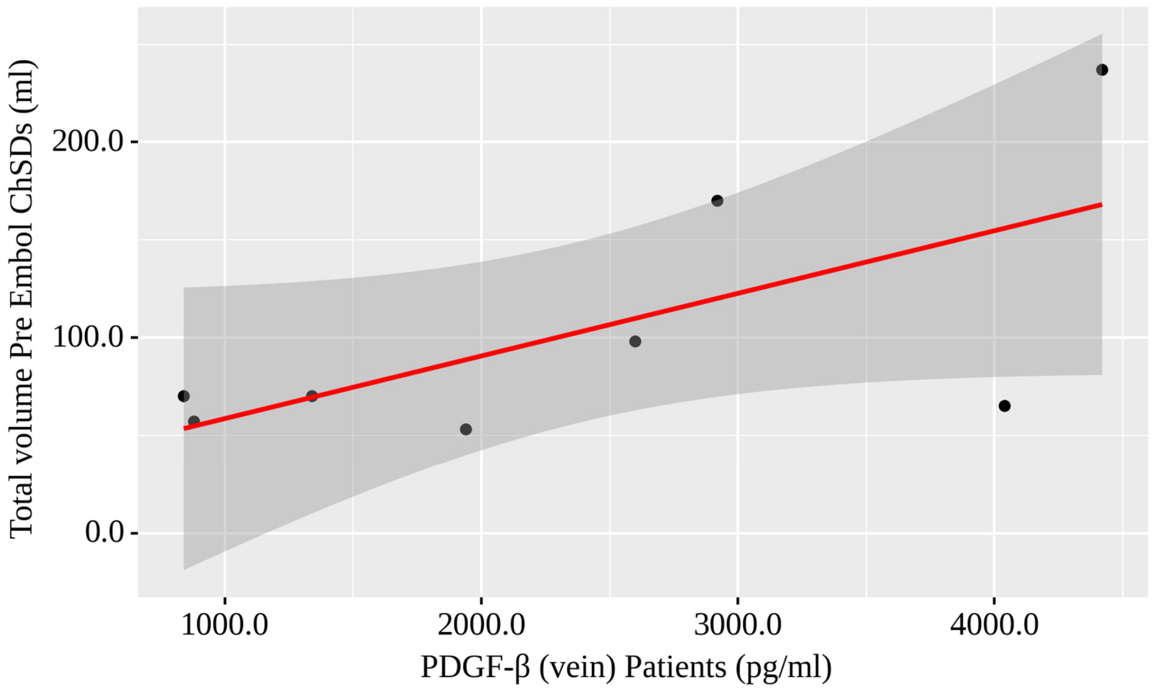

Figure 127 – Regression line characterizing the dependence of Total volume Pre Embol ChSDs from PDGF-β (vein) Patients

We performed analysis of PDGF-β (vein) Patients conditioning on Surgery.

Table 92 – Analysis of PDGF-β (vein) Patients conditioning on Surgery

Variable	Categories	PDGF-β (vein) Patients (pg/ml)			p
		M ± SD	95% CI	n	
Surgery	none	2085 ± 901	651 – 3519	4	0.601
	operated	2660 ± 1831	-253 – 5573	4	

When comparing of PDGF-β (vein) Patients depending on Surgery there were no statistically significant differences (p = 0.601) *(applied method: Welch's t-test)*.

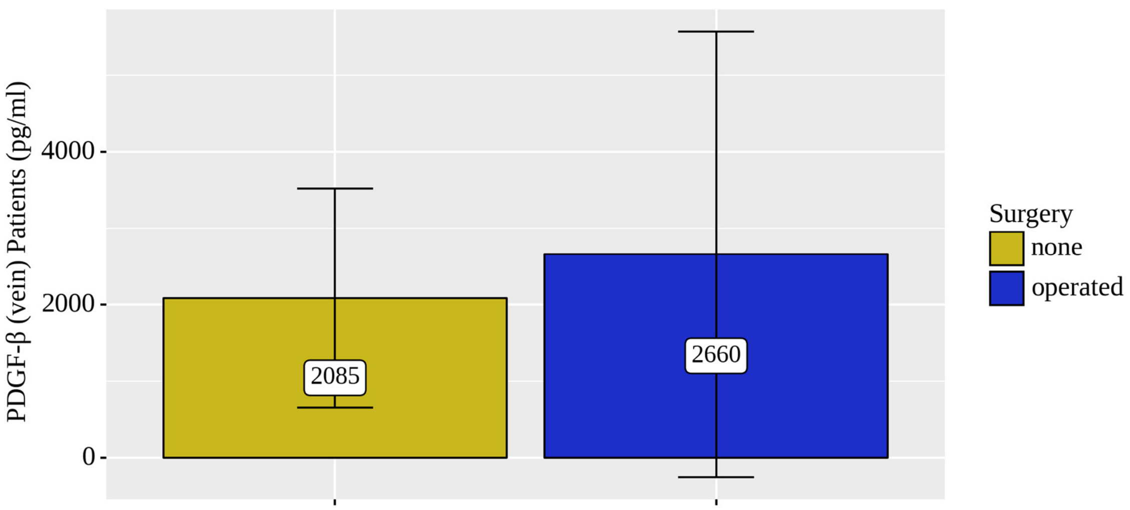

Figure 128 – Analysis of PDGF-β (vein) Patients conditioning on Surgery

When evaluating the dependence of the probability of operated on the PDGF-β (vein) Patients using the ROC analysis, the following curve was obtained.

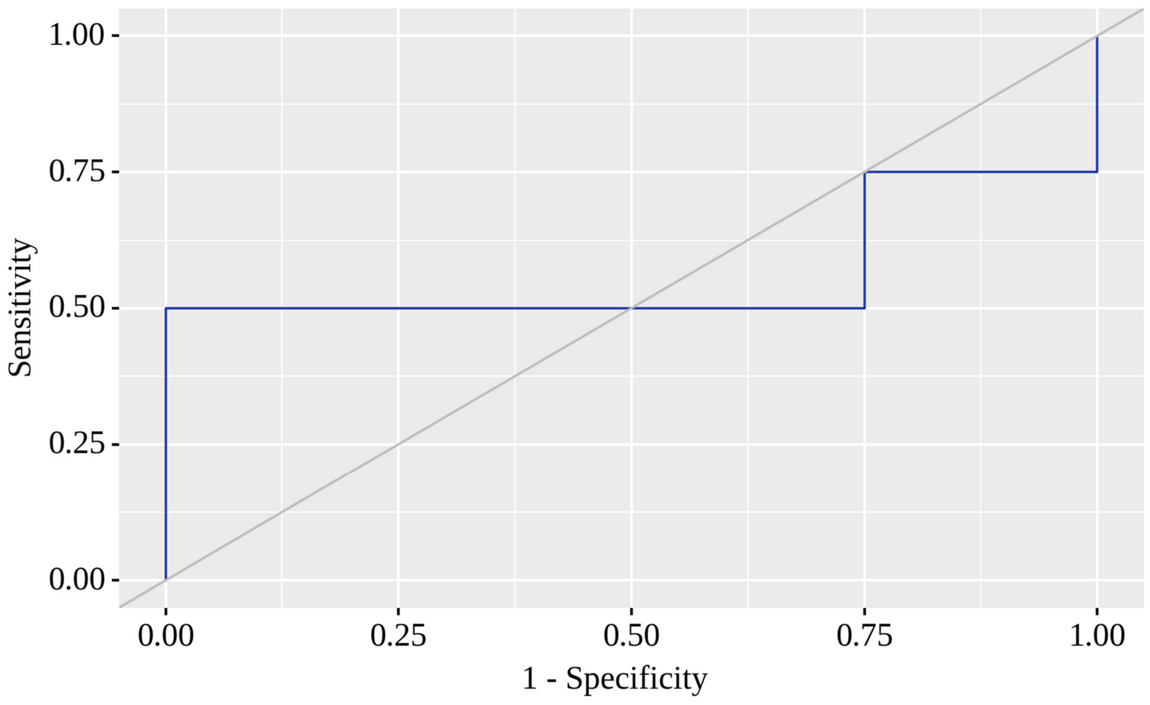

Figure 129 – ROC-curve characterizing the dependence of the probability Surgery on PDGF-β (vein) Patients

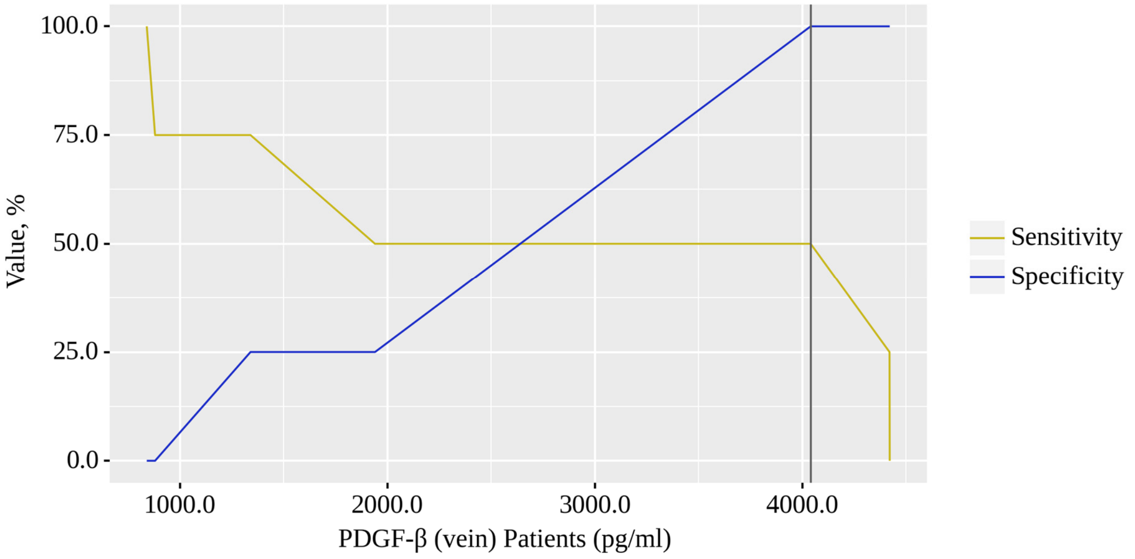

Figure 130 - Analysis of the sensitivity and specificity of Surgery depending on PDGF-β (vein) Patients

Table 93 – Threshold PDGF-β (vein) Patients

Threshold	Sensitivity (Se), %	Specificity (Sp), %	PPV	NPV
**4040**	**50.0**	**100.0**	**100.0**	**66.7**

The area under the ROC curve comprised 0.562 ± 0.215 with 95% CI: 0.142 - 0.983. The resulting model was not statistically significant (p = 0.773).

The cut-off value of PDGF-β (vein) Patients which corresponds to the highest Youden's J statistic is 4040.000 pg/ml. If PDGF-β (vein) Patients was greater than or equal to this value, operated was predicted. The sensitivity and specificity of the method were 50.0% and 100.0%, respectively.

Correlation analysis of the association between PDGF-β (artery) Patients and PDGF-β (vein) Patients was performed.

Table 94 – Results of the correlation analysis of the association between PDGF-β (artery) Patients and PDGF-β (vein) Patients

Variable	Correlation characteristics		
	r_{xy}	Strength of the association assesed using Chaddock scale	p
PDGF-β (artery) Patients – PDGF-β (vein) Patients	0.560	Close	0.191

A close correlation positive association between PDGF-β (vein) Patients and PDGF-β (artery) Patients was estimated.

Observed dependence of PDGF-β (vein) Patients from PDGF-β (artery) Patients is described by a linear regression equation:

$$Y_{\text{PDGF-β (vein) Patients}} = 0.794 \times X_{\text{PDGF-β (artery) Patients}} + 209.55$$

With an 1 pg/ml increase of PDGF-β (artery) Patients 0.794 pg/ml change of PDGF-β (vein) Patients should be expected. According to the coefficient of determination R^2 of the resulting model, 31.4% of the observed variance of PDGF-β (vein) Patients were explained..

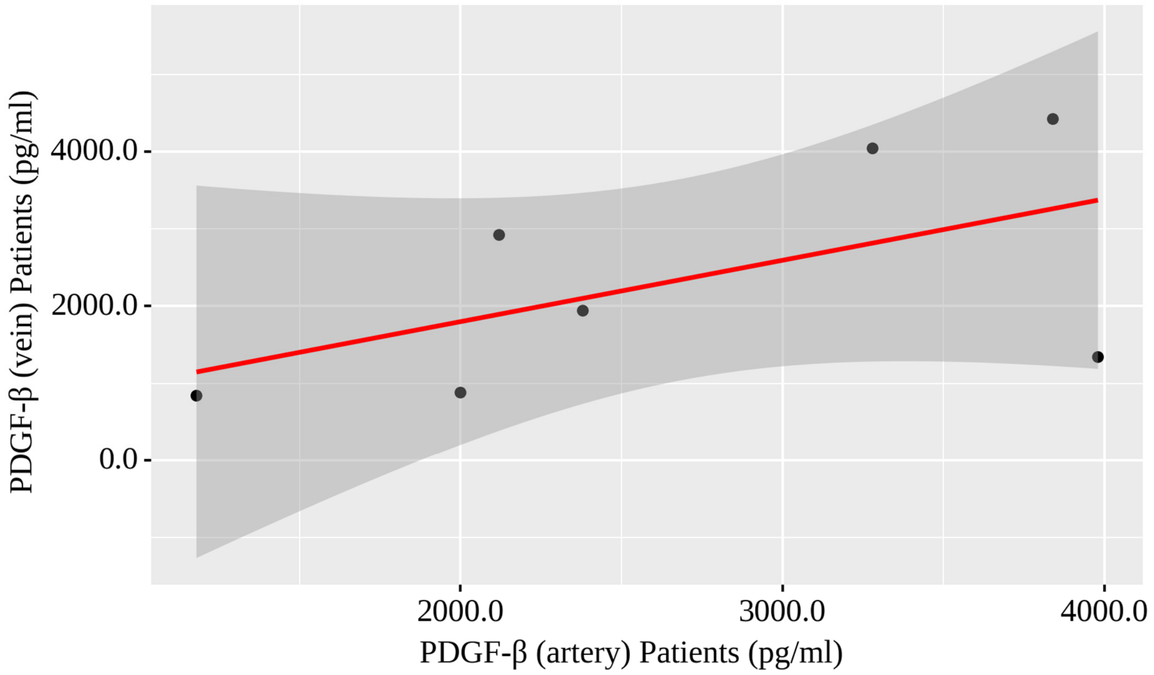

Figure 131 – Regression line characterizing the dependence of PDGF-β (vein) Patients from PDGF-β (artery) Patients

We performed analysis of PDGF-β (artery) Patients conditioning on Sides ChSDH.

Table 95 – Analysis of PDGF-β (artery) Patients conditioning on Sides ChSDH

Variable	Categories	PDGF-β (artery) Patients (pg/ml)			p
		Me	$Q_1 – Q_3$	n	
Sides ChSDH	Bilateral ChSDH	3840	3840 – 3840	1	0.317

	Monolateral ChSDH	2250	2030 – 3055	6	

When comparing of PDGF-β (artery) Patients depending on Sides ChSDH no statistically significant differences were revealed (p = 0.317) *(applied method: Mann-Whitney U-test)*.

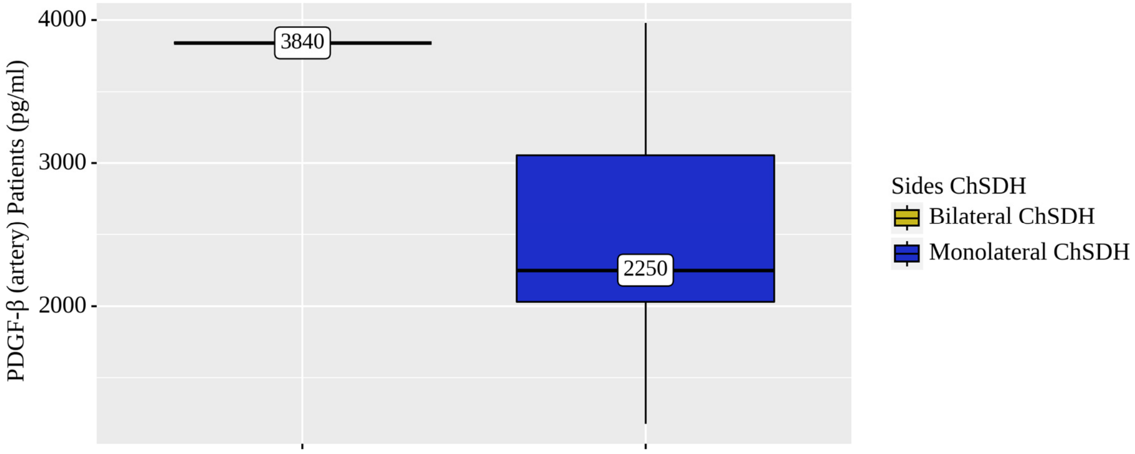

Figure 132 – Analysis of PDGF-β (artery) Patients conditioning on Sides ChSDH

When evaluating the dependence of the probability of Monolateral ChSDH on the PDGF-β (artery) Patients using the ROC analysis, the following curve was obtained.

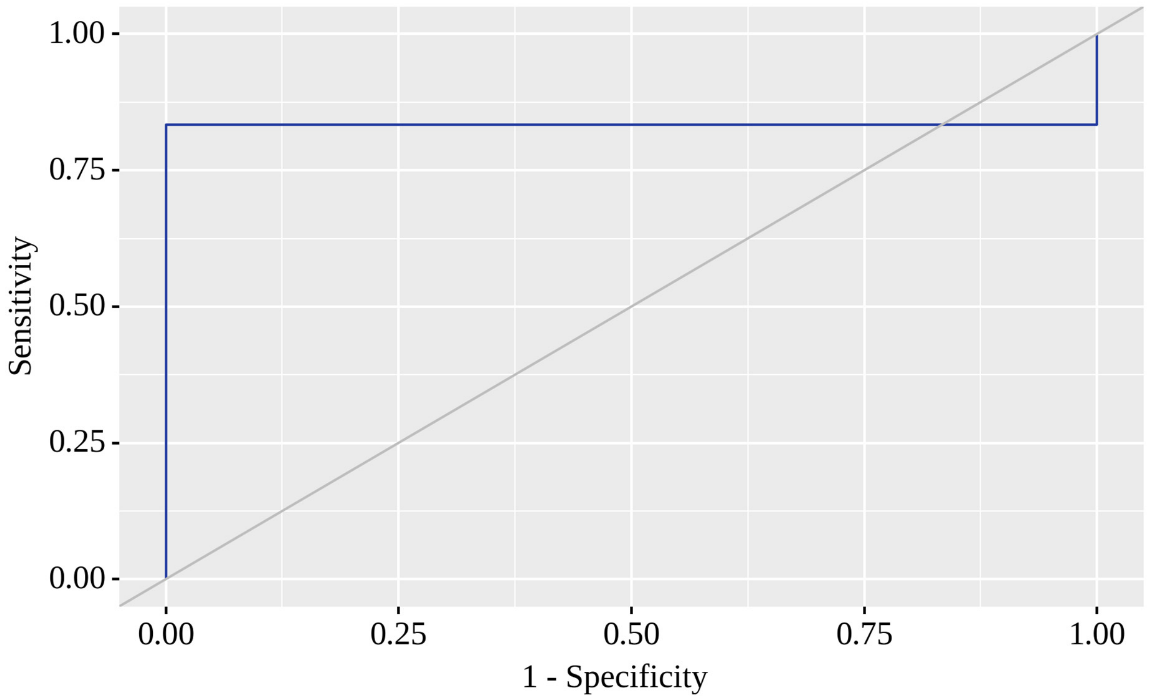

Figure 133 – ROC-curve characterizing the dependence of the probability Sides ChSDH on PDGF-β (artery) Patients

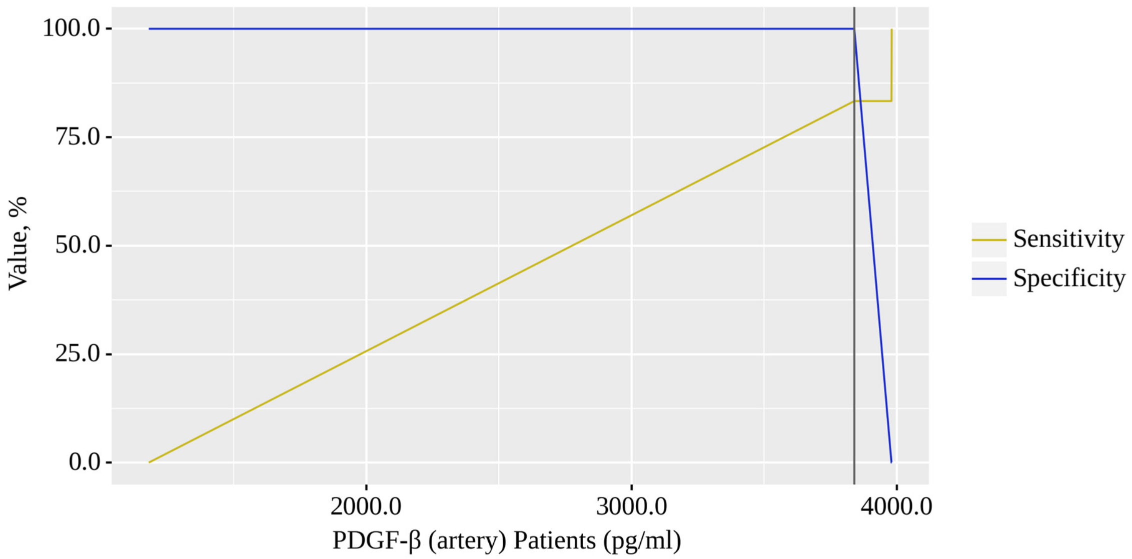

Figure 134 - Analysis of the sensitivity and specificity of Sides ChSDH depending on PDGF-β (artery) Patients

Table 96 – Threshold PDGF-β (artery) Patients

Threshold	Sensitivity (Se), %	Specificity (Sp), %	PPV	NPV
**3840**	**83.3**	**100.0**	**100.0**	**50.0**

The area under the ROC curve comprised 0.833 ± 0.275 with 95% CI: 0.294 - 1.000. The resulting model was not statistically significant ($p = 0.317$).

The cut-off value of PDGF-β (artery) Patients which corresponds to the highest Youden's J statistic is 3840.000 pg/ml. If PDGF-β (artery) Patients was less than this value, Monolateral ChSDH was predicted. The sensitivity and specificity of the method were 83.3% and 100.0%, respectively.

We performed a correlation analysis of the association between PDGF-β (artery) Patients and Total volume Pre Embol ChSDs.

Table 97 – Results of the correlation analysis of the association between PDGF-β (artery) Patients and Total volume Pre Embol ChSDs

Variable	Correlation characteristics		
	ρ	Strength of the association assesed using Chaddock scale	p
PDGF-β (artery) Patients – Total volume Pre Embol ChSDs	0.252	Weak	0.585

A weak correlation positive association between Total volume Pre Embol ChSDs and PDGF-β (artery) Patients was estimated.

Observed dependence of Total volume Pre Embol ChSDs from PDGF-β (artery) Patients is described by a linear regression equation:

$$Y_{\text{Total volume Pre Embol ChSDs}} = 0.023 \times X_{\text{PDGF-}\beta\text{ (artery) Patients}} + 42.166$$

With an 1 pg/ml increase of PDGF-β (artery) Patients 0.023 ml change of Total volume Pre Embol ChSDs should be expected. According to the coefficient of determination R^2 of the resulting model, 11.0% of the observed variance of Total volume Pre Embol ChSDs were explained..

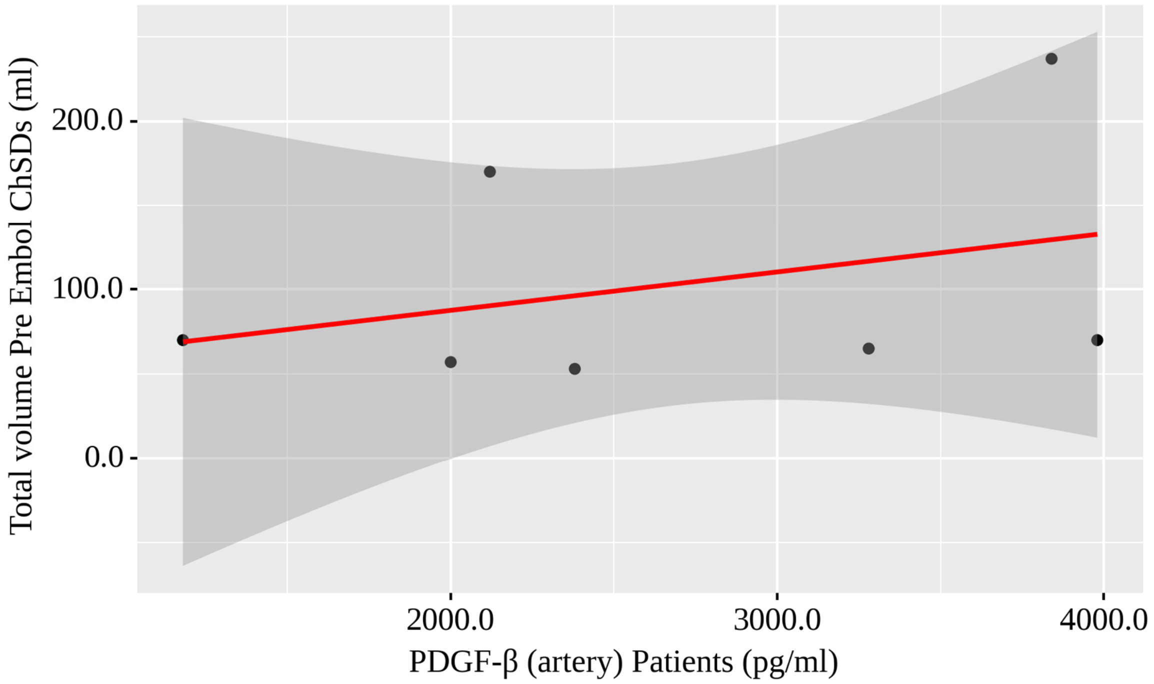

Figure 135 – Regression line characterizing the dependence of Total volume Pre Embol ChSDs from PDGF-β (artery) Patients

We performed analysis of PDGF-β (artery) Patients conditioning on Rebleeding on CT scans.

Table 98 – Analysis of PDGF-β (artery) Patients conditioning on Rebleeding on CT scans

Variable	Categories	PDGF-β (artery) Patients (pg/ml)			p
		M ± SD	95% CI	n	
Rebleeding on CT scans	none	2280 ± 1054	-337 – 4897	3	0.425
	rebleeded	2985 ± 1071	1281 – 4689	4	

When comparing of PDGF-β (artery) Patients depending on Rebleeding on CT scans there were no statistically significant differences (p = 0.425) *(applied method: Student's t-test)*.

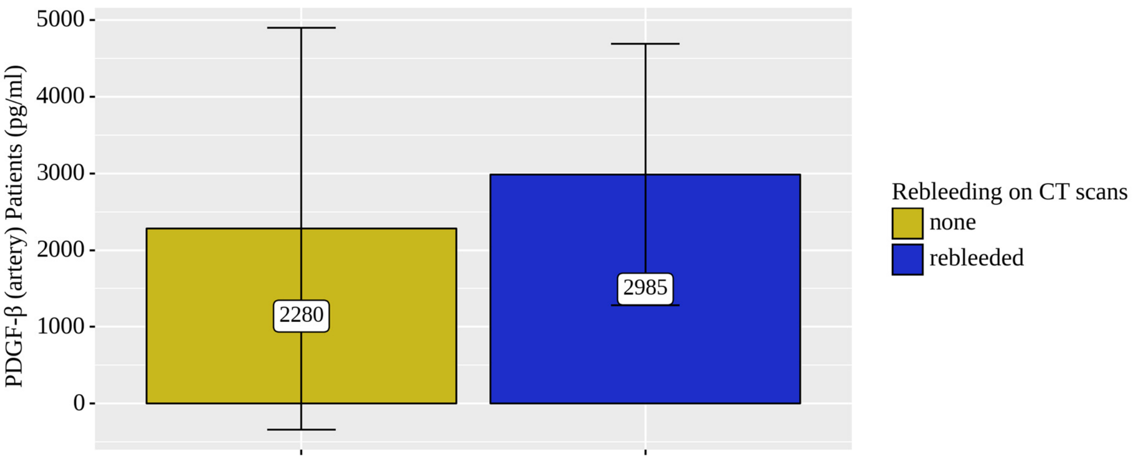

Figure 136 – Analysis of PDGF-β (artery) Patients conditioning on Rebleeding on CT scans

When evaluating the dependence of the probability of rebleeded on the PDGF-β (artery) Patients using the ROC analysis, the following curve was obtained.

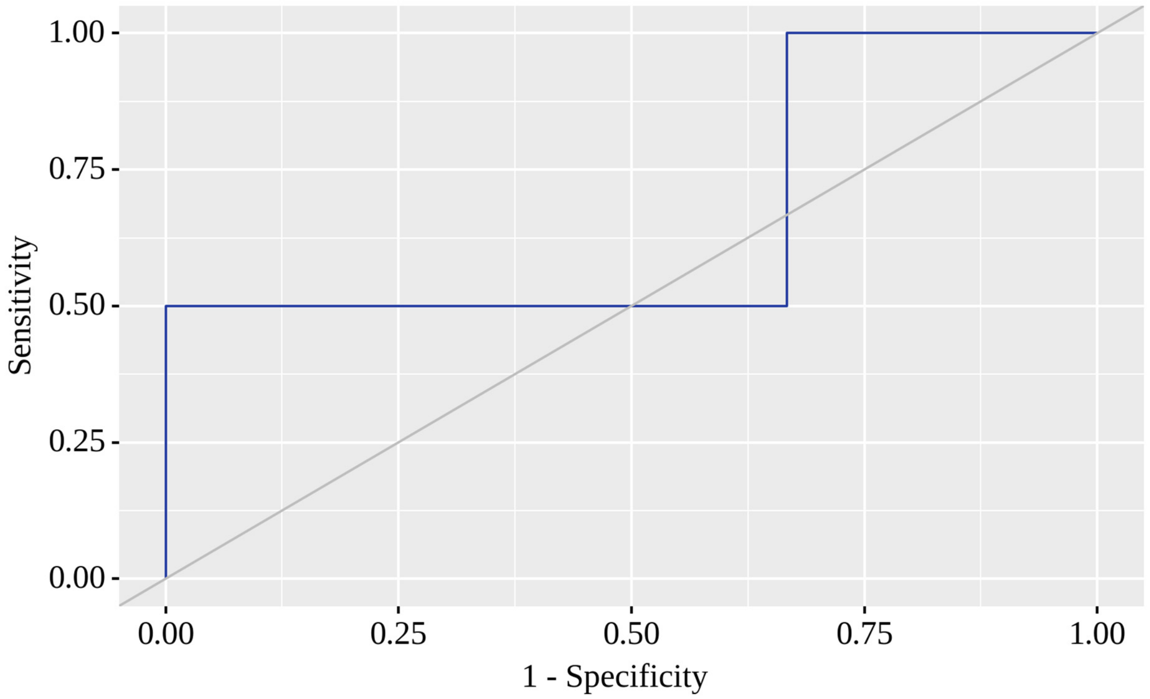

Figure 137 – ROC-curve characterizing the dependence of the probability Rebleeding on CT scans on PDGF-β (artery) Patients

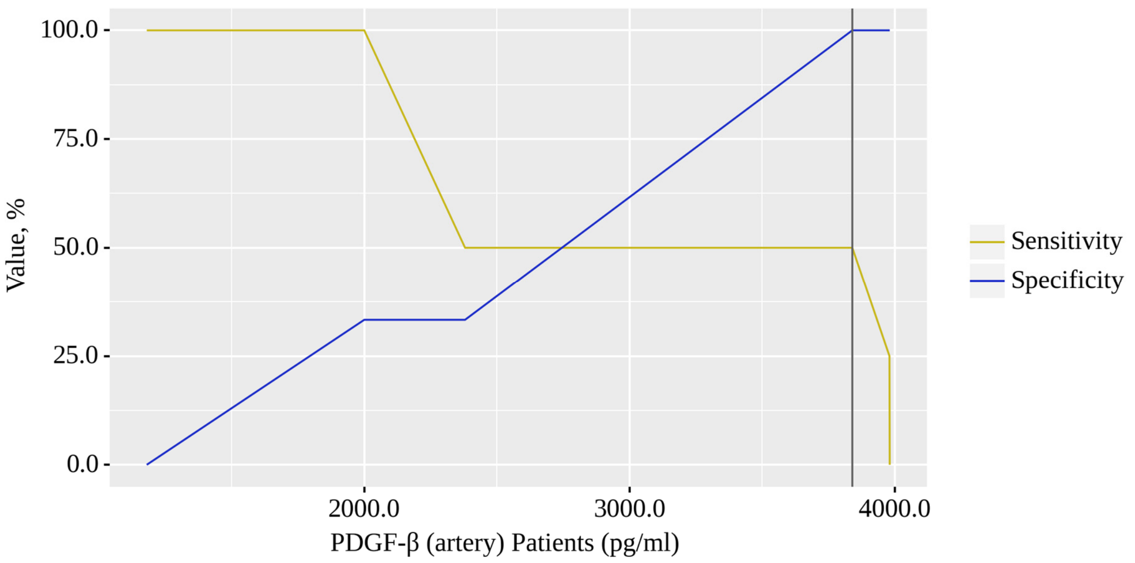

Figure 138 - Analysis of the sensitivity and specificity of Rebleeding on CT scans depending on PDGF-β (artery) Patients

Table 99 – Threshold PDGF-β (artery) Patients

Threshold	Sensitivity (Se), %	Specificity (Sp), %	PPV	NPV
**3840**	**50.0**	**100.0**	**100.0**	**60.0**

The area under the ROC curve comprised 0.667 ± 0.217 with 95% CI: 0.241 - 1.000. The resulting model was not statistically significant (p = 0.480).

The cut-off value of PDGF-β (artery) Patients which corresponds to the highest Youden's J statistic is 3840.000 pg/ml. If PDGF-β (artery) Patients was greater than or equal to this value, rebleeded was predicted. The sensitivity and specificity of the method were 50.0% and 100.0%, respectively.

We performed analysis of PDGF-β (artery) Patients conditioning on Surgery.

Table 100 – Analysis of PDGF-β (artery) Patients conditioning on Surgery

Variable	Categories	PDGF-β (artery) Patients (pg/ml)			p
		M ± SD	95% CI	n	
Surgery	none	2167 ± 194	1684 – 2649	3	0.295
	operated	3070 ± 1296	1008 – 5132	4	

When comparing of PDGF-β (artery) Patients depending on Surgery there were no statistically significant differences ($p = 0.295$) *(applied method: Student's t-test)*.

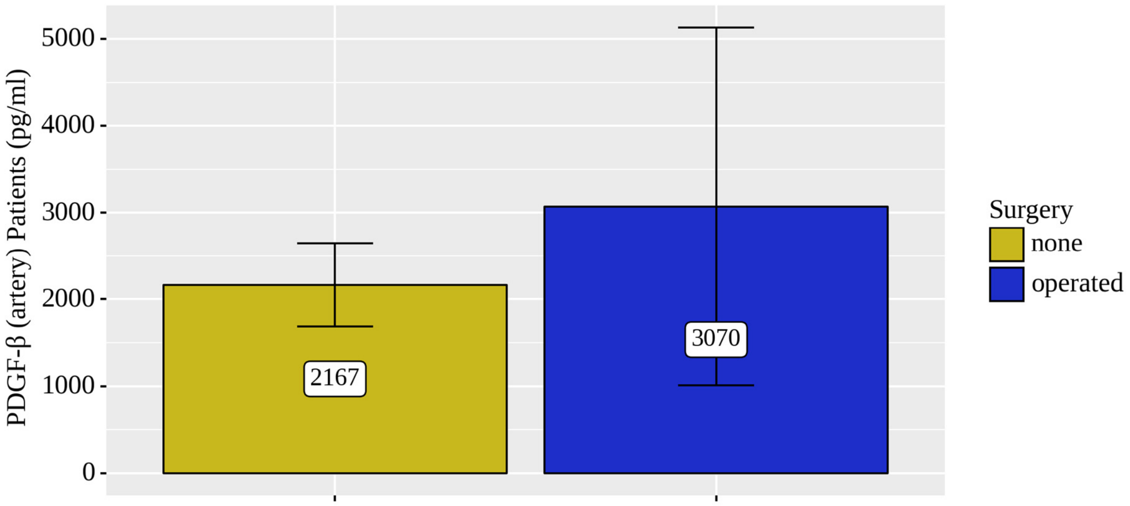

Figure 139 – Analysis of PDGF-β (artery) Patients conditioning on Surgery

When evaluating the dependence of the probability of operated on the PDGF-β (artery) Patients using the ROC analysis, the following curve was obtained.

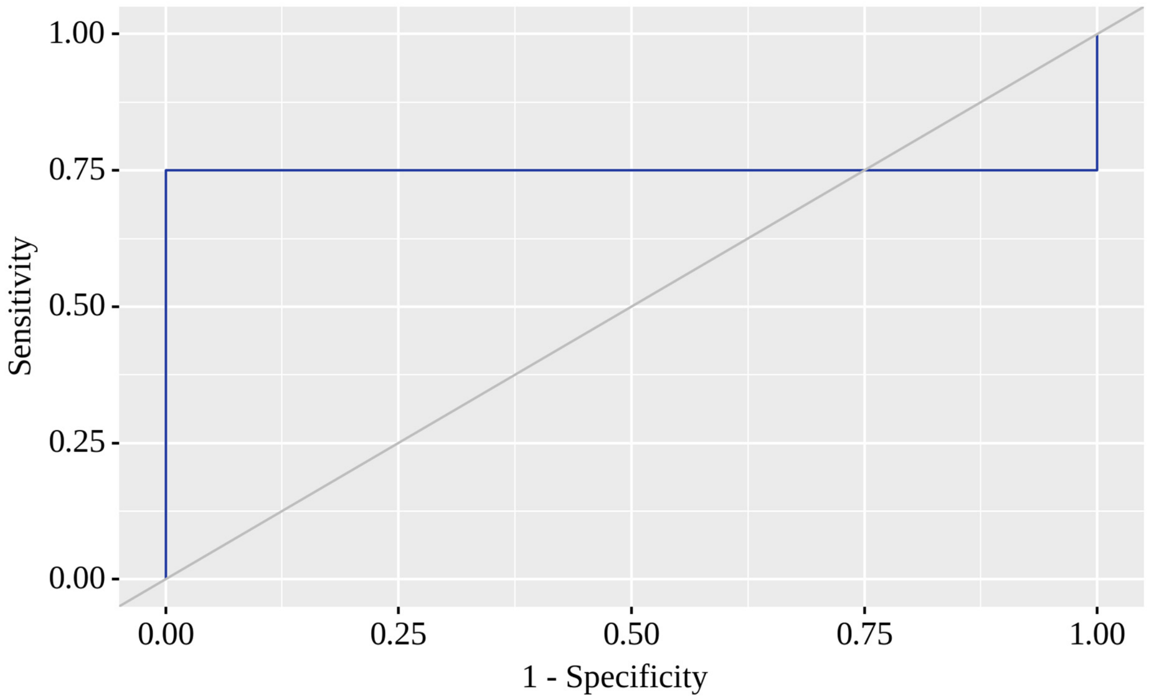

Figure 140 – ROC-curve characterizing the dependence of the probability Surgery on PDGF-β (artery) Patients

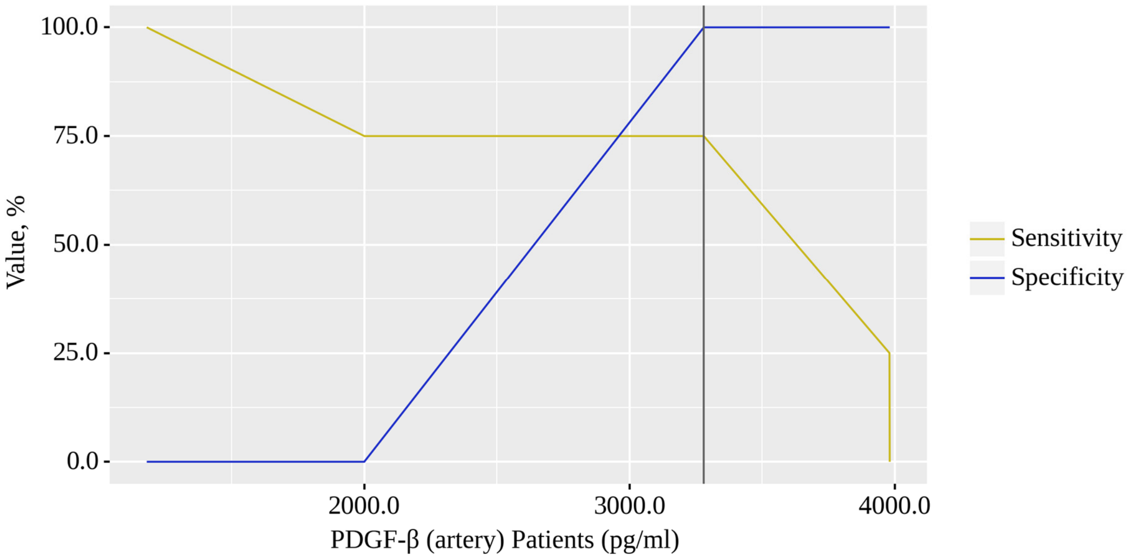

Figure 141 - Analysis of the sensitivity and specificity of Surgery depending on PDGF-β (artery) Patients

Table 101 – Threshold PDGF-β (artery) Patients

Threshold	Sensitivity (Se), %	Specificity (Sp), %	PPV	NPV
**3280**	**75.0**	**100.0**	**100.0**	**75.0**

The area under the ROC curve comprised 0.750 ± 0.196 with 95% CI: 0.366 - 1.000. The resulting model was not statistically significant (p = 0.289).

The cut-off value of PDGF-β (artery) Patients which corresponds to the highest Youden's J statistic is 3280.000 pg/ml. If PDGF-β (artery) Patients was greater than or equal to this value, operated was predicted. The sensitivity and specificity of the method were 75.0% and 100.0%, respectively.
